# Distinct patterns of structural damage underlie working memory and reasoning deficits after traumatic brain injury

**DOI:** 10.1093/brain/awaa067

**Published:** 2020-04-03

**Authors:** Amy E Jolly, Gregory T Scott, David J Sharp, Adam H Hampshire

**Affiliations:** Computational, Cognitive and Clinical Neuroimaging Laboratory, Department of Brain Sciences, Burlington Danes Building, Hammersmith Campus, Imperial College London, Du Cane Road, London W12 ONN, UK

**Keywords:** traumatic brain injury, structural connectome, working memory, reasoning, graph theory

## Abstract

It is well established that chronic cognitive problems after traumatic brain injury relate to diffuse axonal injury and the consequent widespread disruption of brain connectivity. However, the pattern of diffuse axonal injury varies between patients and they have a correspondingly heterogeneous profile of cognitive deficits. This heterogeneity is poorly understood, presenting a non-trivial challenge for prognostication and treatment. Prominent amongst cognitive problems are deficits in working memory and reasoning. Previous functional MRI in controls has associated these aspects of cognition with distinct, but partially overlapping, networks of brain regions. Based on this, a logical prediction is that differences in the integrity of the white matter tracts that connect these networks should predict variability in the type and severity of cognitive deficits after traumatic brain injury. We use diffusion-weighted imaging, cognitive testing and network analyses to test this prediction. We define functionally distinct subnetworks of the structural connectome by intersecting previously published functional MRI maps of the brain regions that are activated during our working memory and reasoning tasks, with a library of the white matter tracts that connect them. We examine how graph theoretic measures within these subnetworks relate to the performance of the same tasks in a cohort of 92 moderate-severe traumatic brain injury patients. Finally, we use machine learning to determine whether cognitive performance in patients can be predicted using graph theoretic measures from each subnetwork. Principal component analysis of behavioural scores confirm that reasoning and working memory form distinct components of cognitive ability, both of which are vulnerable to traumatic brain injury. Critically, impairments in these abilities after traumatic brain injury correlate in a dissociable manner with the information-processing architecture of the subnetworks that they are associated with. This dissociation is confirmed when examining degree centrality measures of the subnetworks using a canonical correlation analysis. Notably, the dissociation is prevalent across a number of node-centric measures and is asymmetrical: disruption to the working memory subnetwork relates to both working memory and reasoning performance whereas disruption to the reasoning subnetwork relates to reasoning performance selectively. Machine learning analysis further supports this finding by demonstrating that network measures predict cognitive performance in patients in the same asymmetrical manner. These results accord with hierarchical models of working memory, where reasoning is dependent on the ability to first hold task-relevant information in working memory. We propose that this finer grained information may be useful for future applications that attempt to predict long-term outcomes or develop tailored therapies.

## Teaser

The heterogeneity of patterns of diffuse axonal injury and cognitive impairment after TBI presents a challenge for predicting long-term outcomes. Using diffusion-weighted imaging and network analyses, Jolly *et al.* show that impairments in working memory and reasoning correlate with distinct patterns of structural damage.

## Introduction

Traumatic brain injury (TBI) can lead to a variety of cognitive problems that persist across the lifespan, impacting on quality of life and contributing to poor functional outcomes (McAllister *et al.*, 2004; Ponsford *et al.*, 2014). The role of diffuse axonal injury in the development of cognitive deficits is well established (Kinnunen *et al.*, 2011; Palacios *et al.*, 2011, 2012; Zappala *et al.*, 2012); however, our ability to accurately predict the type and severity of deficits that each patient will suffer from in the chronic phase is limited.

Brain imaging research has highlighted the importance of the structural connectome in supporting higher-order cognition and has begun to characterize the mechanisms of impairment after TBI. Studies that analyse the brain as an information processing network have motivated a more holistic perspective on cognition by illustrating that functionally specialized regions of the brain dynamically form temporary networks in order to support different cognitive processes (Beckmann *et al.*, 2005; Smith *et al.*, 2009; Hampshire *et al.*, 2016; Soreq *et al.*, 2019). The expression of these transient networks is in turn facilitated by white matter structural connectivity (van den Heuvel *et al.*, 2009). In the context of TBI, damage to structural networks produced by diffuse axonal injury has been demonstrated to disrupt functional connectivity, impairing information transfer across distal brain regions (Sharp *et al.*, 2014) and producing cognitive deficits (Kim, 2009; Bonnelle, 2012; Palacios *et al.*, 2011).

The recent application of graph theory to neuroimaging data has extended this by demonstrating that TBI alters network structure, moving it away from an optimal small-world topology where all areas can communicate with each other efficiently (Bassett and Bullmore, 2006; Braun *et al.*, 2015) to a more segregated architecture where coordinated information processing across systems is hindered (Pandit *et al.*, 2013; Caeyenberghs *et al.*, 2014; Fagerholm *et al.*, 2015). Changes to network efficiency have been proposed to be due to the loss of long-range white matter connections (Achard *et al.*, 2006; Bullmore and Sporns, 2009), supported by previous work investigating network structure and function following TBI (Pandit *et al.*, 2013; Fagerholm *et al.*, 2015). These altered structural network properties have been observed to correlate with cognitive impairments in TBI patients and can be used to robustly classify those who develop chronic cognitive problems (Fagerholm *et al.*, 2015), highlighting their potential for predicting cognitive outcomes.

The move towards describing the impact of brain injury in terms of changes to structural connectomes promises to inform our understanding of the basis of variable cognitive impairments after TBI. It also provides insights into the neural mechanisms that underlie cognitive systems. For example, deficits in working memory and reasoning are common after TBI (Kinnunen *et al.*, 2011; Palacios *et al.*, 2011, 2012; Zappala *et al.*, 2012) yet the incidence and extent of these impairments vary substantially across patients. One potential reason for this variability is the considerable heterogeneity in the spatial distribution of damage to the structural connectome after TBI. This heterogeneity is likely important when considering the impact of structural disconnection on distinct cognitive processes. Most relevantly, when in our previous studies (Hampshire *et al.*, 2012, 2019; Daws and Hampshire, 2017) we applied principal component analysis (PCA) to two large cohorts of healthy individuals (*n *=* *44 600 and *n *=* *18 455), the rotated factor solutions supported the view that working memory and reasoning form distinct cognitive components of cognitive ability. In a smaller subset of controls, it was also shown that independent component analysis of functional MRI brain activity during the performance of these tasks produces the same working memory versus reasoning component structure that relates to spatially different but partially overlapping networks in the brain (Hampshire *et al.*, 2012). Based on this, we proposed that individual differences in the functionality of these networks may manifest as distinct axes of cognitive ability; however, this interpretation was controversial (Haier *et al.*, 2014).

**Figure 1:**
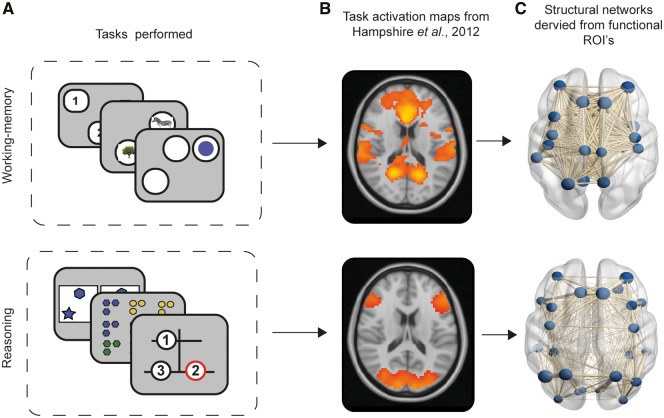
**Illustration of extraction of structural connectomes from task-activation maps produced by Hampshire *et al.* (** **2012** **).** (**A**) Cognitive tasks used, and cognitive component structure created from PCA revealing a working memory versus reasoning component structure. (**B**) Working memory and reasoning activation maps derived from task-functional MRI relative to rest in healthy controls performing the cognitive tasks. (**C**) Construction of structural connectomes in current study using activation maps from Hampshire *et al.* (2012) intersected with Desikan-Killiany grey matter atlas to define nodes (regions of grey matter) and subsequent edges between nodes as white matter connectivity.

Here, we use a novel combination of cognitive testing, diffusion-weighted imaging and graph theoretic analyses to test a logical prediction of this hypothesis, that working memory and reasoning abilities may be differentially affected by TBI dependent on the post-injury integrity of these functionally distinct networks. Critically, the impact of TBI on working memory and reasoning has seldom been investigated in parallel, yet cognitive tasks typically involve some mixture of both. Therefore, we applied PCA to data from a cohort of 92 TBI patients and 105 matched control subjects who performed six of the tasks reported in Hampshire *et al.* (2012). We sought to confirm the previously reported dissociation of behavioural factors and to extract working memory and reasoning scores in an unbiased data-driven manner. We tested whether there are deficits in both orthogonal behavioural components in the TBI population. Next, we investigated how the component scores co-vary with the integrity of the structural connectome in the TBI cohort. We took a hypothesis-driven approach, defining working memory and reasoning subnetworks of the structural connectome by intersecting the whole brain functional MRI activation maps from Hampshire *et al.* (2012) (Fig. 1) with an established atlas of the white matter tracts that connect the grey matter regions of the brain. We tested whether working memory and reasoning performance have dissociable relationships to variability in information processing properties, as quantified using graph theory measures of connectivity (degree centrality), integration (global efficiency) and modularity (local efficiency, clustering coefficient), of their associated structural subnetworks. We sought evidence of a dissociation in a purely data-driven manner by using canonical correlation analysis to identify modes that relate variability in working memory and reasoning performance to white matter measures across the whole structural connectome. We then further examined this dissociation using machine learning models to predict working memory and reasoning performance from the functional subnetworks and graph theory measures. We extended previous findings that disconnection of network hubs (highly interconnected regions) have the strongest association with cognitive performance (Fagerholm *et al.*, 2015), by demonstrating that this relationship is multivariate and dissociable for the functionally distinct subnetworks of the structural connectome. Finally, we determined the relationship that exists between white matter measures, component scores from our computerized tests, and the measures provided by neuropsychological tests that are widely used to assess cognitive problems in the clinical setting.

## Materials and methods

### Participants

Data from 92 patients [19 female, mean age 42.9, standard deviation (SD) 12.40 years] with a moderate-severe TBI as classified using the Mayo criteria (Malec *et al.*, 2007) were included in this study. Patients were recruited from neurology clinics in London and were within the chronic phase (at least 6 months post-injury). Mean time since injury was 130.4 months (SD 154, range 6–497 months). MRI findings, mechanism of injury and current medications can be found in Supplementary Table 1. Inclusion criteria were: age 18–80 years (range 20–80 years), no significant neurological or psychiatric history or previous TBI, no history of alcohol or substance abuse and able to understand English. Exclusion criteria included: contraindication to MRI or a positive urine drug screen. Written informed consent was obtained from all patients in accordance with the Declaration of Helsinki. The studies were approved by the West London and GTAC Research Ethics Committee (14/LO/0067, 13/LO/1678, 14/LO/1998). All patients completed structural MRI, a computerized cognitive battery and a standard neuropsychological test battery.

One-hundred and five healthy controls were included (35 female, mean age 44.98, SD 15.16 years). Controls had no history of neurological or psychiatric illness, TBI or alcohol and substance abuse. All participants gave written informed consent. All participants completed structural MRI. A subset (*n *=* *35, eight females, mean age 44, SD 13.05) also completed the computerized cognitive battery and standard neuropsychological tests.

### Structural MRI acquisition

Structural MRI was acquired using a 3 T Siemens Magnetom Verio Syngo scanner with a 32-channel head coil. All participants were scanned using the same acquisition parameters. Structural MRI included a high-resolution T_1_-weighted MPRAGE (106 1-mm thick transverse slices, repetition time = 2300 ms, echo time = 2.98 ms, flip angle = 9°, in-plane resolution = 1 × 1 mm, matrix size = 256 × 256, filed of view = 25.6 × 25.6 cm), diffusion weighted imaging (64 directions, b = 1000 s/mm^2^ with four interleaved b = 0 s/mm^2^, echo time/repetition time = 103/9500 ms, 64 contiguous slices, field of view 256 mm, voxel size 2 mm^3^) and FLAIR to identify focal lesions.

### Neuropsychological assessment

#### Computerized cognitive assessment

**Figure 2:**
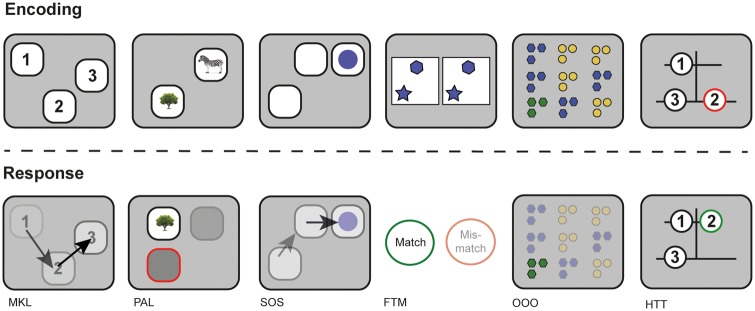
**Schematic of the six computerized cognitive tasks used to assess TBI patients and controls.** From *left* to *right*: working memory tasks MKL, PAL, SOS; and reasoning tasks FTM, OOO, and HTT. Encoding demonstrates the initial presentation of stimuli for each task and response demonstrates the method in which to complete the task correctly.

A computerized battery was used to assess working memory and reasoning in TBI patients and healthy controls (Fig. 2). This battery consisted of six tasks, designed and programmed by A.H.H. and based on classical assessment paradigms to measure working memory and reasoning performance. These were identified as the minimal set required to reliably differentiate working memory and reasoning abilities based on subsampling data from previous studies with two large cohorts of internet users (Hampshire *et al.*, 2012, 2019; Daws and Hampshire, 2017). Tasks that loaded onto a third language factor were excluded because of the diverse backgrounds of our cohort. The tasks were deployed on iPads via a custom-programmed application. They included: (i) Monkey Ladder test (MKL), a visuospatial working memory task based on non-human primate research (Inoue and Matsuzawa, 2007); (ii) Paired Associates Learning (PAL), an object-spatial variant on the binding paradigm that has previously been used in other clinical populations (Gould, 1981) and in TBI patients with post-traumatic amnesia (De Simoni *et al.*, 2016); (iii) Self-Ordered Search (SOS), a visuospatial working memory task developed from behavioural measures of strategy in searching (Collins *et al.*, 1998); (iv) Feature Match Test (FTM) based on a classical feature search task used to measure attentional processing (Treisman and Gelade, 1980); (v) Odd One Out (OOO), a deductive reasoning task based on a subset of problems from the Cattell Culture Fair Intelligence Test (Cattell, 1949); and (vi) The Hampshire Tree Task (HTT), a spatial planning task designed by A.H.H. and related to the Tower of London Task (Shallice, 1982).

All tasks were adaptive to performance, e.g. increasing and decreasing the speed of delivery or number of stimuli, or the complexity of problems on completion of each trial. For tasks that did not have time limits, three errors triggered the end of the task. Detailed information about the task designs has been reported previously (Hampshire *et al.*, 2012) and can be found in the Supplementary material.

### Standard neuropsychological assessment

In addition to computerized testing, all TBI patients and 35 healthy control subjects completed a standard pen and paper neuropsychological assessment that is commonly used to assess cognitive function after TBI (Kinnunen *et al.*, 2011; De Simoni *et al.*, 2018; Jenkins *et al.*, 2018). This provides comparative, clinically validated measures enabling us to determine the relevance of our computerized tasks to the clinical gold standard. The test battery included: (i) the Wechsler test of Adult reading (WTAR) and the matrix reasoning subtest from the Wechsler Adult Intelligence Scale (WAIS-III) to assess intellectual function (Wechsler, 1945); (ii) the Peoples Test from the Peoples And Door Test (Wechsler, 1945) and logical memory subtest of the Wechsler Memory Scale (Wechsler, 1945) to assess memory; and (iii) the colour-word interference (Stroop) test from the Delis-Kaplan executive function system (DKEFS; Delis *et al.*, 2001) and Trail-Making Task (A and B) to assess executive function.

### Statistical analysis

#### Diffusion tensor imaging

Diffusion-weighted images were processed using FSL’s diffusion toolkit in the following steps. First, eddy current correction was applied to diffusion weighted images to correct for distortions and movement. BVECs were rotated using the output from eddy correction and brain extraction (BET) was applied to the eddy-corrected data. Tensor fitting was performed using DTIFIT with a weighted least squares approach. Individual fractional anisotropy maps constructed from DTIFit were registered to standard MNI152 space using the FMRIB 1 mm fractional anisotropy atlas and tract-based spatial statistics (TBSS) (Smith *et al.*, 2006). Concatenated standard space fractional anisotropy maps of TBI patients and controls were skeletonized at 0.2 threshold to sample from the centre of the white matter in order to avoid partial volume effects.

Voxelwise non-parametric permutation analysis was undertaken using ‘randomise’ in FSL to examine patterns of white matter damage (as quantified using fractional anisotropy) in the TBI cohort relative to controls. Specifically, fractional anisotropy skeleton maps were compared between patients and controls in a voxelwise manner with 10 000 permutations forming the null distribution. Age was included as a covariate and results were threshold-free cluster corrected to account for multiple comparisons.

#### Behavioural data

Cognitive data for TBI patients and controls were preprocessed in the following steps. Multiple linear regression was applied to regress out age, age-squared and age-cubed from the data, accounting for non-linear relationships between age and cognitive performance. A rank based inverse transform was then applied to the extracted residuals to ensure assumptions of normality were met. All preprocessing and analyses were performed in MATLAB (R2017b). For computerized cognitive data, performances across the six tasks were compared between TBI patients and controls using a repeated measures ANOVA. Performance across the 16 paper neuropsychological test measures were also compared using a repeated measures ANOVA. To confirm in an unbiased data-driven manner that the six computerized tasks mapped onto working memory and reasoning components as per previous research (Hampshire *et al.*, 2012), a PCA with varimax rotation was applied to patients’ task scores. A PCA with varimax rotation was also applied to patient and control data combined to determine whether a similar factor structure was observed. The Kaiser convention was used to threshold out components that had eigenvalues < 1 prior to rotation.

Bivariate correlations were performed to determine the degree to which the computerized tasks captured the same variance in patients as standard neuropsychological assessment. For dimensionality reduction, age-regressed and rank inverse normalized data from patients using the standard neuropsychological battery were included in a PCA with varimax rotation. Components that had eigenvalues < 1 prior to rotation were removed. The components that remained were then correlated to the components derived from the computerized tasks and false discovery rate (FDR) corrected for multiple comparisons. A PCA with varimax rotation was also applied to patient and control neuropsychological data combined to determine whether a similar factor structure was observed.

### Graph theory analysis

**Fig 3:**
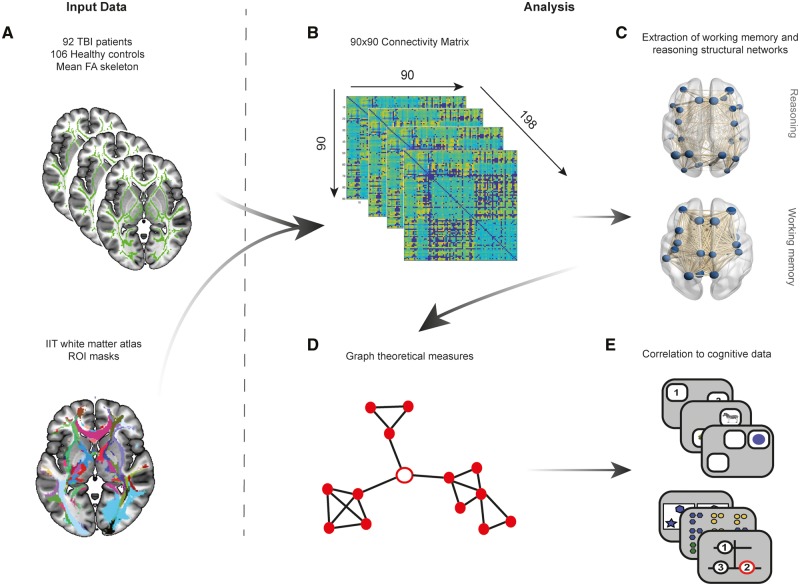
**Overview of graph theory analysis methods.** (**A**) Skeletonized fractional anisotropy (FA) maps for the 92 TBI patients and 106 healthy controls were intersected with region-to-region, thresholded and binarized track density images (TDIs) created using probabilistic tractography for the IIT white matter atlas. Mean fractional anisotropy for each region of interest (ROI) was therefore extracted for all patients and controls. (**B**) Mean fractional anisotropy for each region of interest derived from the 90 region-to-region connections were then used to create a 90 × 90 connectivity matrix for all 198 subjects and weighted by number of streamlines to account for weak connections. A thresholding approach using the mean and standard deviation of healthy controls for each connection was then applied to all controls and patients to remove weak connections. (**C**) Using task-functional MRI activation maps from Hampshire *et al.* (2012), working memory and reasoning structural networks were derived from the 90 × 90 connectivity matrices by selecting nodes that overlapped with activation maps and their respective connections. (**D**) Graph theoretical measures were subsequently derived from these two structural networks including global/local efficiency, degree centrality and clustering coefficient. (**E**) Graph theory metrics for each network were then correlated to performance on cognitive tasks associated with working memory and reasoning in patients.

A summary of the analysis methodology applied to our graph theory analysis can be found in Fig. 3. To investigate structural network topology and efficiency, a graph theoretical approach was applied to the skeletonized fractional anisotropy maps of patients and controls. As tractography is susceptible to issues when fractional anisotropy values are low (Squarcina *et al.*, 2012), we used a large set of predefined white matter tractography masks from healthy controls to define region-to-region connectivity for the creation of connectivity matrices. This was achieved using the track density images (TDIs) created for the construction of the Illinois Institute of Technology (IIT) human brain atlas of white matter (Zhang and Arfanakis, 2018) developed by the Magnetic Resonance Imaging Laboratory (MRIIT). In summary, track density images (standardized to MNI152 space) of 72 healthy controls were created by MRIIIT by first parcellating structural T_1_ images into 90 cortical and subcortical grey matter regions using the Desikan-Killiany grey matter parcellation scheme (Desikan *et al.*, 2006). Individual grey matter parcellations of the 72 healthy controls were registered to standard MNI152 space and probabilistic tractography between all 90 regions was performed creating TDIs for each region to region connection where they existed. TDI, region labels, number of streamlines and the IIT white matter atlas were obtained from an online archive (https://www.nitrc.org/projects/iit/). Refer to Zhang and Arfanakis (2018) for more details.

Each region-to-region connection (TDI) from the IIT atlas was first thresholded and binarized to create a region of interest mask. Masks were intersected with each participant’s skeletonized fractional anisotropy map to extract the mean fractional anisotropy for that connection (Fig. 3A). This resulted in a 90 × 90 undirected connectivity matrix, weighted by fractional anisotropy, for each participant (Fig. 3B). When no connection was present between regions in the connectivity matrix, a value of zero was included.

### Connectivity matrix preprocessing

We applied the same approach as reported by Fagerholm *et al.* (2015) when preprocessing our structural connectivity matrices. The aim was to ensure results were robust against different choices of thresholding whilst controlling for weak connections (i.e. small tracts with low fractional anisotropy), which can increase noise and impact upon graph theoretical analysis (Li *et al.*, 2013). The following steps were applied: first, each connection in a participant’s connectivity matrix was multiplied by the number of streamlines used to create the connection during probabilistic tractography to ensure greater weight was applied to large white matter tracts. Second, the overall mean and standard deviation of each connection in the 90 × 90 connectivity matrices of all 105 healthy controls was calculated. This produced two 90 × 90 connectivity matrices representing the average fractional anisotropy and standard deviation of each connection in our healthy population. Next, a total of 10 thresholding limits were calculated using the same scheme as Fagerholm *et al.* (2015) ranging from 0.5 to 5 SD units in increments of 0.5. This was achieved by multiplying the control SD connectivity matrix by the threshold value (e.g. 0.5 or 5) and subtracting this from the control mean connectivity matrix. The resulting threshold values derived from this calculation were then applied to each patient and control’s individual connectivity matrix such that any value less than the threshold value calculated for a connection was replaced with a zero. Finally, this process was repeated for each of the 10 possible thresholds. For ease of presentation, all findings are presented using the moderate threshold (2.5 SD units) with all other thresholding results reported within the Supplementary material.

### Extraction of reasoning and working memory networks for graph theory analysis

To test the hypothesis that impairments of working memory and reasoning performance relate to disruption of different systems in the brain, we focused our graph theory analysis on the networks that had previously been associated with these two aspects of cognition when using the same tasks (Hampshire *et al.*, 2012). Specifically, we extracted the whole brain working memory and reasoning activation maps from a study that measured changes in functional MRI activation for the same tasks relative to rest (Hampshire *et al.*, 2012). This rendered two distinct networks associated with reasoning and working memory (Fig. 3C). The DTI connectivity matrices were intersected with each of these maps, so that two subnetworks were produced comprising nodes that overlapped with the activation maps and their connections (edges).

### Graph theory measures

Graph theoretic measures were extracted from the structural connectivity matrices of reasoning and working memory networks using functions from the brain connectivity toolbox (BCT) (Rubinov and Sporns, 2010) (Fig. 3D). Hubs within each network were identified by determining the top 20% of nodes with the highest average degree within healthy controls (van den Heuvel *et al.*, 2009) to determine whether dysconnectivity of network hubs had a strong relationship to cognitive impairment as per previous research (Fagerholm *et al.*, 2015). Global and local properties of each network for patients and controls were examined. For global measures, the metrics extracted were: global efficiency, average local efficiency, and average degree centrality. For local measures, the metrics extracted were: local efficiency, degree centrality, and local clustering coefficient. Measures were selected based on previous literature examining TBI (Pandit *et al.*, 2013; Fagerholm *et al.*, 2015) as well as for the quantification of network integration (global efficiency, average degree centrality), modularity (local efficiency, clustering coefficient) and disconnection (degree centrality). For example, TBI is known to disrupt small-world topology via diffuse axonal injury (Sharp *et al.*, 2014), therefore degree centrality (a measure of dysconnectivity) was used to examine the loss of long-range white matter connections. Higher-order cognition is supported by small-world topology (Bullmore and Sporns, 2009; Braun *et al.*, 2015; Cohen and D'esposito, 2016), which refers to an optimal balance between network segregation (quantified using local efficiency and clustering coefficient) and integration (quantified using global efficiency). These measures have previously been shown to be sensitive to TBI (Pandit *et al.*, 2013; Fagerholm *et al.*, 2015) and provide a focused summary of network topology in the brain. Further information about these metrics can be found in the Supplementary material.

### Multivariate correlational analysis of reasoning/working memory performance and structural networks

Canonical correlational analysis (CCA) was applied as a preliminary statistical analysis to confirm that multiple statistically significant latent variables relate network dysconnectivity (as quantified using degree centrality) to working memory and reasoning impairment. This method allows the dimensionality of multivariate relationships between two sets of variables to be statistically tested in a data-driven manner (see Supplementary material for a detailed description of CCA). For our analysis, we explored the relationships between behavioural data and degree centrality of all 90 nodes that were defined within our whole-brain structural connectome. To overcome the problem of overfit due to the large number of nodes, a PCA was applied prior to CCA reducing node measures to a set of components that captured 90% of the variance. The resultant component scores were input to the CCA along with the patient’s cognitive component scores. The relationships between these two sets of variables (behavioural and graph theoretic) are referred to as canonical modes and the strength of their relationships are represented as canonical correlation coefficients. If more than one canonical correlation coefficient (or mode) is significant as calculated using robust permutational testing, evidence of multiple, differential relationships between behaviour and structural data can be inferred. To determine the relative contribution of the two sets of variables to each mode, back projection of canonical variates to raw task and node degree data using Pearson’s *r* correlation was applied. A custom subsampling routine with separate train and test data was applied to robustly determine the generalizability of canonical model and degree of overfit (see Supplementary material for details). In summary, the CCA analyses were repeated in an iterative loop, whereby the data were randomly subsampled, the model trained, then applied to the held out data to which it was naïve. This process was repeated with systematic variation in the trained (number of samples) and held out (remaining samples) ratio, with *r*-values averaged over 1000 iterations at each point. The process was then repeated using permuted data to assess overfit.

### Cross-sectional and correlational analysis of graph theory measures

Global network measures were compared between patients and controls using *t*-tests to determine whether there were significant differences in the overall information processing properties of the structural connectome. Data were checked for assumptions of normality and results FDR corrected across the 12 comparisons. Next, to determine whether there was any relationship between network properties and corresponding cognitive performance in patients, the global graph theoretic measures of reasoning and working memory networks were correlated to reasoning and working memory cognitive components derived from patient-only PCA (Fig. 3E). These comparisons were repeated across non-corresponding network-behavioural component pairs, e.g. global properties of the working memory network were correlated to the reasoning cognitive component and global properties of the reasoning network were correlated to the working memory cognitive component. Results were corrected for multiple comparisons across the 12 correlations using FDR correction.

Local network measures were also compared between patients and controls using *t*-tests to determine if there were any significant differences in the properties of individual nodes within the structural connectome. Cross-sectional local network measures were corrected for multiple comparisons across network nodes using FDR correction. Local measures of the nodes in the working memory and reasoning networks were then correlated to reasoning and working memory cognitive component scores derived from patient-only PCA to quantify the relationship between node properties and cognitive performance in patients. Similar to global network analysis, correlations were repeated across non-corresponding network-behavioural component pairs. For each network, correlational results were corrected for multiple comparisons across network nodes using FDR correction.

### Prediction of reasoning and working memory performance using local/nodal graph theory measures 

Although CCA is suitable for identifying how many latent variables interrelate two multivariate datasets, a limitation is that the models tend to be overfitted. Consequently, machine learning regression was used to investigate the predictive value of local/nodal graph theory measures derived from each network for working memory and reasoning performance in patients. Using the Pattern Recognition for Neuroimaging toolbox (PRoNTo; Schrouff *et al.*, 2013), local/nodal graph theory measures from a given network were used as independent variables and the component score for either working memory or reasoning was used as the dependent variable in a kernel ridge regression (KRR) model with automated hyperparameter optimization using nested 5-fold cross validation. For example, local/nodal measures of degree centrality from the working memory network were used in a model to predict working memory performance and in another model to predict reasoning performance. This process was repeated for all graph theory measures in each network.

Model validation was achieved by running 5-fold cross validation on the data after hyperparameter optimization to determine accuracy and generalizability. This was followed by permutation testing with 1000 randomizations to derive a null distribution and *P*-value for each model. Model accuracy was assessed using Pearson correlation coefficients between network local/nodal graph theory measures and cognitive component scores. The proportion of variance explained (R^2^) and mean standard error (MSE) between predicted and actual component score was also examined. As an additional step, computation of weights for each model was calculated to examine which nodes within a network contributed the greatest to the prediction model.

### Data availability

Raw data were generated at Imperial College London. Derived data supporting the findings of this study are available from the corresponding author on request.

## Results

### TBI patients perform significantly less well on all six cognitive tasks

**Figure 4:**
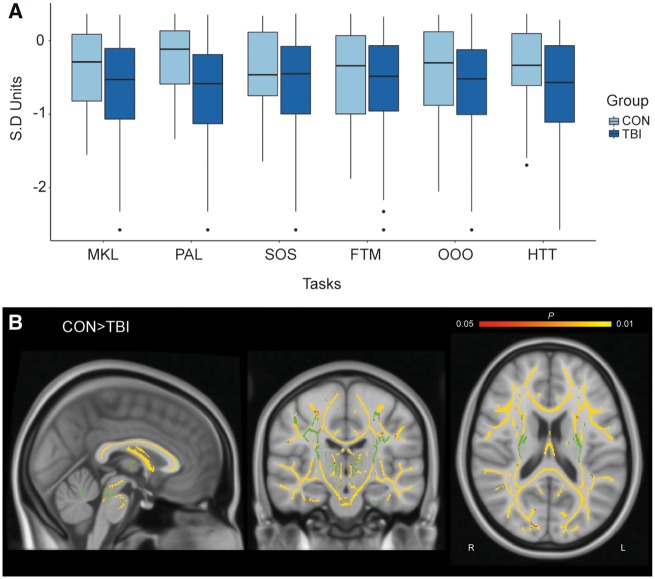
**Cross-sectional analysis of TBI patients and controls for cognitive performance and whole-brain fractional anisotropy.** (**A**) Performance across the six cognitive tasks in TBI patients and healthy controls. *Y*-axis is in SD units. (**B**) Voxelwise analysis using TBSS, TFCE corrected with age as a covariate. Yellow indicates voxels with significantly higher fractional anisotropy in controls than patients (*P *<* *0.05). Green indicates the group averaged mean fractional anisotropy skeleton mask.

Repeated measures ANOVA across the six cognitive tasks revealed a significant main effect of group [*F*(1,126) = 10.554, *P = *0.001] such that TBI patients performed significantly less well than healthy controls. There was no significant group × task interaction indicating that all tasks could be affected by TBI to similar degrees, although numerically the PAL showed the greatest sensitivity (Fig. 4A).

### TBI patients have widespread diffuse axonal injury

Voxelwise TBSS indicated that patients on average had significantly lower fractional anisotropy compared to controls, threshold-free cluster enhancement (TFCE) corrected (*P < *0.05). White matter damage was distributed widely throughout the brain (Fig. 4B). No significant increases in fractional anisotropy were observed in patients.

### Cognitive tasks load onto two distinct cognitive components

**Figure 5:**
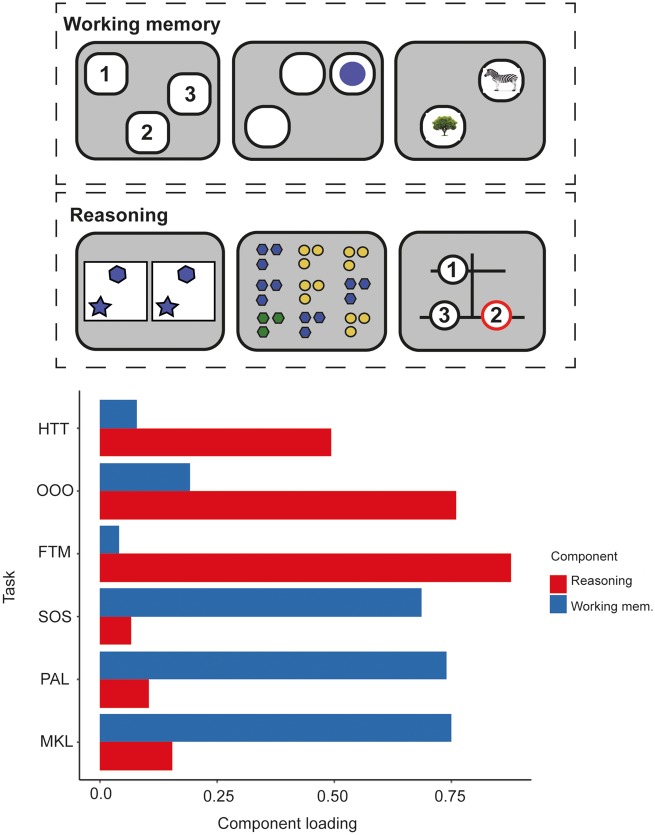
**Principal component analysis of patient cognitive data.** Component loadings for each task demonstrate higher loadings onto component 1 for reasoning-based tasks such as the HTT, OOO and FTM. Greater loadings to component two were observed in working memory-based tasks including MKL, PAL and SOS.

Applying the Kaiser convention, a PCA of the patient cognitive data indicated two significant components (Supplementary Fig. 1). Orthogonal (varimax) rotation replicated the previously reported pattern of task-component loadings, where psychometric analyses were applied to data from 44 600 members of the general public (Hampshire *et al.*, 2012) (Fig. 5). The rotated components explained 54.27% of the variance with component one explaining 27.17% and component two explaining 27.10%. Higher loadings of the FTM, OOO and HTT tasks onto component one accord with a distinct reasoning ability. Higher loadings of the MKL, PAL and SOS onto component two accord with a distinct working memory ability. PCA with varimax rotation was also applied to all patient and control cognitive data combined. A similar structure was observed with two components explaining 42.33% of the variance in the data. Component one explained 21.17% and component two explained 21.16%. Component one again had highest loadings for the reasoning tasks (FTM, OOO, HTT) while component two had the highest loadings for the working memory tasks (MKL, PAL, SOS).

### TBI patients perform significantly less well on a number of standard neuropsychological tests

Repeated measures ANOVA did not reveal a significant main effect of group [*F*(1,15) = 3.652, *P = *0.056]. However, a significant group × task interaction was observed [*F*(15,1856) = 12.920, *P < *0.001] (Supplementary Fig. 2A) with *post hoc t*-tests revealing that this interaction was driven by lower performance on 12 of 16 test scores. This included the WTAR, Trail Making Test scores, the four subtests of the colour-word (Stroop) interference task, Peoples Test total score, Peoples Test delayed score, logical memory total score for subtests one and two and logical memory retention scores (Supplementary Table 2).

### Reasoning and working memory tasks correlate to standard clinical neuropsychological tests

We examined the relationship between the two cognitive components derived from our computerized tasks and the standard paper neuropsychological tests in patients. A PCA of standard neuropsychological tests revealed four significant components that explained 69.93% of the total variance (Supplementary Fig. 2B). A PCA with both patients and controls revealed the same factor structure. Bivariate correlations between computerized and neuropsychological cognitive components revealed a significant relationship between reasoning and neuropsychological component one (*r =* −0.32, *P = *0.016, FDR corrected) and a significant relationship between working memory performance and neuropsychological component three (*r = *0.37, *P < *0.01, FDR corrected) (Supplementary Fig. 2C). Task loadings revealed that component one was primarily associated with executive function measures such as the colour-word interference task subscales (Stroop) and trail making tasks whilst component three was associated with memory measures such as the Peoples Test and to a lesser extent, logical memory subscores (Supplementary Fig. 2D).

### Abnormal graph theory measures of white matter and reasoning networks after TBI

Extraction of working memory and reasoning networks by intersection of the Desikan-Killiany grey matter atlas with task-based activation maps from Hampshire *et al.* (2012) generated a working memory network consisting of 31 nodes and a reasoning network consisting of 26 nodes (Supplementary Fig. 3). In network science, nodes can be members of multiple networks. Here, there were nine nodes that had shared membership across the two networks (Supplementary Table 3). These included: left lateral orbitofrontal, left precentral gyrus, left rostral middle frontal, left superior frontal, right caudate middle frontal, right precentral, right rostral middle frontal, right superior frontal and right superior parietal lobe. Six hubs were identified in the working memory network based on average degree in healthy controls and included: left caudate, right pallidum, insula bilaterally, right precuneus and the right superior parietal cortex. In the reasoning network, five hubs were identified and included: the left rostral middle frontal cortex, left superior frontal cortex, superior parietal cortices and right inferior parietal cortex. Three of the hubs within the reasoning network formed nodes within the working memory network. Hub classification was consistent across all analysed thresholds.

Cross-sectional analysis of global network measures using graph theory demonstrated that TBI patients had significant alterations in both working memory and reasoning networks compared to controls. TBI patients had significantly lower global efficiency in working memory [*t*(196) = 6.04, *P < *0.001, FDR corrected] and reasoning networks [*t*(196) = 5.29, *P < *0.001, FDR corrected], significantly lower average degree centrality in working memory [*t*(196) = 8.82, *P < *0.001] and reasoning networks [*t*(196) = 8.81, *P < *0.001, FDR corrected] and significantly higher average local efficiency in working memory [*t*(196) = −6.59, *P < *0.001, FDR corrected] and reasoning networks [*t*(196) = −3.35, *P < *0.001, FDR corrected] compared to controls. This was consistent across 80% of threshold iterations (Supplementary Fig. 4) excluding the most extreme thresholds only.

### Patients demonstrate alterations in local/nodal network properties

**Figure 6:**
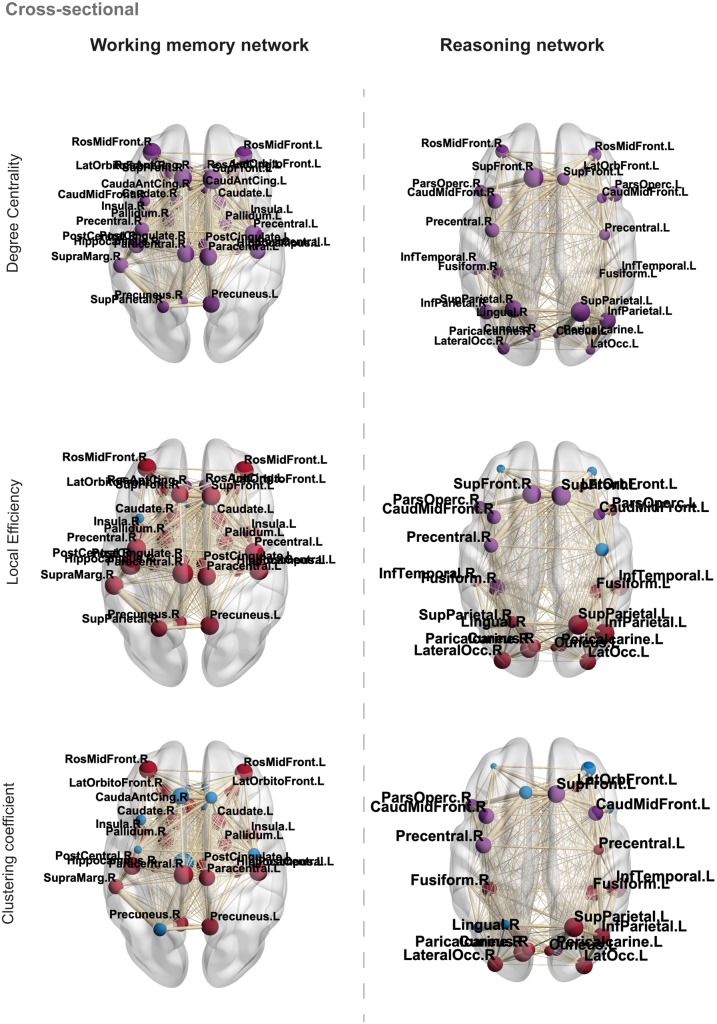
**Cross-sectional analysis of local/nodal properties in TBI patients and controls.** Blue nodes indicate non-significance, purple nodes indicate significantly lower values in patients compared to controls and red nodes indicate significantly greater values in patients compared to controls, FDR corrected. Node size = difference between patient and control local/nodal properties for each metric examined. Edge width = weighted by fractional anisotropy and no. streamlines. Significant nodes are labelled for reference.

Individual node metrics in both the working memory and reasoning networks were compared between patients and controls (Fig. 6). Patients demonstrated significant reductions in degree for all nodes within the working memory and reasoning networks compared to controls (*P < *0.001, FDR corrected). In the working memory network, local efficiency was significantly higher in 81% of nodes (*P < *0.01, FDR corrected) and significantly lower in 6% of nodes (*P < *0.01, FDR corrected) compared to controls. Greater clustering coefficients were also observed in 68% of nodes (*P < *0.05, FDR corrected) in patients compared to controls (Supplementary Table 4).

In the reasoning network, local efficiency was significantly greater in 62% of nodes (*P < *0.05, FDR corrected) and significantly lower in 23% of nodes (*P < *0.05, FDR corrected) in patients compared to controls. Clustering coefficient was significantly greater in 50% of nodes (*P < *0.01, FDR corrected) and significantly lower in 19% of nodes (*P < *0.01, FDR corrected) in patients compared to controls (Supplementary Table 5).

### CCA confirms dissociable relationships between working memory/reasoning and structural networks

**Figure 7:**
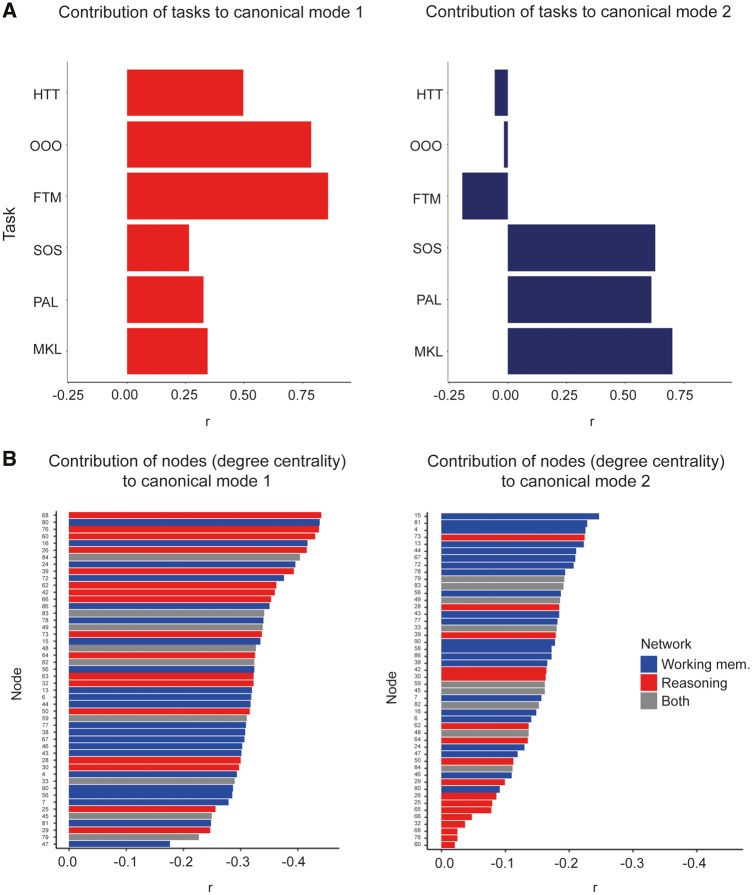
**Back projection of canonical variates to task and node data from canonical correlation analysis.** Canonical correlation analysis produced two significant modes demonstrating two distinct relationships between cognitive variables and degree centrality of the 90 nodes defined in the whole brain connectome. (**A**) Back projection of cognitive canonical variates to cognitive tasks demonstrated greater contribution of reasoning tasks (HTT, OOO and FTM) to canonical mode 1 and greater contribution of working memory tasks (MKL, PAL, SOS) to canonical mode 2. (**B**) Back projection of node canonical variates to individual nodes and their measures of degree centrality revealed that dissociable patterns of nodes were associated with each canonical mode. Specifically, reasoning nodes (red bars) contributed the most to canonical mode 1 and working memory nodes (blue bars) contributed the most to canonical mode 2. Grey bars denote where nodes are present within both structural networks.

A CCA was conducted to test in a data-driven manner whether multiple latent variables captured shared variance between node degree centrality (a graph theoretic measure of white matter dysconnectivity) and cognitive measures. Data reduction using an unrotated PCA was applied to the 90 nodes included in the whole brain structural connectome. A total of nine components capturing 90% of the variance were identified. Scores for the two cognitive components formed the first matrix whilst scores for the nine node components formed the second matrix input to the CCA. To determine the significance of canonical correlation coefficients, a permutational test was conducted, where the subject labels were shuffled and the CCA repeated 10 000 times, producing null distributions for each canonical mode (Supplementary Fig. 5). The results indicated two significant modes (*P < *0.001 and *P < *0.02) with canonical coefficient values (*r*) of 0.470 and 0.334, respectively. The means of canonical modes in the corresponding null distributions were (*r*) of 0.36 and 0.24, respectively. Back projection of canonical modes to raw task and node degree data revealed a greater contribution of reasoning tasks to mode one and greatest contribution of working memory tasks to canonical mode two (Fig. 7A). Of the 90 nodes included within the analysis, back projection revealed nodes associated with the reasoning subnetwork contributed the greatest to canonical mode one whereas nodes associated with the working memory subnetwork were found to contribute the greatest to canonical mode two (Fig. 7B).

### Correlation of global network properties with cognitive performance in patients

CCAs are useful insofar as they test how many latent variables relate two matrices of data in a data-driven and multivariate manner. A limitation is that they are overfitted by definition and provide limited mechanistic insight. Therefore, calculation of the scale of relationships and interpretation require further analysis. First, global graph theoretic measures from the two subnetworks were compared to behavioural scores. The results showed significant but distinct relationships to cognitive performance. Specifically, global efficiency of the working memory network correlated significantly and at the same approximate strength with both working memory (*r = *0.22, *P = *0.042, FDR corrected) and reasoning (*r = *0.25, *P = *0.025, FDR corrected) performance. Conversely, global efficiency of the reasoning network correlated significantly with reasoning (*r = *0.32, *P = *0.024, FDR corrected) performance, but was subthreshold for working memory (*r = *0.20, *P = *0.06, FDR corrected). Similarly, the mean local efficiency of the working memory network correlated significantly with both working memory (*r =* −0.25, *P = *0.025, FDR corrected), and reasoning (*r =* −0.26, *P = *0.024, FDR corrected) performance; whereas the average local efficiency of the reasoning network correlated significantly with reasoning (*r =* −0.28, *P < *0.02, FDR corrected) but not working memory (*r =* −0.12, *P = *0.239, FDR corrected) performance. Finally, mean degree centrality of the working memory network correlated at the same approximate strength with working memory (*r = *0.28, *P < *0.02, FDR corrected) and reasoning (*r = *0.30, *P < *0.02, FDR corrected); whereas average degree centrality in the reasoning network correlated more robustly with reasoning (*r = *0.33, *P < *0.02, FDR corrected) than working memory performance (*r = *0.24, *P = *0.025, FDR corrected).

### Local/nodal level network properties correlate to cognitive performance in patients differentially

**Figure 8:**
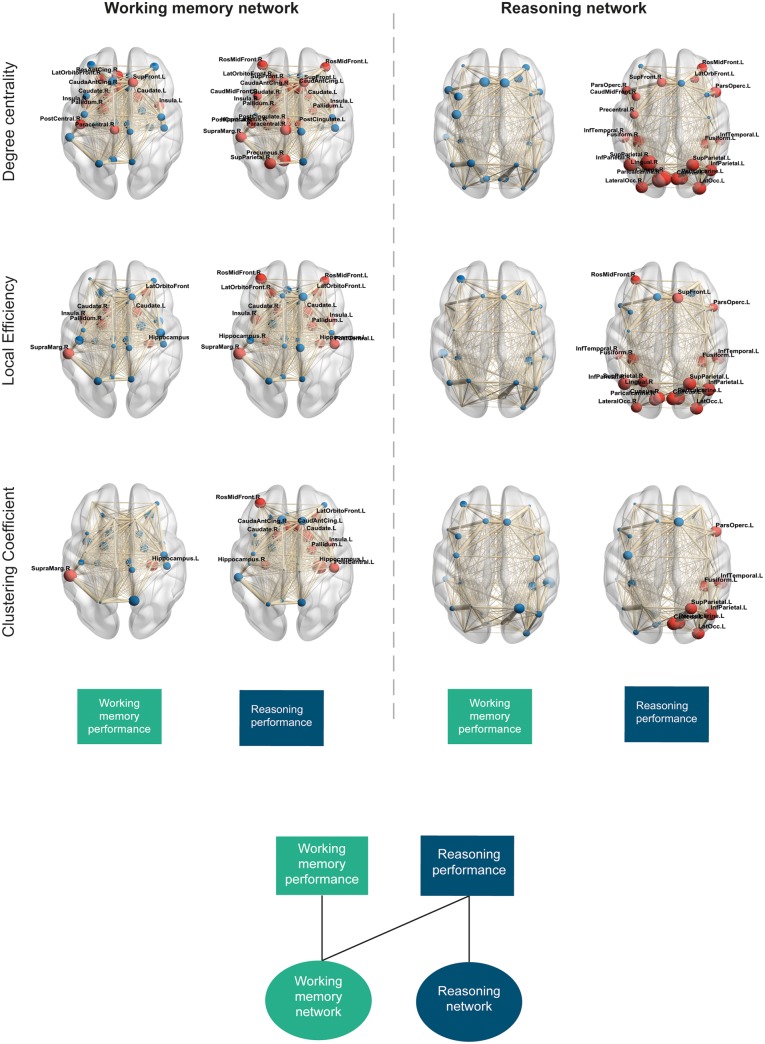
**Local graph theory network measures and correlation to cognitive performance.** Node size represents correlation coefficient (*r*, positive or negative). Red nodes demonstrate a significant correlation to cognitive components (*P *<* *0.05, FDR corrected), blue nodes demonstrate non-significant correlations. For measures of degree centrality, red nodes denote significant positive correlations, FDR corrected. For measures of local efficiency and clustering coefficient, red nodes denote significant negative correlations, FDR corrected. Edge thickness demonstrates weight of a given connection (fractional anisotropy and number of streamlines). Boxes at the *bottom* demonstrate which cognitive component is correlated to each of the networks and properties demonstrated. *Bottom* panel illustrates the dissociable relationship between cognitive components and working memory and reasoning structural network properties.

Next, local/individual node level graph properties were correlated to cognitive performance in patients (Fig. 8). In the working memory network, significant positive correlations were observed between working memory performance and degree centrality in 32% of nodes. Significant negative correlations were observed between working memory performance and local efficiency in 20% of nodes, whilst significant negative correlations were demonstrated between clustering coefficient and working memory performance in 3% of nodes (*P < *0.05, FDR corrected). Correlation of reasoning performance to working memory network properties showed significant positive correlations with degree centrality in 70% of nodes and negative correlations to local efficiency and clustering coefficient in 42% and 35% of nodes, respectively (*P < *0.05, FDR corrected). Local efficiency and degree centrality of network hubs including the left caudate, right pallidum and insula bilaterally were shown to be consistently related to working memory and reasoning performance. Nodes with significant correlations to reasoning and working memory performance were all shown to have abnormal network properties compared to controls.

In the reasoning network, significant positive correlations were observed between degree centrality and reasoning performance in 81% of nodes. A mixture of positive (62%) and negative (8%) correlations was observed between reasoning performance and local efficiency of nodes. Significant negative correlations were also observed between reasoning performance and clustering coefficient in 31% of nodes (*P < *0.05, FDR corrected). In contrast, no significant correlations were observed between working memory performance and node properties in the reasoning network. Four of the five network hubs consistently related to reasoning in both degree centrality and local efficiency. All local/nodal correlations to cognitive components are provided in Supplementary Tables 6–25.

### Local/nodal network properties predict cognitive performance in patients

A machine learning approach was used to determine how accurately local/nodal graph theory measures could predict working memory and reasoning performance in patients. When using measures of degree centrality from the working memory network, a significant model predicting working memory performance was observed (*r = *0.22, *P = *0.045; *R*^2^ = 0.19, *P < *0.01; MSE = 1.09, norm MSE = 1.09). Computation of weights indicated that the right pallidum, left superior frontal, right paracentral, right supramarginal and right insula nodes contributed most prominently to the model (Supplementary Fig. 6A). A significant model was also observed when predicting reasoning performance (*r = *0.22, *P = *0.044; *R*^2^ = 0.13, *P = *0.038; MSE = 1.02, norm MSE = 1.08). Regional weights indicated that degree centrality measures from the left caudal anterior cingulate, right paracentral, right precentral, right rostral anterior cingulate and right supramarginal nodes contributed most prominently to the model (Supplementary Fig. 6B). Network measures of local efficiency did not provide a significant correlation for the prediction of working memory (*r = *0.16, *P = *0.14; *R*^2^ = 0.15, *P = *0.026; MSE = 1.09, norm MSE = 1.09) but did for the prediction of reasoning (*r = *0.34, *P < *0.01; *R*^2^ = 0.19, *P < *0.01; MSE = 0.98, norm MSE = 0.99). Regional weights indicated the greatest contribution to the model from the right precentral, right supramarginal and right insula nodes. No significant predictive models were observed when using clustering coefficient measures (Supplementary Fig. 7).

Conversely, when examining the predictive value of local/nodal measures in the reasoning network, only significant models were observed for the prediction of reasoning performance. A significant model was observed using degree centrality to predict reasoning (*r = *0.38, *P < *0.01; *R*^2^ = 0.21, *P < *0.01; MSE = 0.98, norm MSE = 1) with computation of weights indicating the greatest contribution to the model by measures from the left lateral occipital, right cuneus, right inferior temporal pole, right lingual, right pericalcarine and right superior parietal nodes (Supplementary Fig. 8A). In contrast, no significant model was observed when predicting working memory (*r = *0.09, *P = *0.32; *R*^2^ = 0.10, *P = *0.33; MSE = 1.09, norm MSE = 1.10). A significant model was observed when using local efficiency measures to predict reasoning (*r = *0.30, *P < *0.01; *R*^2^ = 0.13, *P = *0.033; MSE = 0.99, norm MSE = 0.99) but not for the prediction of working memory performance (*r = *0.09, *P = *0.28; *R*^2^ = 0.05, *P = *0.51; MSE = 1.08, norm MSE = 1.09). Local efficiency of the left pars opercularis, left superior frontal and right rostral middle frontal nodes contributed the greatest to this model (Supplementary Fig. 8B). No significant models were observed when using clustering coefficient measures.

## Discussion

Our results provide evidence that working memory and reasoning deficits after TBI relate to dissociable patterns of white matter damage, as demonstrated by their differential relationships to functionally distinct structural networks. We demonstrate, for the first time, that TBI impacts on both working memory and reasoning using a large cohort of patients. A notable strength of our data-driven approach, is not only that it provides a further replication of the findings of Hampshire *et al.* (2012), by illustrating that working memory and reasoning form two distinct cognitive abilities; it demonstrates that TBI can impact on either one of these abilities when taking into account the other. This point is important because cognitive tasks tend to tap multiple abilities and the behavioural basis of deficits can therefore be hard to interpret if individual tasks are analysed in isolation. We also replicate the finding that disruption to network hubs has a particularly significant impact on the cognitive processes they support as per previous research (Fagerholm *et al.*, 2015). By intersecting an established atlas of white matter connections with our reasoning and working memory task-functional MRI activation maps, we confirmed that individual differences in the integrity of the information processing architecture within functionally-distinct subnetworks of the structural connectome underpin different axes of cognitive ability.

Broadly speaking, this accords with the prediction of Hampshire *et al.* (2012), where it was observed that tasks that activate the same functional networks also tend to load on to similar behavioural abilities when factor analysing intersubject variability in their behavioural scores. This interpretation was controversial when first proposed (Haier *et al.*, 2014) and our hypothesis sought to extend this by suggesting that damage to a functionally distinct subnetwork would impact on the associated axes of cognitive ability. Here our results confirm this hypothesis but also provide refinement in our understanding of the way in which functionally distinct structural subnetworks support reasoning and working memory abilities. More specifically, whilst the expected dissociation between reasoning and working memory structural networks was evident, the association to working memory and reasoning performance unexpectedly provided evidence of a hierarchal functional relationship between the two networks.

The analysis of behavioural data confirmed the hypothesis that working memory and reasoning are distinct aspects of human cognitive ability and that they can be differentially affected by neurological damage. Previous studies investigating cognitive impairment after TBI have shown that tasks that tap either of these aspects of cognition can be affected by TBI (McAllister *et al.*, 2004; Kinnunen *et al.*, 2011; Palacios *et al.*, 2011; De Simoni *et al.*, 2018). However, working memory and reasoning have seldom been investigated in parallel, limiting our understanding of the varied prevalence of impairments after TBI and the mechanisms contributing to them. In studies of healthy individuals, factor analyses of >44 600 members of the general public have demonstrated that working memory and reasoning form distinct axes of cognitive ability (Hampshire *et al.*, 2012; Daws and Hampshire, 2017). Here we observed relatively uniform deficits in the performance of all six cognitive tasks when comparing mean scores for TBI patients relative to controls. This indicated that they were all similarly susceptible to neurotrauma. Nonetheless, the PCA of the cognitive data replicated the two cognitive components observed in previous literature (Hampshire *et al.*, 2012; Daws and Hampshire, 2017) in an unbiased data-driven manner; specifically, tasks that involved maintaining information in working memory (MKL, PAL, SOS) loaded onto one principal component whereas tasks that involved transforming information according to rules (FTM, OOO, HTT) loaded onto another. This was the case when analysing all data together, or when analysing patient data alone.

Widespread diffuse axonal injury was also observed in TBI patients compared to controls in our voxelwise analysis, suggesting disruption to these networks may impact upon cognition, as per previous research (Fagerholm *et al.*, 2015). The impact of diffuse axonal injury on cognition may be best understood mechanistically from a graph theoretic perspective as the balance between network integration (global efficiency) and segregation (clustering coefficient/local efficiency) is described as key in the emergence of small-world organization of networks that support cognition (Bullmore and Sporns, 2009; Braun *et al.*, 2015; Cohen and D'esposito, 2016). Alterations to this balance reflect aberrant information processing properties that may lead to impaired cognition. Analysis of the global graph theoretic measures revealed abnormalities in both the working memory and reasoning networks of TBI patients compared to controls, remaining consistent across 80% of the alternative thresholds examined. We replicated the observation of disruption away from optimal small-world topology as illustrated by a reduction in global efficiency and increase in local efficiency, which has previously been shown in TBI (Pandit *et al.*, 2013). Evidence of reduced average degree in networks suggest that this move away from small-worldness may in part be due to loss of long-range white matter connections (Sharp *et al.*, 2014).

The results from our canonical correlation analysis further statistically supported our prior hypothesis that multiple latent variables or ‘modes’ relate working memory and reasoning abilities to measures of white matter tract integrity throughout the brain. An advantage of this method is that it identifies the number of latent variables that are common across different matrices of data in an unbiased, data-driven manner. Back-projection of the canonical variates onto the behavioural matrix reproduced the working memory versus reasoning dissociation. Back projection onto the node degree centrality matrix partially dissociated the nodes of the working memory and reasoning networks. These results provide further confirmation of the differential impact of diffuse axonal injury on working memory and reasoning in white matter previously associated to impairment after TBI (McAllister *et al.*, 2004; Kinnunen *et al.*, 2011; Palacios *et al.*, 2011; De Simoni *et al.*, 2018) and provide further support for our initial graph theory findings. However, the main limitations of CCA are that the models are, by design, overfitted. We directly tested this limitation using a custom subsampling permutation test and illustrated that our two modes differed from the null distribution of permuted data, indicating real dissociable relationships were observed. Nevertheless, CCA analyses do not provide mechanistic insights, for example, regarding the impact of observed individual differences on network information processing properties; therefore, they require other complementary forms of analysis to gauge the scale of relationship and interpret them mechanistically.

Critically, when cross-correlating behavioural components and graph theoretic measures for these subnetworks, we found similar scaled correlations between reasoning performance and global graph theoretic measures in the reasoning and working memory subnetworks, but significant correlations between working memory performance and the working memory subnetwork only. This one-way dissociation was even more pronounced in the analysis of node-centric graph theoretic measures. Patients had abnormalities in degree centrality, local efficiency and clustering coefficient for a large proportion of nodes within the working memory and reasoning networks, and this was robustly replicated across 80% of the alternative thresholds. When correlated to cognitive components, similar significant correlations to degree centrality, local efficiency and clustering coefficient were observed with consistently strong relationships in network hubs, supporting previous literature demonstrating the impact of hub disconnection on cognitive impairment (Fagerholm *et al.*, 2015). Importantly, cross-correlation revealed a distinct and strong relationship of reasoning performance to these metrics in both the working memory and reasoning subnetworks, but for working memory performance there were significant relationships to nodes within the working memory network only. This one-way dissociation was robust, being evident in 70% of the threshold iterations (i.e. excluding the most liberal and strict thresholds).

Although we had not predicted this hierarchy when planning the study, this dissociation is precisely what is predicted in the classic literature regarding hierarchal models of working memory (Petrides, 1989, 1994; D’Esposito *et al.*, 1999). More specifically, those models proposed that information must first be maintained in working memory before it can be processed according to task rules. In accordance with this, disruption to the working memory network correlates with impairments in both working memory and reasoning, whereas disruption of the reasoning network has a more selective correlation with reasoning score. From this perspective, it is interesting to note the relationship of reasoning and working memory abilities to distinct but partially overlapping combinations of brain regions. This overlap is unsurprising from a network science perspective, as the same nodes typically contribute to multiple networks, but in different combinations; indeed, this has been shown to be the case for the tasks applied here (Lorenz *et al.*, 2018; Soreq *et al.*, 2019). Here, three of the five reasoning hubs were also present as nodes within the working memory network, which provides clues regarding how these networks may operate cooperatively to support the processing of actively maintained information.

From a clinical perspective, the dissociation of working memory and reasoning impairments provides new insights into the systems-level basis for heterogeneity in the chronic cognitive problems that TBI patients suffer from. These impairments have a major impact on a patient’s quality of life and warrant the need for improved therapies to be developed. An exciting avenue of research has begun to attempt to treat these impairments via pharmacological interventions, neurostimulation [e.g. transcranial direct/alternating current stimulation (tDCS/tACS)] and cognitive rehabilitation; however, there remains much debate about the efficacy of these treatments in TBI with little to no standard framework that can be translated into real-world clinical practice. This is likely due to the need to tailor treatments dependent on the exact nature of impairments that the patients have. A connectomic description is likely to have value when predicting chronic cognitive problems or developing individually tailored therapies. A sensible way to achieve this is to consider concurrently the behavioural profile of impairment and the networks on the brain that are affected in the individual.

This neurocognitive profiling could also be used to subclassify the patients when validating pharmacotherapies, cognitive interventions, and experimental therapeutics such as neurostimulation (Violante *et al.*, 2017; Li *et al.*, 2019). For example, individuals who have deficits due to damage to functionally distinct parts of the structural connectome are likely to respond best to different treatments. Indeed, we have recently reported that the efficacy of dopaminergic therapy can be predicted based on the degree of damage to fronto-striatal circuits (Jenkins *et al.*, 2018). Furthermore, we have illustrated that the efficacy of modulating functional networks using tDCS is associated with the structural connectivity underlying the stimulation target (Li *et al.*, 2019). Here we provide a complementary way to subdivide the patients further according to the cortico-cortical networks that are affected and begin to test the clinical utility of this approach using machine learning regression. We demonstrate that local/nodal graph theoretic measures of our functional subnetworks can accurately predict working memory and reasoning. Furthermore, we demonstrate that prediction of working memory and reasoning within these networks demonstrates the same asymmetrical dissociation as our correlational analysis. Importantly, we begin to provide finer grained information regarding the relative contribution of nodes in the prediction of reasoning and working memory performance in our models that may help to inform future research regarding therapeutic targets and prognostic markers.

Nevertheless, there are several potential limitations that should be considered for future research. First, although our network measures could significantly predict cognitive performance in patients, future research would benefit from refinement of these measures for use as a clinical diagnostic measure. Specifically, we demonstrate the predictive value of our measures in a chronic TBI population; longitudinal changes in graph theoretical measures of the structural connectome have been shown to be associated with improved recovery (Kuceyeski *et al.*, 2019) and future research would therefore benefit from utilizing and refining our subnetwork graph theory measures to predict the evolution of reasoning and working memory impairments in an acute TBI population. Second, working memory and reasoning are themselves complex constructs (Hampshire *et al.*, 2012; Christophel *et al.*, 2017; Soreq *et al.*, 2019) that subdivide the networks examined here at a finer grain. In fact, we have recently developed a paradigm that could be used for this diagnostic purpose because it strongly differentiates multiple aspects of working memory based on network connectomics with high reliability (Soreq *et al.*, 2019). Third, a broader characterization of the types of deficits that are relevant to TBI is required. Indeed the comparison of standard neuropsychological tests to our computerized measures has some relevance to this issue. Although substantial variance was shared between the two assessment approaches, the internal structure of that variance clearly differed. Specifically, standard neuropsychological assessments provided further factors that were affected by TBI that our tasks did not measure. This calls for a broader characterization of the distinct behavioural abilities that are most commonly affected in TBI and how they map to different patterns of damage within the structural connectome. Achieving this is a focus of our current research. Finally, in our current work we have not taken into account the presence of focal lesions that may also impact upon network functionality and network properties, which would be advantageous to explore further. Nevertheless, nodes within our networks do not overlap with lesion overlap maps constructed for this patient cohort which fits with a broader view that impairments primarily relate to dysconnectivity as opposed to damage localized within the grey matter (Gaffan, 2005).

In summary, TBI has dissociable effects on working memory and reasoning due to the differential degradation of information processing properties of the structural networks that subserve these functions. Our work provides new mechanistic insights by illustrating that the nature of this dissociation is one-sided, which accords with multifactorial accounts of human cognitive ability (Hampshire *et al.*, 2012) and provides clear evidence in support of hierarchical accounts of reasoning systems (D’Espoito and Postle, 2015). Our work provides potential markers for the identification of patients likely to develop working memory and reasoning impairments by demonstrating their predictive power using machine learning in a chronic TBI population. Further investigation using these measures in the prediction of long-term outcomes of acute patients may prove beneficial. Identifying the properties of aberrant networks and their relationship to working memory and reasoning impairments provides opportunities for individually targeted therapeutic interventions to be explored.

## Supplementary Material

awaa067_Supplementary_Data

## Abbreviations

CCA =: canonical correlation analysis
FTM =: Feature Match test
HTT =: Hampshire Tree test
MKL =: Monkey Ladder test
OOO =: Odd One Out test
PAL =: Paired Associative Learning test
PCA =: principal component analysis
SOS =: Self-Ordered Search
TBI =: traumatic brain injury
