# Supplementary material for: Distinct patterns of structural damage underlie working memory and reasoning deficits after traumatic brain injury
Source: Brain. 2020 Apr 3;143(4):1158–76. doi: 10.1093/brain/awaa067 (PMC7174032; doi:10.1093/brain/awaa067)
Supplement: awaa067_Supplementary_Data [file awaa067_supplementary_data.docx]

**Supplementary material**

**Supplementary information**

**Description of computerised cognitive tasks**

In the MKL, squares containing numbers are displayed on screen in random locations on an invisible 5x5 grid. After a short period of time, the numbers disappear but the squares remain. Individuals are required to remember the location of the numbers and respond by pressing in ascending numerical order on the blank squares where the numbers were. If the participant answers correctly, the next trial has one more square. If they answer incorrectly, then there is one less square. The task starts at a difficulty level of 2 squares. The main outcome measure is the average number of squares achieved. Mean population performance is 7.85, 1.154 S.D.

In the PAL task, boxes are displayed at random locations on the screen in an invisible 5x5 grid and reveal one at a time an image that is within them. Once all images have been revealed and subsequently hidden, an object that matches one of the concealed objects is displayed. Individuals must select which box the matching object is hidden in. If the participant answers correctly, the next trial has one more square. If they answer incorrectly, then there is one less square. The task starts at a difficulty level of 2 squares. The main outcome measure is average number of squares achieved. Mean population performance is 5.28, 1.13 S.D.

In the SOS task, boxes are displayed on the screen at random locations in an invisible 5x5 grid. Individuals need to search through boxes by clicking on them to collect hidden tokens. A token will not appear in the same box twice on a trial and the participant must continue to search through the boxes until all tokens have been found in every box. If an individual searches a box that has previously revealed a token or searches the same empty box before finding a token, they will incur an error. If the participant answers correctly, the next trial has one more square. If they answer incorrectly, then there is one less square. The task starts at a difficulty level of 4 boxes (minimum task level=2 boxes). The main outcome measure is the average number of boxes achieved. Mean population performance is 8.23, 2.1 S.D.

In FTM, two grids are displayed in parallel each containing a set of shapes. In half of the trials, the grids differ by one shape. The participant must decide whether the two grids are identical or different by selecting match or mismatch response button. Participants must solve as many problems as possible in 90 seconds. If the response is correct, the total score increases by the number of shapes prese4nted in the grid and the number of shapes presented in subsequent trials increases. If a participant makes an error the total score decreases by the number of shapes in the grid and the subsequent trials have fewer shapes than the previous trial. The first trial of grids contains on two shapes each. The main outcome measure is the total score achieved after 90 seconds. Mean population performance is 131.35, 32.79 S.D.

In OOO, a 3x3 grid of objects is displayed. The objects displayed contain a variable number of copies of a coloured shape. The features that make up the object in each cell of the grid (colour, shape, number of copies) are related to each other based on a set of rules. The participant must identify the rules that relate the object features and select the one cell whose contents do not correspond to those rules. The participant must solve as many problems as possible in 180 seconds. If the response is correct, the total score increases by one point and the next problem is more complex. If the response is incorrect, the total score decreases by 1 point. The outcome measure is the total score achieved in 180 seconds. Population mean=10.43, 3.31 S.D.

In HTT, a tree shaped structure containing numbered beads is displayed. Participants must rearrange the beads so that they sit in ascending numerical order on the tree running from left to right and top to bottom. The beads are highlighted as red when in an incorrect position and are green when located in the correct position on the tree. Participants must solve as many problems as possible in as few moves as possible within 90 seconds. Problems become progressively more difficult with the total number of moves required and complexity of planning increasing in each trial. Trials are aborted if the participant makes more than twice the number of moves required to solve the problem. After each trial, the score is increased by adding the minimum number of moves required multiplied by 2, minus the number of moves actually made. This allows for efficient planning to be rewarded. The first trial can be solved in 3 moves. The outcome measure is the total score achieved after 90 seconds. Population mean is 32, 10.19 S.D.

**Graph theory metrics**

Global efficiency (E_glob_) provides a measure of information flow or overall network efficiency and is defined as the inverse of all shortest path lengths in a given network (Latora & Machiori, 2001; Chen *et al*., 2013). In the context of white matter, global efficiency is associated with long-range connections that enable the rapid transfer of information across distal regions. Local efficiency (E_loc_) provides a measurement of information flow at the individual nodal level (Onnela *et al*., 2005, Klados *et al*., 2013) and is associated with short-range connections between nearby regions that enable modularity of information processing or ‘fault-tolerance’ in a network (Latora & Macrchiori, 2001). Degree centrality refers to the number of neighbours connected to a given node where Cij represents a connection between node I and node J. If Cij=0 there would be no connection whereas Cij>0 would represent a connection (Sporns, 2004; Gottlich *et al*., 2013). Finally, the clustering coefficient provides a measure of the extent to which nodes tend to group or cluster together. The clustering coefficient is defined as the number of ‘triangles’ on the nodal level (Watts & Strogatz., 1998, Onnela *et al*., 2005) or the proportion of connections among the neighbours of a node. Refer to Fagerholm *et al*., 2015 for detailed information of each graph theory metric.

**Description of canonical correlation analysis method**

Canonical correlation analysis (CCA) is a multivariate statistical method that allows for the measurement of the linear relationship between two multidimensional datasets. This method can be thought of as an extension of multiple linear regression in the sense that rather than having multiple independent variables and a single dependent variable, CCA uses multiple independent and dependent variables. In CCA, the linear relationships between a set of variables (X) on one side and a set of variables (Y) on the other side are explored. This technique seeks to find several linear combinations of the X variables and the same number of linear combinations of the Y variables in a way that best describes the correlations between the two datasets. These linear composites are referred to as canonical variates (V and U), with the correlations between the corresponding pairs of canonical variates being referred to as the canonical correlation (r). The number of canonical correlations (also known as canonical modes) produced from a CCA will be related to the smallest number of variables from either X or Y, thus if a CCA is performed between dataset X with two variables and dataset Y with five variables, two canonical modes will be produced. If more than one significant mode is observed, more than one linear relationship is observed between the two datasets that are dissociable from one another.

**Supplementary figures**

**
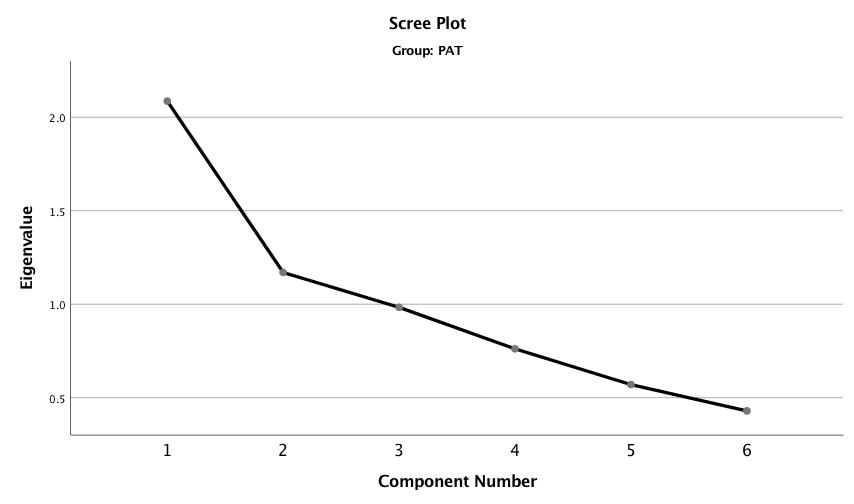
**

| **Task** | **MKL** | **PAL** | **SOS** | **FTM** | **OOO** | **HTT** |
| --- | --- | --- | --- | --- | --- | --- |
| **MKL** | 1 | 0.371 | 0.306 | 0.17 | 0.161 | 0.224 |
| **PAL** | 0.371 | 1 | 0.251 | 0.119 | 0.267 | 0.047 |
| **SOS** | 0.306 | 0.251 | 1 | 0.134 | 0.178 | 0.061 |
| **FTM** | 0.17 | 0.119 | 0.134 | 1 | 0.52 | 0.262 |
| **OOO** | 0.161 | 0.267 | 0.178 | 0.52 | 1 | 0.096 |
| **HTT** | 0.224 | 0.047 | 0.061 | 0.262 | 0.096 | 1 |

**Supplementary figure 1:** Scree plot and cross-correlation table of patient cognitive components from PCA analysis with orthogonal rotation.


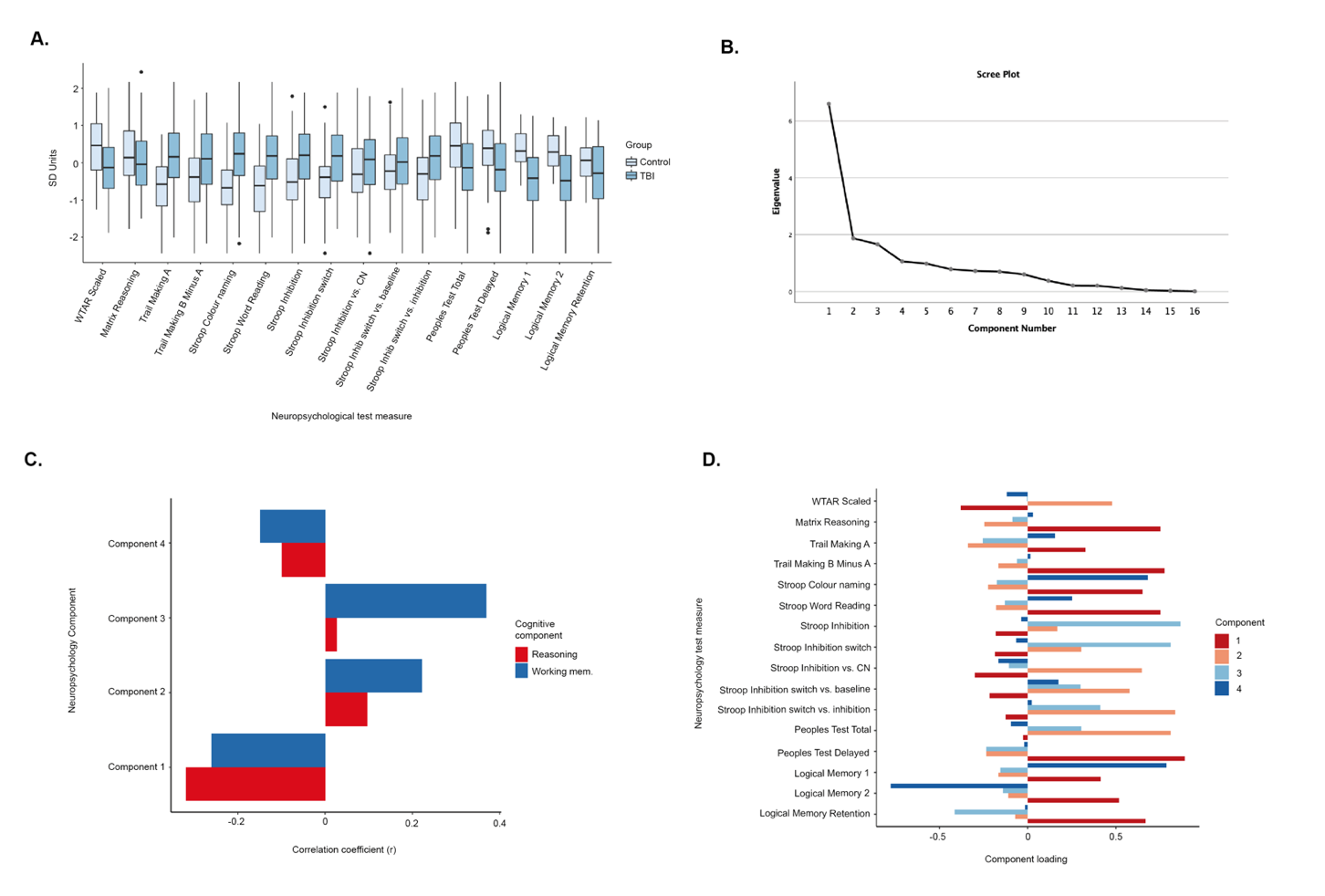


**Supplementary figure 2: Association between working memory/reasoning performance derived from computerised tasks and classical neuropsychological test performance in patients.** A. Performance as quantified in SD units on 16 sub-tests of the standard neuropsychological test battery previously used to assess TBI patients (Kinnunen *et al*., 2011; De Simoni *et al*., 2017). B. Component eigenvalues derived from principal component analysis (PCA) with orthogonal rotation of standard neuropsychological test performance in TBI patients. C. Bivariate correlations between working memory and reasoning performance derived from PCA of computerised task data with the four cognitive components derived from PCA of standard neuropsychological tests in the same patient cohort. D. Component loadings of each individual neuropsychological sub-test.


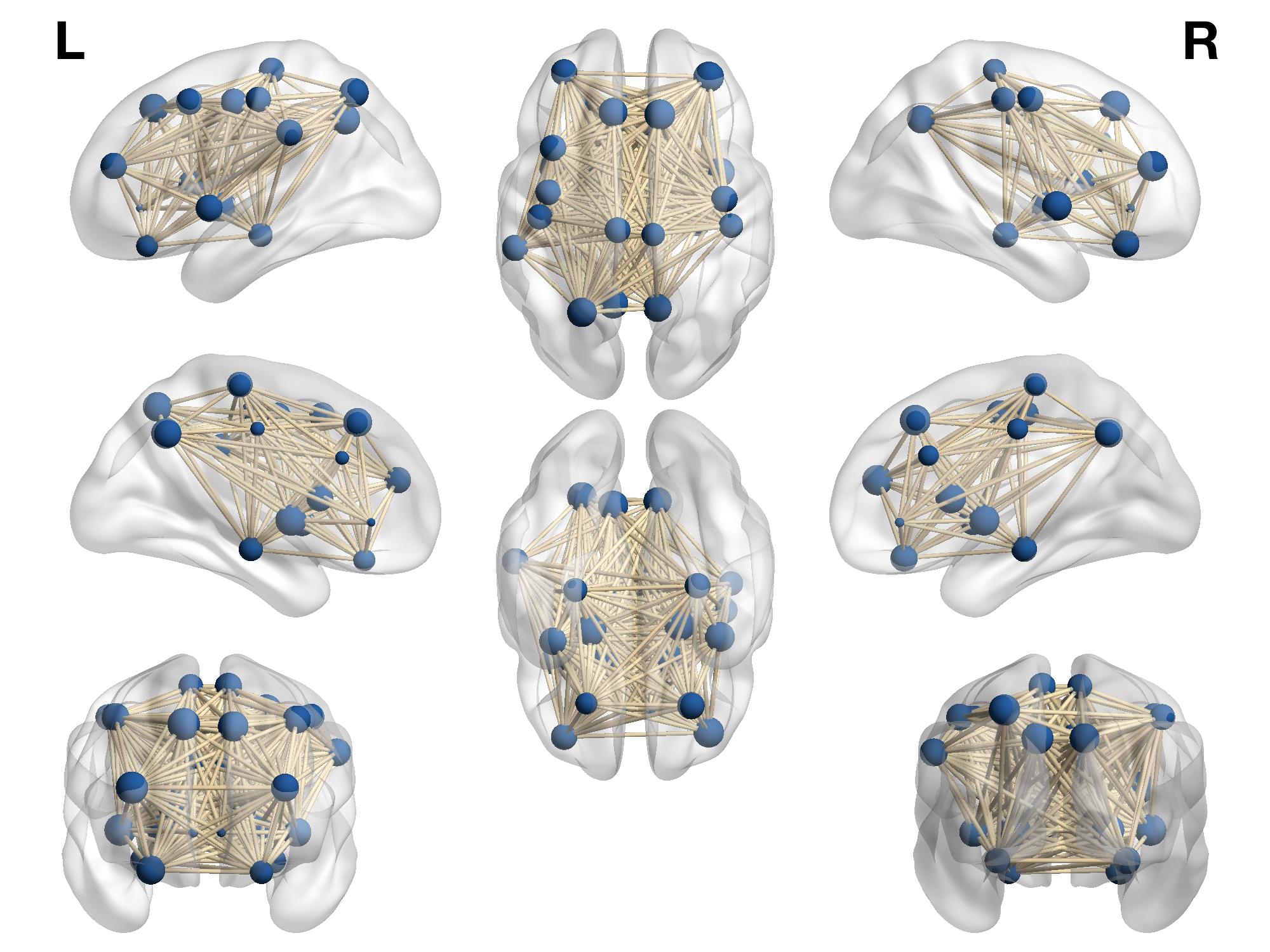

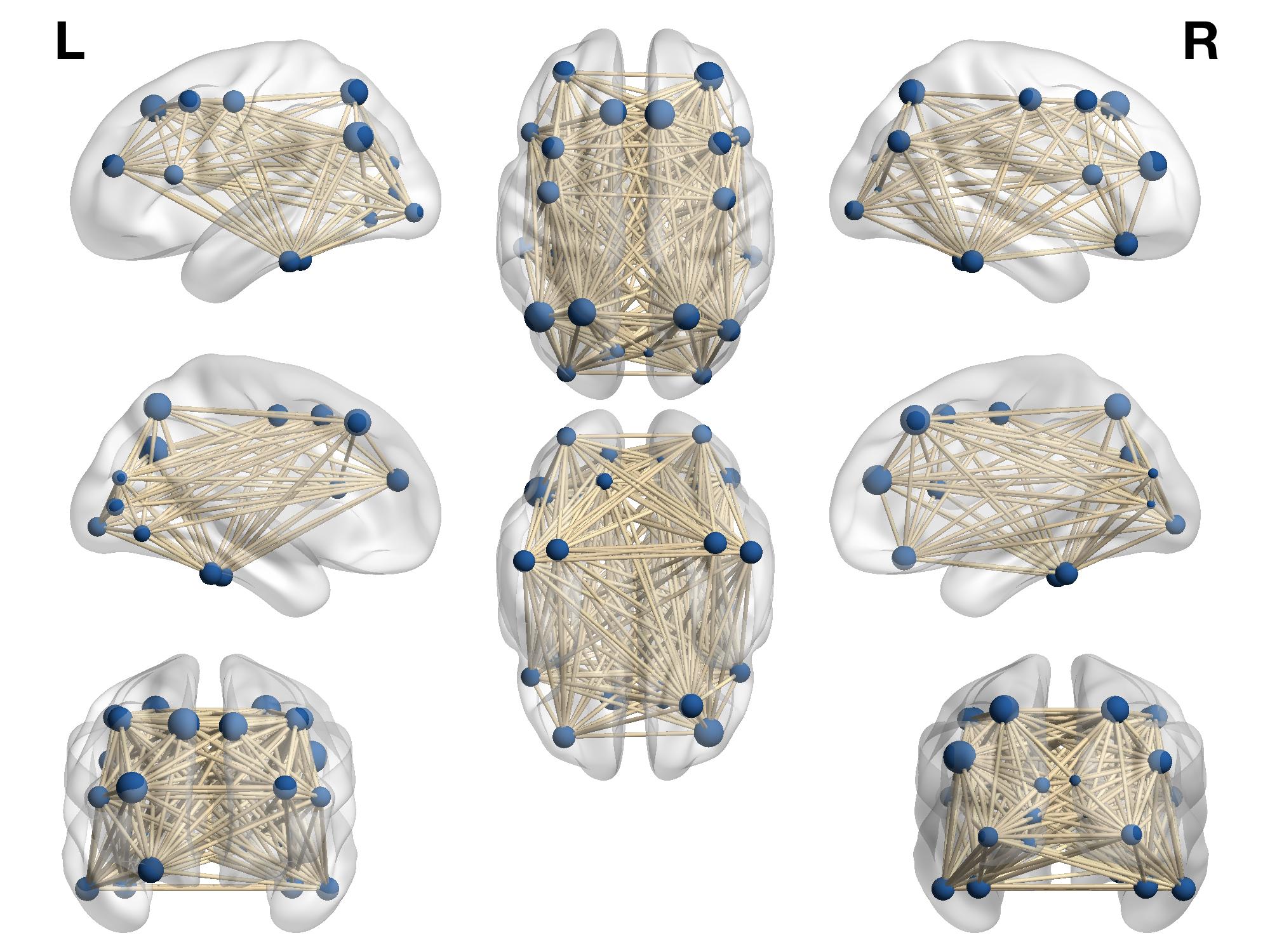


**Supplementary figure 3: Working memory (left) and reasoning networks (right) constructed using activation maps from Hampshire *et al*., 2012.** Node size reflects degree centrality, edge size reflects FA and no. streamlines for central threshold (2.5 SD).


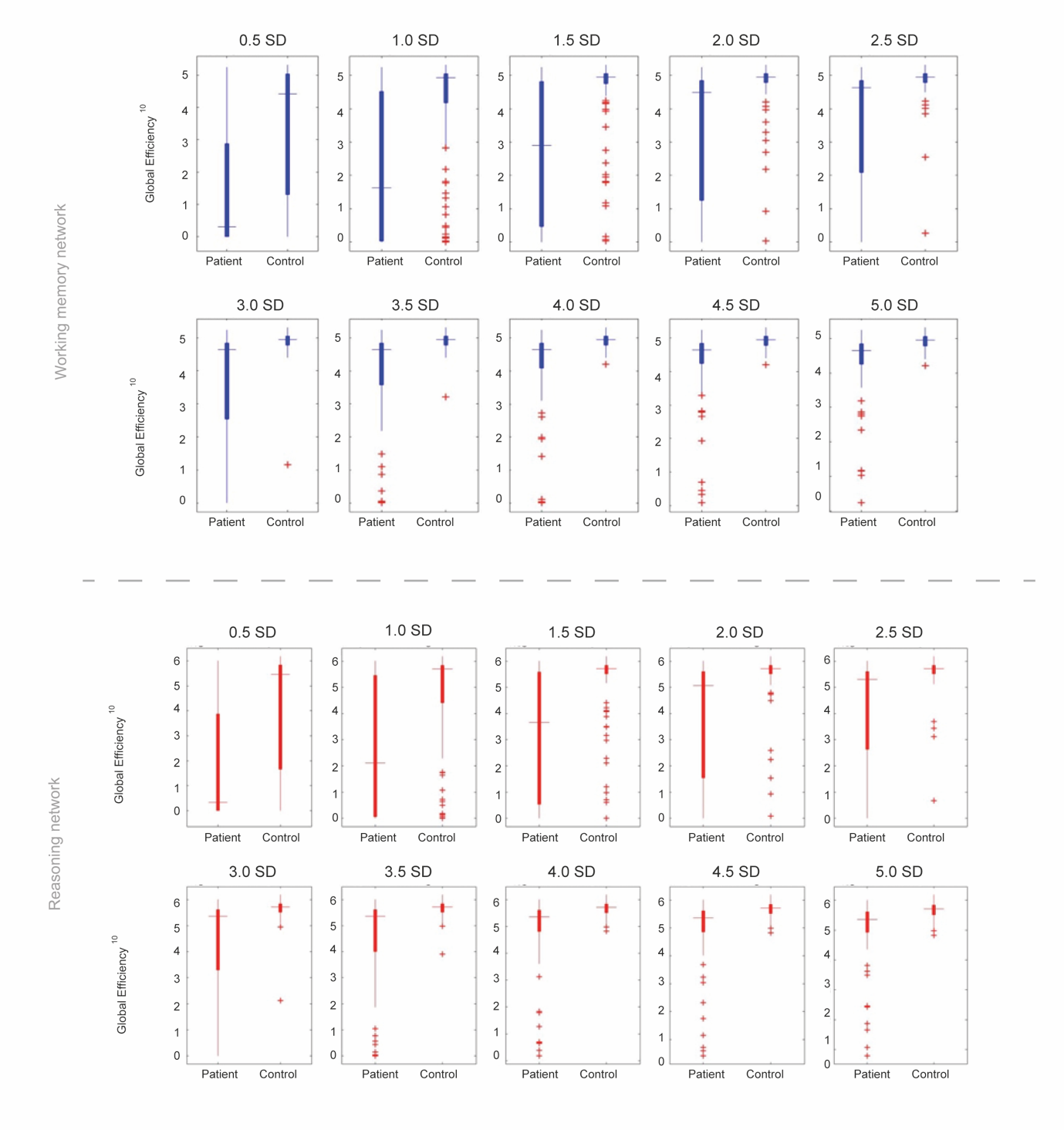


**Supplementary figure 4: Results from global cross sectional analysis of working memory and reasoning network graph theory properties across threshold iterations.** Blue figures represent working memory global network properties, red figures represent reasoning global network properties. Threshold iterations from 0.5-5 SD in 10 increments of 0.5 from left to right, top to bottom.


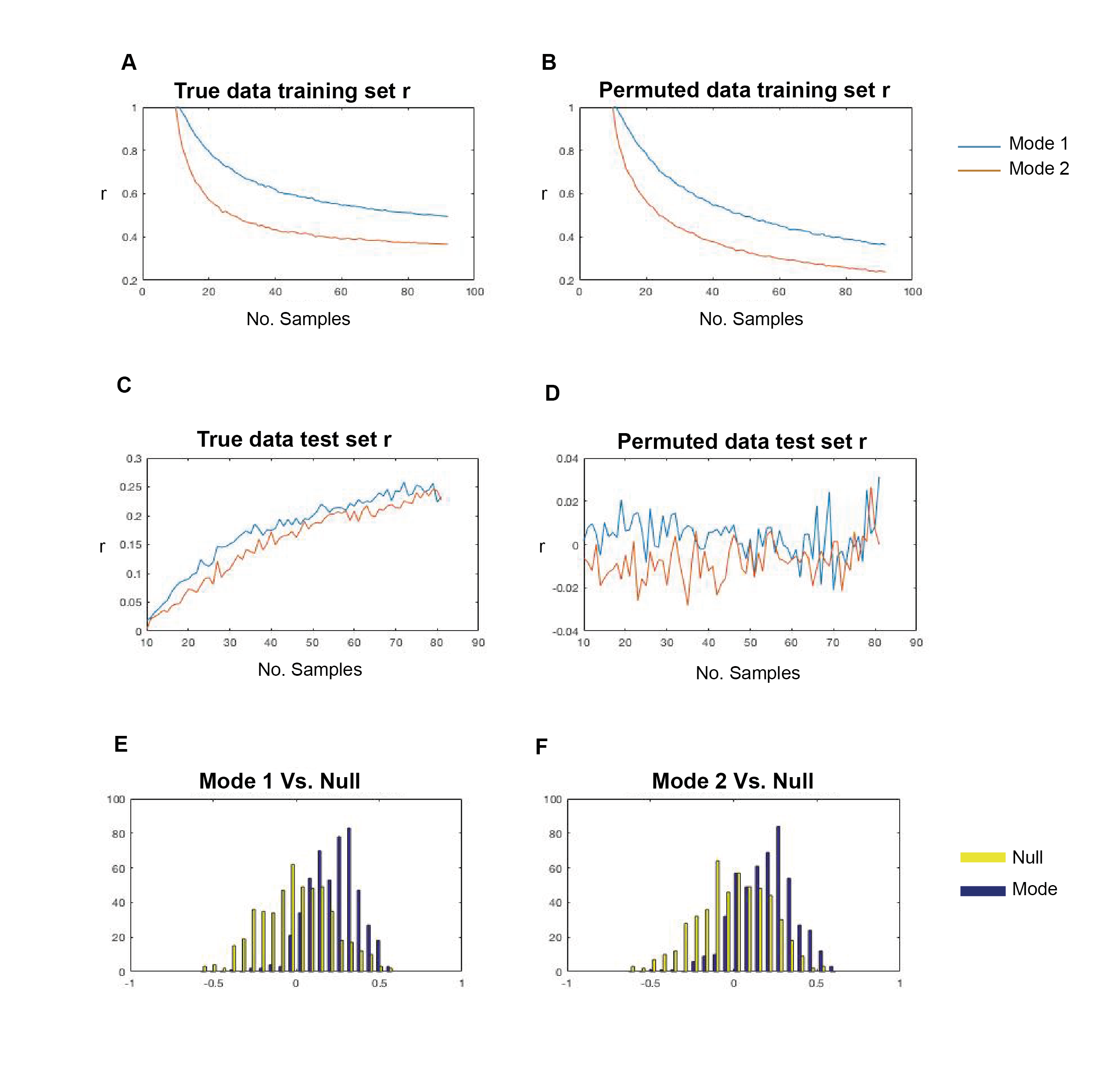


**Supplementary figure 5: Sub-sampling canonical correlation analysis results to assess model genralisability and overfit.** In order to quantify generalisability the CCA analyses were repeated in an iterative loop, whereby the data were randomly sub-sampled, the model trained, then applied to the held out data to which it was naiive. This process was repeated with systematic variation in the trained (N. samples) and held out (remaining samples) ratio, with r values averaged over 1000 iterations at each point. The process was then repeated using permuted data to assess overfit. **A.** Canonical r values for each mode calculated within the training set at varying sub-sample sizes. **B.** Canonical r values for each mode as calculated using the same training set data but with the labels permuted to break the behavioural-graph thery metric linkage. Note that although the r values are higher for the true data, they also are in the medium range for the permuted data. **C.** Canonical r values for the held out data, to which the model was naiive. **D.** Canonical r values calculated the same way as for ‘C’ but with the permuted data. Note that generalisation for the true data is in the r=0.25 range for both modes in the true data, but on average is close to 0 for the permuted data **E.** Distribution of canonical r values for Mode 1 using 1000 sub-samples at a 2:1 train/test ratio. Blue=true data, yellow = permuted data. **F.** The distribution of true and permuted canonical r values for Mode 2 for the same models as ‘E’.


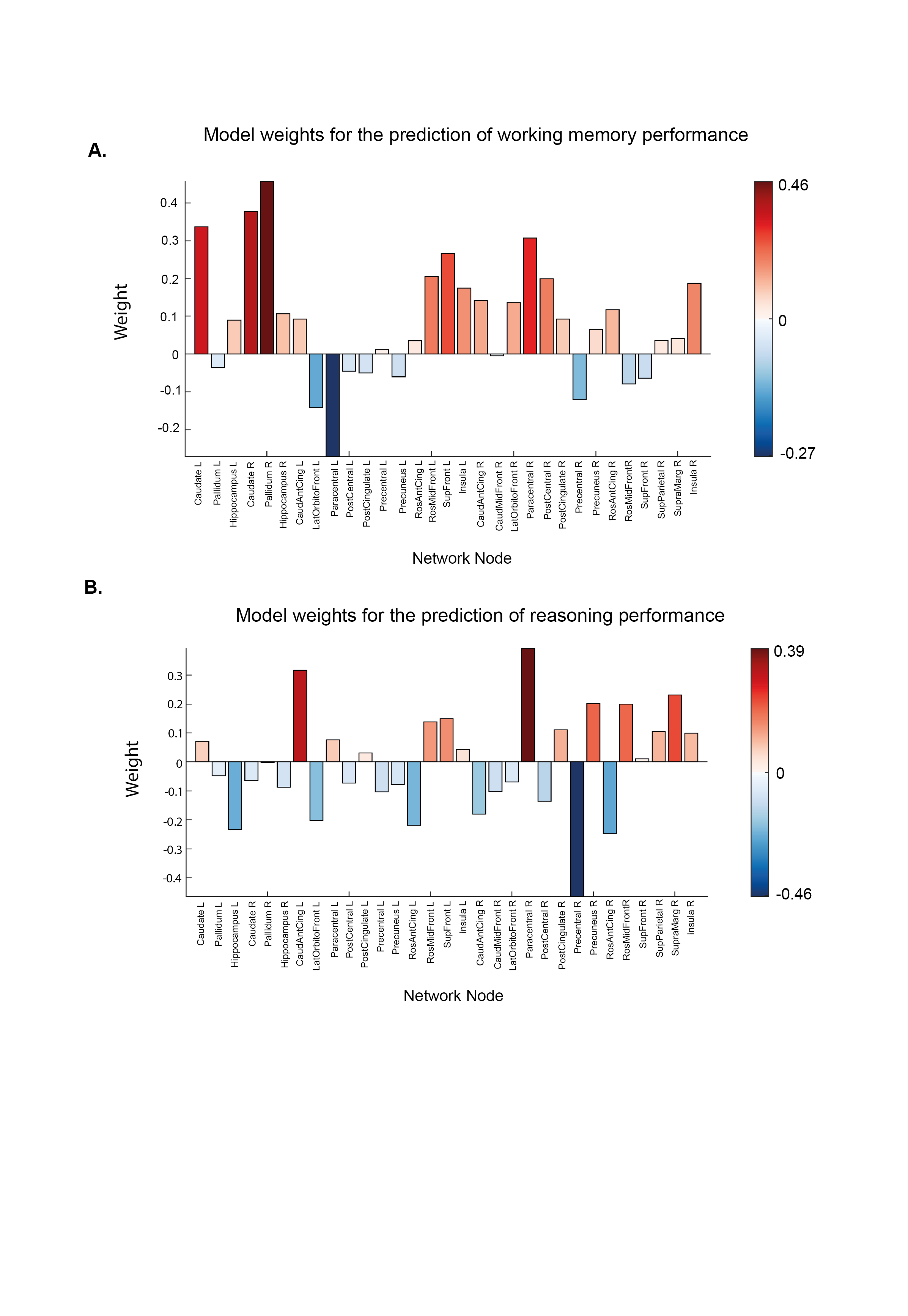


**Supplementary figure 6: Weights derived from PRoNTo machine learning regression models using local/nodal degree centrality measures of the working memory network.** A. Nodal weights derived from signfiicant kernel ridge regression (KRR) model predicting working memory performance in patients using degree centrality measures. B. Nodal weights derived from signfiicant KRR model predicting reasoning performance in patients using degree centrality measures. Greater weight value in either direction reflects greatest contribution to predictive model.


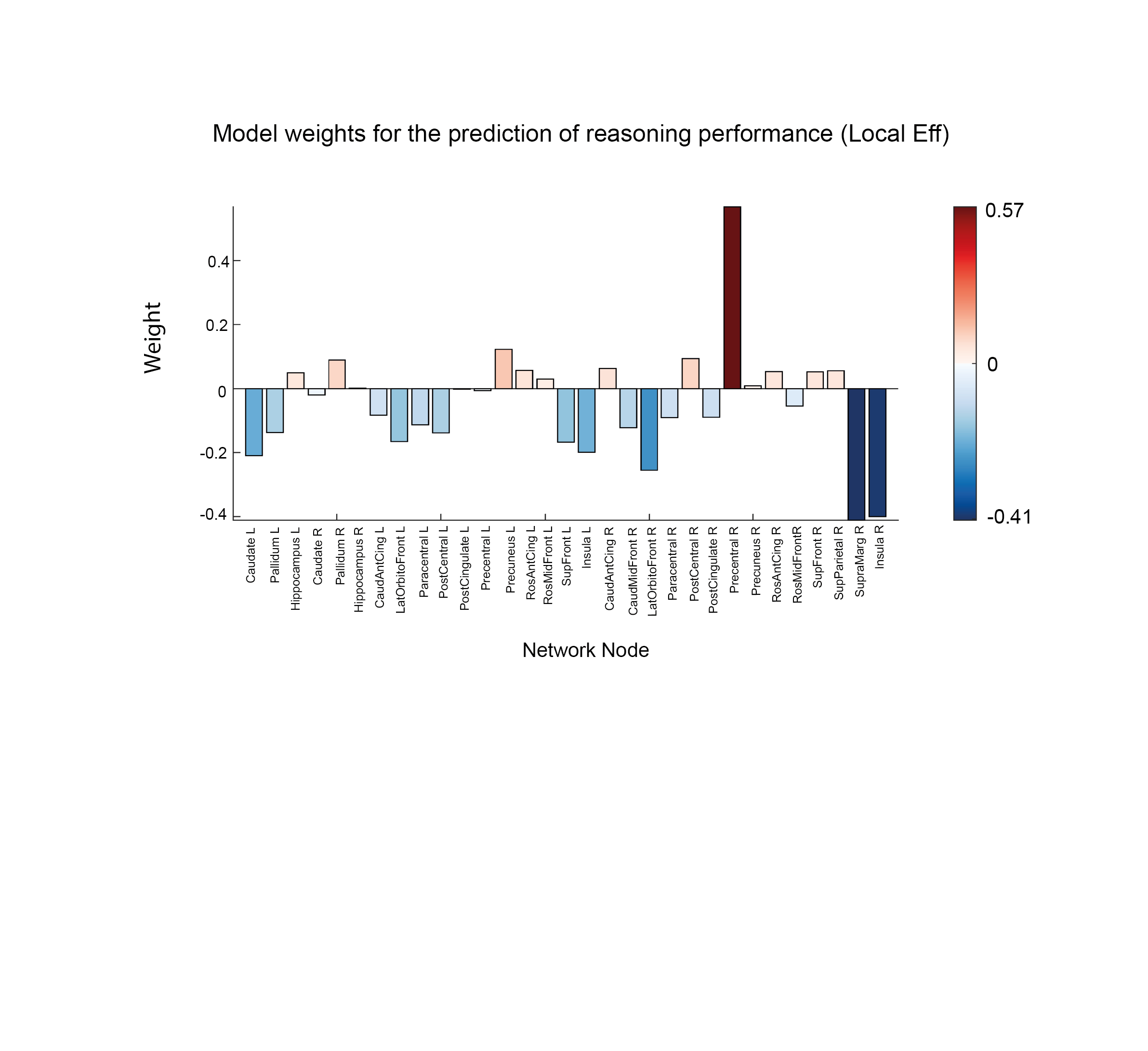


**Supplementary figure 7: Weights derived from PRoNTo machine learning regression models using local/nodal local efficiency measures of the working memory network to predict reasoning performance.** Greater weight value in either direction reflects greatest contribution to predictive model. Local eff=local efficiency.


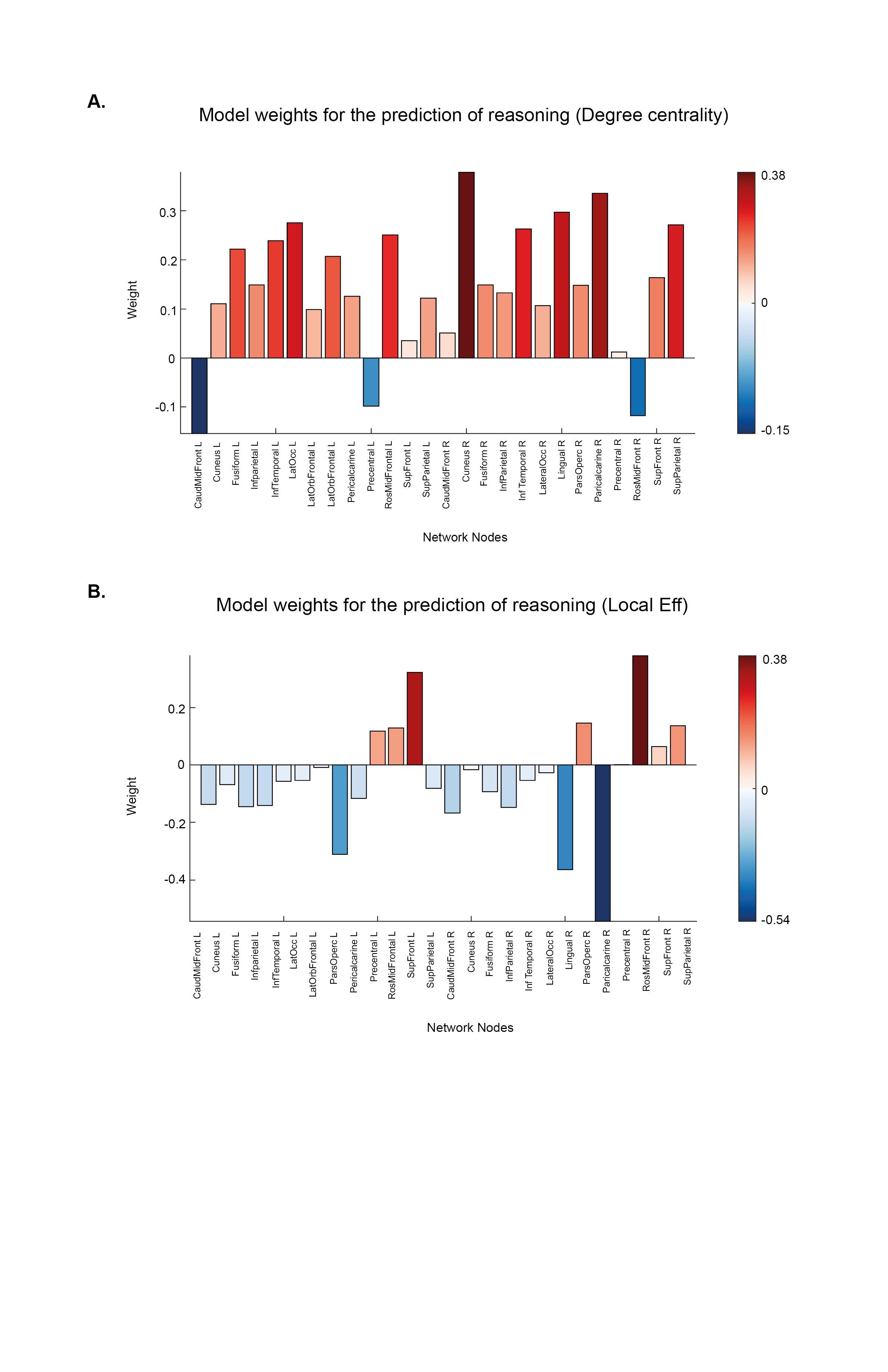


**Supplementary figure 8. Weights derived from PRoNTo machine learning regression models using local/nodal degree centrality measures of the reasoning network.** A. Nodal weights derived from significant KRR model predicting reasoning performance in patients using degree centrality measures. B. Nodal weights derived from significant KRR model predicting reasoning performance in patients using local efficiency measures. Greater weight value in either direction reflects greatest contribution to predictive model.

**
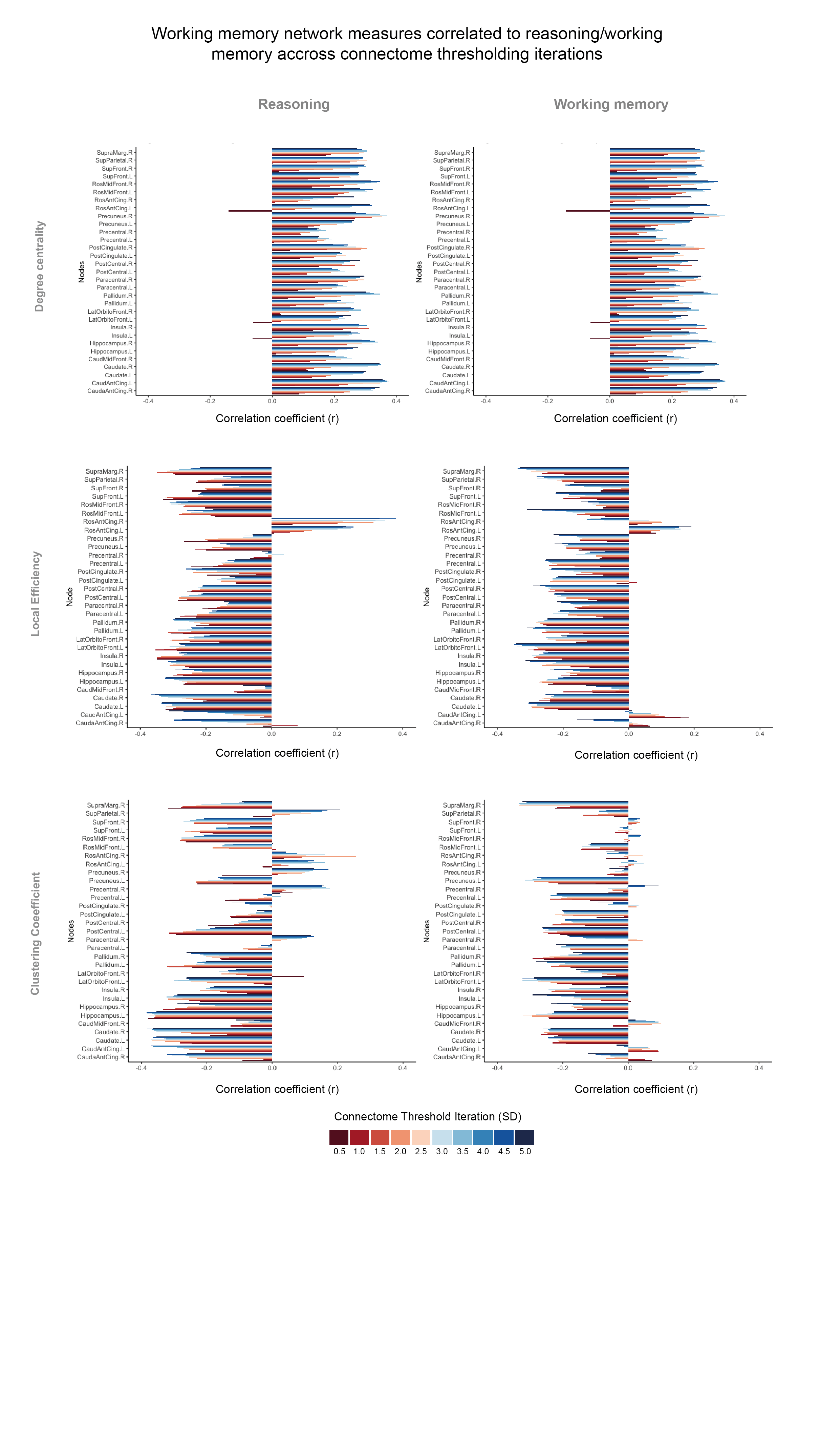
**

**Supplementary figure 9: Correlation coefficients of local/nodal working memory network measures across the ten connectome threshold iterations.** Plots on left illustrate correlation coefficients for working memory network node measures (degree centrality, local efficiency and clustering coefficient) to reasoning performance across all ten connectome threshold iterations. Plots on right illustrate correlation coefficients for working memory network node measures (degree centrality, local efficiency and clustering coefficient) to working memory performance across all ten connectome threshold iterations. Connectome threshold iterations refer to multiplying the control SD connectivity matrix by the threshold value (e.g. 0.5, 1.0, 1.5, 2.0, 2.5, 3.0, 3.5, 4.0, 4.5 and 5.0 SD) and subtracting this from the control mean connectivity matrix. The resulting threshold values derived from this calculation were then applied to each patient and control’s individual connectivity matrix such that any value less than the threshold value calculated for a connection was replaced with a zero.

**
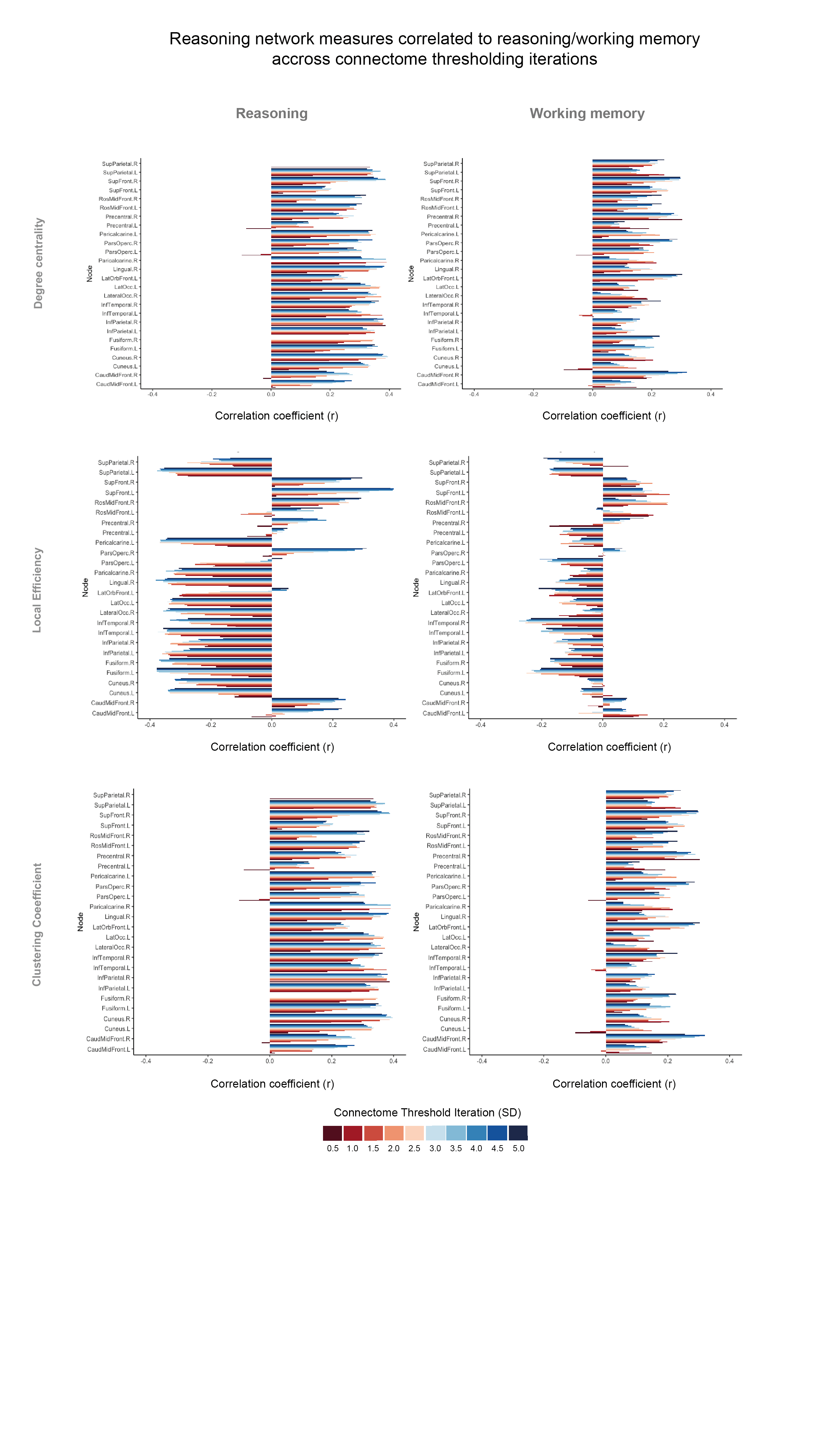
**

**Supplementary figure 10: Correlation coefficients of local/nodal reasoning network measures across all ten connectome threshold iterations.** Plots on left illustrate correlation coefficients for reasoning network node measures (degree centrality, local efficiency and clustering coefficient) to reasoning performance across all ten connectome threshold iterations. Plots on right illustrate correlation coefficients for reasoning network node measures (degree centrality, local efficiency and clustering coefficient) to working memory performance across all ten connectome threshold iterations. Connectome threshold iterations refer to multiplying the control SD connectivity matrix by the threshold value (e.g. 0.5, 1.0, 1.5, 2.0, 2.5, 3.0, 3.5, 4.0, 4.5 and 5.0 SD) and subtracting this from the control mean connectivity matrix. The resulting threshold values derived from this calculation were then applied to each patient and control’s individual connectivity matrix such that any value less than the threshold value calculated for a connection was replaced with a zero.

**Supplementary Tables**

| Age | Gender | Severity | Cause | PTA | Contusion | Microbleeds | Medication |
| --- | --- | --- | --- | --- | --- | --- | --- |
| 65 | M | Mod/Sev | RTA | >7 days | Y | N | nil |
| 38 | M | Mod/Sev | violence/assault | 2 days | N | Y | nil |
| 51 | F | Mod/Sev | incident fall | 2 days | unknown | unknown | Lansoprazole 15mg od |
| 31 | M | Mod/Sev | RTA | 90 days | Y | Y | nil |
| 54 | M | Mod/Sev | RTA | 5 days | N | N | Citalopram 30mg, thyroxine 150 mcg |
| 20 | M | Mod/Sev | incident fall | 28 days | N | Y | nil |
| 36 | M | Mod/Sev | violence/assault | 3 days | Y | N | nil |
| 44 | M | Mod/Sev | RTA | 90 days | Y | Y | nil |
| 51 | M | Mod/Sev | RTA | several weeks | Y | Y | nil |
| 64 | M | Mod/Sev | RTA | few hours to a day | Y | N | Omeprazole 40mg, Levothyroxine 75mcg |
| 58 | M | Mod/Sev | RTA | 1 month | Y | N | nil |
| 34 | M | Mod/Sev | other | 6 weeks | Y | Y | nil |
| 45 | M | Mod/Sev | RTA | 2 weeks | N | Y | nil |
| 20 | M | Mod/Sev | RTA | 2 months | Y | Y | nil |
| 52 | M | Mod/Sev | RTA | 5 days | N | Y | nil |
| 45 | M | Mod/Sev | other | 14 days | N | N | nil |
| 31 | M | Mod/Sev | violence/assault | months | Y | Y | nil |
| 49 | M | Mod/Sev | other | yes, but unknown duration | Y | Y | Loperamine 2mg |
| 24 | M | Mod/Sev | RTA | 14 days | N | N | nil |
| 52 | M | Mod/Sev | incident fall | 21 days | Y | Y | Levetiracetam 1250mg bd, perindopril 2mg od |
| 54 | M | Mod/Sev | violence/assault | 15 days | Y | Y | nil |
| 26 | M | Mod/Sev | violence/assault | 56 days | Y | N | Amitriptyline 10mg, Sodium Valproate 100mg |
| 21 | F | Mod/Sev | RTA | 35 days | N | Y | Fluoxetine 20mg |
| 39 | F | Mod/Sev | incident fall | 2 days | N | N | nil |
| 47 | M | Mod/Sev | RTA | 3 days | Y | Y | nil |
| 22 | M | Mod/Sev | RTA | 450 days | Y | Y | nil |
| 34 | M | Mod/Sev | incident fall | 0 days | Y | N | nil |
| 48 | M | Mod/Sev | RTA | 60 days | Y | N | Amitriptyline 20mg tds, Fexofenadine 180mg od,  Esomeprazole 40mg od  Solifenacin 5mg od  Loperamine 10mg od |
| 38 | M | Mod/Sev | violence/assault | 700 days | Y | N | nil |
| 49 | F | Mod/Sev | violence/assault | 10 days | Y | N | Levothyroxine 150mcg od, omeprazole |
| 36 | F | Mod/Sev | RTA | 120 days | Y | Y | nil |
| 43 | M | Mod/Sev | RTA | 42 days | N | Y | nil |
| 31 | M | Mod/Sev | violence/assault | 21 days | Y | N | Growth Hormone 2mg, Lamotrigine 50mg bd |
| 37 | M | Mod/Sev | incident fall | 120 days | Y | N | Tegretol CR 400mg BD |
| 33 | M | Mod/Sev | RTA | 30 days | Y | Y | Citalopram 10mg OD, Baclofen 20mg OD |
| 35 | M | Mod/Sev | incident fall | 7 days | Y | Y | nil |
| 33 | M | Mod/Sev | RTA | 28 days | Y | N | Symbicort |
| 52 | M | Mod/Sev | violence/assault | 4 days | Y | N | Lansoprazole 15mg od |
| 38 | M | Mod/Sev | violence/assault | 14 days | unknown | unknown | nil |
| 31 | M | Mod/Sev | RTA | 120 days | unknown | unknown | nil |
| 39 | M | Mod/Sev | RTA | 2 weeks | Y | N | nil |
| 24 | F | Mod/Sev | RTA | 120 days | unknown | unknown | Citalopram 40mg OD |
| 35 | M | Mod/Sev | incident fall | 3 days | unknown | unknown | Salbutamol, Beclomethasone |
| 42 | M | Mod/Sev | RTA | unknown | N | N | Modafinil 300od, citalopram 20 OD, |
| 37 | M | Mod/Sev | RTA | 4 days | Y | N | Omeprazole 40mg OD, Gabapentin 900mg OD |
| 6 | M | Mod/Sev | incident fall | 4-6 hours | N | N | nil |
| 59 | M | Mod/Sev | RTA | 7 days | N | Y | nil |
| 57 | F | Mod/Sev | incident fall | 1-7 days | Y | Y | Demeclocycline 300g bd, ramipril 5mg od,  salbutamol brn, |
| 54 | M | Mod/Sev | incident fall | 2 days | Y | N | nil |
| 39 | M | Mod/Sev | RTA | unknown | N | Y | nil |
| 44 | M | Mod/Sev | RTA | 6 days | Y | Y | unknown |
| 48 | M | Mod/Sev | violence/assault | 24-48 hours | N | N | Carbamazepine 200mg |
| 65 | M | Mod/Sev | unknown | unknown | Y | Y | unknown |
| 59 | M | Mod/Sev | RTA | 1-7 days | Y | N | Lansoprazole 30mg od |
| 37 | M | Mod/Sev | incident fall | 1 days | Y | N | nil |
| 26 | M | Mod/Sev | RTA | 3 days | Y | Y | nil |
| 24 | M | Mod/Sev | RTA | unknown | Y | N | nil |
| 39 | M | Mod/Sev | violence/assault | 3 weeks | Y | N | nil |
| 53 | M | Mod/Sev | incident fall | unknown | Y | Y | nil |
| 29 | F | Mod/Sev | RTA | still in PTA | Y | Y | Lansoprazole 30mg, Phenytoin 300mg, |
| 51 | M | Mod/Sev | unknown | unknown | Y | Y | Gabapentin 300mg tds, Amitriptyline 50 mg |
| 52 | M | Mod/Sev | Incident fall | 24-48 hours | Y | Y | nil |
| 57 | M | Mod/Sev | RTA | 4 days | Y | N | unknown |
| 40 | M | Mod/Sev | RTA | 42 days | Y | N | nil |
| 42 | F | Mod/Sev | RTA | 3 days | Y | Y | nil |
| 40 | F | Mod/Sev | unknown | unknown | Y | N | unknown |
| 31 | M | Mod/Sev | incident fall | unknown | N | Y | nil |
| 34 | M | Mod/Sev | unknown | 3 days | Y | N | nil |
| 49 | F | Mod/Sev | incident fall | 24-48 hours | Y | N | Ibuprofen |
| 39 | M | Mod/Sev | unknown | 7 days | Y | N | ramipril |
| 44 | F | Mod/Sev | RTA | unknown | Y | N | unknown |
| 49 | M | Mod/Sev | RTA | unknown | unknown | unknown | nil |
| 61 | F | Mod/Sev | RTA | 120 days | Y | Y | Citalopram 10mg od |
| 39 | F | Mod/Sev | RTA | unknown | Y | Y | citalopram 20mg OD |
| 49 | M | Mod/Sev | unknown | unknown | Y | N | phenytoin 35omg OD, Omnitrope GH 0.3mg/0.4mg,  Bendroflumethiazide 2.5mg, Thiamine 300mg OD,  Quetiapine 100mg OD, Mirtazapine 45mg OD,  Atorvastatin 40mg OD, Amlodipine 10mg OD,  Ramipril 7.5mg OD, Pregabalin 300mg OD |
| 72 | M | Mod/Sev | unknown | unknown | Y | N | nil |
| 40 | F | Mod/Sev | RTA | unknown | Y | N | Tegretol, propranolol |
| 65 | M | Mod/Sev | incident fall | 14 days | N | N | amlodipine, simvastatin, ramipril, |
| 54 | M | Mod/Sev | RTA | 10 days | Y | Y | Tegretol 200mg BD, Amitriptyline 100mg |
| 56 | F | Mod/Sev | RTA | several months | Y | N | Ventolin, Seretide |
| 43 | M | Mod/Sev | RTA | unknown | Y | N | pregabalin 75mg bd, Ventolin, cetirizine, omeprazole, lamotrigine 50mg bd |
| 39 | M | Mod/Sev | RTA | unknown | Y | N | Tegretol 400mg bd |
| 57 | M | Mod/Sev | RTA | unknown | Y | N | nil |
| 31 | F | Mod/Sev | unknown | unknown | unknown | unknown | unknown |
| 38 | M | Mod/Sev | unknown | unknown | unknown | unknown | unknown |
| 51 | M | Mod/Sev | incident fall | unknown | unknown | unknown | nil |
| 54 | M | Mod/Sev | RTA | 1 days | N | N | nil |
| 46 | F | Mod/Sev | incident fall | 4 weeks | unknown | unknown | nil |
| 43 | M | Mod/Sev | RTA | no | N | N | nil |
| 55 | M | Mod/Sev | violence/assault | 4 days | N | N | Levetiracetam, Salbutamol |
| 48 | M | Mod/Sev | RTA | no | N | N | Salbutamol, Seretide |
| 57 | M | Mod/Sev | RTA | no | N | N | nil |

**Supplementary table 1: Demographics of TBI patients.** M=Male, F=Female, Mod/Sev=Moderate-severe TBI injury. RTA=Road traffic accident. PTA=Post traumatic amnesia. N=No, Y=Yes. OD=Once daily. BD=twice a day.

| **Domain** | **Task** | **Control mean**  **n=35** | **Patient mean**  **N=92** | **Difference** |
| --- | --- | --- | --- | --- |
| **Intellectual ability** | WTAR Scaled | 114.8 | 108.3 | **6.5**** |
|  | Matrix reasoning | 27.76 | 26.93 | 0.83 |
| **Information processing** | Trail Making A | 20.55 | 32.49 | **-11.94**** |
|  | Stroop Word reading | 20.41 | 25.18 | **-4.77**** |
|  | Stroop colour naming time | 27.95 | 34.92 | **-6.97**** |
| **Executive function** | Trail Making B minus A | -0.47 | 0.07 | **0.54**** |
|  | Stroop Inhib time | 52.77 | 60.59 | **-7.82*** |
|  | Stroop Inhib switch time | 57.2 | 71.02 | **-13.82**** |
|  | Stroop contrast inhib vs colour naming | 24.83 | 25.66 | -0.83 |
|  | Stroop Contrast Inhib switch vs Inhib | 4.43 | 10.44 | -6.01 |
|  | Stroop Contrast Inhib switch vs combi baseline | 34.91 | 39.99 | -5.08 |
| **Memory** | Peoples Test total | 27.58 | 22.82 | **4.76*** |
|  | Peoples test delayed | 9.79 | 8.18 | **1.61*** |
|  | Logical memory 1 total | 50.26 | 38.35 | **11.91**** |
|  | Logical memory 2 total | 32.55 | 23.24 | **9.31**** |
|  | Logical memory retention | 89.62 | 80.42 | **9.2*** |

**Supplementary table 2: Standard neuropsychological test battery performance for patients and controls.** Data in bold represent significant differences in performance between patients and controls as quantified using post-hoc Tukey HSD contrasts from a repeated measures ANOVA. *=p<0.05, **=p<0.01, ***=p<0.001. WTAR=Weschler test of adult reading.

| **Working Memory network nodes** | **Reasoning network nodes** |
| --- | --- |
| Caudate.L**^✝^** | CaudMidFront.L |
| Pallidum.L | Cuneus.L |
| Hippocampus.L | Fusiform.L |
| Caudate.R | InfParietal.L |
| Pallidum.R**^✝^** | InfTemporal.L |
| Hippocampus.R | LatOcc.L |
| CaudAntCing.L | **LatOrbFront.L** |
| **LatOrbitoFront.L** | ParsOperc.L |
| Paracentral.L | Pericalcarine.L |
| PostCentral.L | **Precentral.L** |
| PostCingulate.L | **RosMidFront.L^✝^** |
| **Precentral.L** | **SupFront.L^✝^** |
| Precuneus.L | SupParietal.L**^✝^** |
| RosAntCing.L | **CaudMidFront.R** |
| **RosMidFront.L** | Cuneus.R |
| **SupFront.L** | Fusiform.R |
| Insula.L**^✝^** | InfParietal.R**^✝^** |
| CaudaAntCing.R | InfTemporal.R |
| **CaudMidFront.R** | LateralOcc.R |
| LatOrbitoFront.R | Lingual.R |
| Paracentral.R | ParsOperc.R |
| PostCentral.R | Paricalcarine.R |
| PostCingulate.R | **Precentral.R** |
| **Precentral.R** | **RosMidFront.R** |
| Precuneus.R**^✝^** | **SupFront.R** |
| RosAntCing.R | **SupParietal.R^✝^** |
| **RosMidFront.R** |  |
| **SupFront.R** |  |
| **SupParietal.R^✝^** |  |
| SupraMarg.R |  |
| Insula.R**^✝^** |  |

**Supplementary Table 3: List of network nodes for working memory and reasoning networks defined using activation maps from Hampshire *et al*., 2012. ^✝^**Network Hub as defined using node degree (highest 20% degree in healthy controls). Highlighted nodes in bold indicate overlap between working memory and reasoning networks.

| **Working memory network** | | | | | | | | | |
| --- | --- | --- | --- | --- | --- | --- | --- | --- | --- |
|  | Local Efficiency | | | Degree | | | Clustering coefficient | | |
| Node | Con | Pat | P(FDR) | Con | Pat | P(FDR) | Con | PAT | P(FDR) |
| Caudate.L^✝^ | 16815.549 | 20236.534 | 0.000*** | 29.434 | 26.478 | 0.000*** | 12666.228 | 15931.769 | 0.000*** |
| Pallidum.L | 16007.302 | 17985.204 | 0.000*** | 28.660 | 26.261 | 0.000*** | 11149.220 | 12062.893 | 0.001** |
| Hippocampus.L | 8747.993 | 9842.603 | 0.000*** | 26.698 | 24.837 | 0.000*** | 6290.153 | 7392.492 | 0.000*** |
| Caudate.R | 21100.774 | 25168.573 | 0.000*** | 29.736 | 27.000 | 0.000*** | 15984.150 | 20201.510 | 0.000*** |
| Pallidum.R^✝^ | 24084.156 | 26877.096 | 0.000*** | 29.604 | 27.315 | 0.000*** | 17604.054 | 19070.478 | 0.000*** |
| Hippocampus.R | 6149.067 | 6568.627 | 0.000*** | 26.698 | 25.337 | 0.000*** | 4062.609 | 4388.791 | 0.001** |
| CaudAntCing.L | 6859.146 | 6793.239 | 0.579 | 25.717 | 23.609 | 0.000*** | 5603.492 | 5700.563 | 0.551 |
| LatOrbitoFront.L | 13498.172 | 15146.114 | 0.000*** | 27.717 | 25.065 | 0.000*** | 10111.978 | 11650.011 | 0.000*** |
| Paracentral.L | 31543.029 | 34137.437 | 0.000*** | 26.585 | 23.315 | 0.000*** | 22102.203 | 23919.350 | 0.023* |
| PostCentral.L | 23411.077 | 26636.278 | 0.000*** | 26.689 | 23.804 | 0.000*** | 16277.600 | 19546.049 | 0.000*** |
| PostCingulate.L | 10519.887 | 11387.525 | 0.000*** | 24.670 | 22.076 | 0.000*** | 6651.602 | 7963.272 | 0.000*** |
| Precentral.L | 40576.919 | 43968.222 | 0.000*** | 26.679 | 23.424 | 0.000*** | 28909.212 | 28470.366 | 0.583 |
| Precuneus.L | 25475.842 | 28309.738 | 0.000*** | 27.679 | 24.500 | 0.000*** | 17723.149 | 20382.424 | 0.002** |
| RosAntCing.L | 4499.403 | 4032.585 | 0.000*** | 22.858 | 21.783 | 0.000*** | 4836.203 | 4727.655 | 0.487 |
| RosMidFront.L | 34091.968 | 37622.774 | 0.000*** | 29.519 | 26.228 | 0.000*** | 24219.030 | 27691.741 | 0.002** |
| SupFront.L | 52953.337 | 55941.815 | 0.000*** | 29.623 | 25.935 | 0.000*** | 37634.545 | 38086.021 | 0.647 |
| Insula.L^✝^ | 27615.340 | 30807.954 | 0.000*** | 28.623 | 26.500 | 0.000*** | 20551.918 | 22759.488 | 0.000*** |
| CaudaAntCing.R | 6179.156 | 6248.551 | 0.574 | 24.736 | 23.891 | 0.000*** | 5551.114 | 7569.185 | 0.004** |
| CaudMidFront.R | 20421.489 | 20600.807 | 0.352 | 24.689 | 22.870 | 0.000*** | 13967.221 | 13694.287 | 0.402 |
| LatOrbitoFront.R | 11702.886 | 12391.604 | 0.000*** | 29.764 | 27.250 | 0.000*** | 9890.857 | 11310.579 | 0.000*** |
| Paracentral.R | 36704.269 | 42028.098 | 0.000*** | 27.660 | 24.489 | 0.000*** | 27810.266 | 33244.109 | 0.012* |
| PostCentral.R | 30942.906 | 35907.345 | 0.000*** | 27.708 | 24.804 | 0.000*** | 23980.939 | 26897.134 | 0.001** |
| PostCingulate.R | 9346.930 | 10073.217 | 0.000*** | 27.774 | 25.902 | 0.000*** | 7753.104 | 15332.966 | 0.054 |
| Precentral.R | 44466.331 | 47485.613 | 0.000*** | 26.604 | 24.011 | 0.000*** | 31718.234 | 31602.625 | 0.854 |
| Precuneus.R^✝^ | 25194.630 | 25889.898 | 0.003** | 28.689 | 26.989 | 0.000*** | 17485.324 | 18971.761 | 0.005** |
| RosAntCing.R | 4610.147 | 4444.214 | 0.000*** | 20.868 | 19.891 | 0.000*** | 3755.706 | 3702.175 | 0.504 |
| RosMidFront.R | 23123.279 | 26133.465 | 0.000*** | 29.679 | 26.283 | 0.000*** | 16973.678 | 20494.366 | 0.006** |
| SupFront.R | 48482.950 | 50489.749 | 0.000*** | 29.679 | 26.326 | 0.000*** | 34139.131 | 36110.027 | 0.158 |
| SupParietal.R^✝^ | 29222.546 | 30795.403 | 0.000*** | 27.594 | 25.446 | 0.000*** | 20701.982 | 21328.062 | 0.087 |
| SupraMarg.R | 25978.043 | 28959.918 | 0.000*** | 28.660 | 25.880 | 0.000*** | 19039.778 | 20240.209 | 0.005** |
| Insula.R^✝^ | 21363.536 | 23795.822 | 0.000*** | 29.660 | 27.304 | 0.000*** | 16754.316 | 18462.856 | 0.002** |

**Supplementary table 4: Local/nodal network properties of patients and controls in the working memory network.** ✝Network hubs as classified by determining top 20% of nodes with highest degree across healthy population at threshold of 2.5 S.D.

| **Reasoning network** | | | | | | | | | |
| --- | --- | --- | --- | --- | --- | --- | --- | --- | --- |
|  | Local Efficiency | | | Degree | | | Clustering Coefficient | | |
| Node | Con | Pat | FDR | Con | Pat | FDR | Con | Pat | FDR |
| CaudMidFront.L | 45143.064 | 44334.101 | 0.008** | 21.792 | 20.641 | 0.000*** | 39036.915 | 37322.438 | 0.004** |
| Cuneus.L | 8098.240 | 8464.496 | 0.008** | 19.896 | 19.033 | 0.000*** | 5960.671 | 6481.295 | 0.005** |
| Fusiform.L | 17083.379 | 18696.326 | 0.000*** | 24.708 | 23.793 | 0.000*** | 15571.427 | 18407.135 | 0.001** |
| InfParietal.L | 29577.958 | 33073.872 | 0.000*** | 24.736 | 23.098 | 0.000*** | 24823.057 | 27423.823 | 0.003** |
| InfTemporal.L | 24712.372 | 28086.847 | 0.000*** | 24.689 | 23.478 | 0.000*** | 21018.432 | 26710.216 | 0.003** |
| LatOcc.L | 20438.961 | 22437.616 | 0.001** | 24.868 | 23.739 | 0.000*** | 18094.638 | 20881.697 | 0.002** |
| LatOrbFront.L | 11029.768 | 11370.555 | 0.000*** | 24.679 | 23.554 | 0.000*** | 9940.590 | 10759.964 | 0.001** |
| ParsOperc.L | 37997.545 | 38808.974 | 0.022* | 19.745 | 18.185 | 0.000*** | 30469.375 | 29968.675 | 0.245 |
| Pericalcarine.L | 9011.637 | 9705.834 | 0.001** | 20.868 | 20.033 | 0.000*** | 7766.978 | 8751.377 | 0.000*** |
| Precentral.L | 44359.494 | 45466.625 | 0.079 | 21.849 | 20.620 | 0.000*** | 36580.574 | 37157.042 | 0.567 |
| RosMidFront.L^✝^ | 41476.861 | 42062.625 | 0.191 | 23.745 | 22.326 | 0.000*** | 35885.362 | 37641.653 | 0.068 |
| SupFront.L^✝^ | 53626.504 | 50157.573 | 0.000*** | 22.679 | 21.337 | 0.000*** | 42650.488 | 39598.898 | 0.001** |
| SupParietal.L^✝^ | 32088.953 | 35690.365 | 0.000*** | 24.736 | 22.880 | 0.000*** | 23379.730 | 27864.039 | 0.001** |
| CaudMidFront.R | 28904.806 | 27657.205 | 0.010* | 22.689 | 21.174 | 0.000*** | 24191.172 | 22219.897 | 0.000*** |
| Cuneus.R | 12546.965 | 13544.679 | 0.001** | 17.877 | 16.880 | 0.000*** | 9455.167 | 10563.770 | 0.009** |
| Fusiform.R | 22244.117 | 24520.766 | 0.000*** | 23.783 | 22.457 | 0.000*** | 18225.608 | 20160.950 | 0.002** |
| InfParietal.R^✝^ | 21473.320 | 21728.667 | 0.251 | 24.575 | 23.065 | 0.000*** | 16235.403 | 15821.861 | 0.070 |
| InfTemporal.R | 16802.134 | 17683.229 | 0.022* | 23.613 | 21.978 | 0.000*** | 12770.564 | 12973.215 | 0.567 |
| LateralOcc.R | 19040.846 | 21358.076 | 0.000*** | 20.811 | 19.500 | 0.000*** | 13784.563 | 15711.671 | 0.000*** |
| Lingual.R | 16853.564 | 19239.613 | 0.000*** | 21.849 | 20.609 | 0.000*** | 14119.562 | 17048.882 | 0.000*** |
| ParsOperc.R | 23408.375 | 22493.908 | 0.035* | 20.755 | 19.467 | 0.000*** | 19797.425 | 18565.069 | 0.002** |
| Paricalcarine.R | 20198.020 | 22761.774 | 0.000*** | 20.858 | 19.826 | 0.000*** | 17317.791 | 21352.841 | 0.001** |
| Precentral.R | 28421.789 | 27105.584 | 0.001** | 22.736 | 21.261 | 0.000*** | 24187.744 | 22030.213 | 0.000*** |
| RosMidFront.R | 24968.764 | 24514.426 | 0.204 | 19.660 | 18.239 | 0.000*** | 17455.188 | 17226.701 | 0.567 |
| SupFront.R | 45786.966 | 42725.445 | 0.002** | 22.726 | 20.837 | 0.000*** | 35565.529 | 34430.753 | 0.310 |
| SupParietal.R^✝^ | 20924.338 | 21746.199 | 0.002** | 24.726 | 23.054 | 0.000*** | 14784.840 | 15235.855 | 0.096 |

**Supplementary table 5: Local/nodal network properties of patients and controls in the working memory network.** ✝Network hubs as classified by determining top 20% of nodes with highest degree across healthy population at threshold of 2.5 S.D.

**Working memory network correlations to cognitive components across all structural connectome threshold iterations (see supplementary figure 9 for graphical illustration of results)**

**0.5 SD units**

| GT Metrics | Degree centrality | | | | Local Efficiency | | | | Clustering coefficient | | | |
| --- | --- | --- | --- | --- | --- | --- | --- | --- | --- | --- | --- | --- |
| Node | Reasoning  R. p(FDR) | | Working mem  R. p(FDR) | | Reasoning  R. p(FDR) | | Working mem  R. p(FDR) | | Reasoning  R. p(FDR) | | Working mem.  R. p(FDR) | |
| Caudate.L^✝^ | 0.081 | 0.839 | 0.116 | 0.761 | -0.322 | 0.018* | -0.214 | 0.238 | -0.247 | 0.068 | -0.190 | 0.258 |
| Pallidum.L | 0.056 | 0.839 | 0.109 | 0.774 | -0.308 | 0.022* | -0.139 | 0.388 | -0.246 | 0.068 | -0.177 | 0.294 |
| Hippocampus.L | 0.013 | 0.963 | -0.071 | 0.958 | -0.268 | 0.032* | -0.246 | 0.158 | -0.379 | 0.005* | -0.242 | 0.203 |
| Caudate.R | 0.116 | 0.794 | 0.020 | 0.997 | -0.209 | 0.075 | -0.241 | 0.158 | -0.191 | 0.160 | -0.214 | 0.203 |
| Pallidum.R^✝^ | 0.051 | 0.845 | 0.086 | 0.865 | -0.232 | 0.051 | -0.251 | 0.158 | -0.156 | 0.267 | -0.258 | 0.203 |
| Hippocampus.R | 0.025 | 0.925 | -0.064 | 0.958 | -0.269 | 0.032* | -0.179 | 0.271 | -0.299 | 0.028* | -0.160 | 0.334 |
| CaudAntCing.L | 0.073 | 0.839 | 0.143 | 0.594 | -0.035 | 0.789 | 0.182 | 0.271 | NaN | NaN | NaN | NaN |
| LatOrbitoFront.L | -0.062 | 0.839 | 0.003 | 0.997 | -0.289 | 0.026* | -0.254 | 0.158 | -0.024 | 0.857 | -0.172 | 0.294 |
| Paracentral.L | 0.083 | 0.839 | 0.001 | 0.997 | -0.231 | 0.051 | -0.078 | 0.559 | NaN | NaN | NaN | NaN |
| PostCentral.L | 0.056 | 0.839 | -0.012 | 0.997 | -0.285 | 0.026* | -0.097 | 0.559 | -0.295 | 0.028* | -0.208 | 0.203 |
| PostCingulate.L | 0.090 | 0.839 | 0.096 | 0.865 | -0.108 | 0.377 | -0.038 | 0.746 | -0.126 | 0.376 | -0.098 | 0.708 |
| Precentral.L | 0.005 | 0.989 | -0.001 | 0.997 | -0.245 | 0.044* | -0.087 | 0.559 | -0.144 | 0.295 | -0.116 | 0.583 |
| Precuneus.L | 0.114 | 0.794 | 0.154 | 0.550 | -0.200 | 0.082 | -0.153 | 0.319 | -0.228 | 0.093 | -0.214 | 0.203 |
| RosAntCing.L | -0.141 | 0.794 | 0.062 | 0.958 | 0.008 | 0.943 | 0.084 | 0.559 | -0.029 | 0.852 | -0.011 | 0.974 |
| RosMidFront.L | 0.089 | 0.839 | 0.132 | 0.652 | -0.172 | 0.136 | -0.072 | 0.571 | 0.000 | 0.998 | -0.043 | 0.893 |
| SupFront.L | 0.113 | 0.794 | 0.190 | 0.433 | -0.332 | 0.018* | -0.096 | 0.559 | -0.182 | 0.177 | -0.006 | 0.974 |
| Insula.L | -0.064 | 0.839 | 0.205 | 0.383 | -0.251 | 0.040* | -0.120 | 0.491 | -0.222 | 0.098 | 0.008 | 0.974 |
| CaudaAntCing.R | 0.085 | 0.839 | 0.209 | 0.383 | -0.027 | 0.827 | 0.066 | 0.571 | -0.091 | 0.536 | 0.074 | 0.828 |
| CaudMidFront.R | -0.024 | 0.925 | 0.000 | 0.997 | -0.105 | 0.379 | -0.110 | 0.540 | -0.090 | 0.536 | 0.007 | 0.974 |
| LatOrbitoFront.R | 0.028 | 0.925 | 0.226 | 0.383 | -0.159 | 0.167 | -0.160 | 0.311 | 0.097 | 0.536 | -0.079 | 0.828 |
| Paracentral.R | 0.147 | 0.794 | 0.045 | 0.997 | -0.211 | 0.075 | -0.159 | 0.311 | NaN | NaN | NaN | NaN |
| PostCentral.R | 0.152 | 0.794 | 0.053 | 0.997 | -0.258 | 0.036* | -0.196 | 0.238 | -0.198 | 0.153 | -0.131 | 0.502 |
| PostCingulate.R | 0.058 | 0.839 | -0.085 | 0.865 | -0.198 | 0.083 | -0.076 | 0.559 | NaN | NaN | NaN | NaN |
| Precentral.R | 0.027 | 0.925 | -0.027 | 0.997 | -0.067 | 0.596 | -0.089 | 0.559 | 0.062 | 0.684 | -0.061 | 0.863 |
| Precuneus.R | 0.137 | 0.794 | 0.030 | 0.997 | -0.266 | 0.032* | -0.067 | 0.571 | -0.154 | 0.267 | -0.045 | 0.893 |
| RosAntCing.R | -0.124 | 0.794 | 0.240 | 0.383 | 0.065 | 0.596 | 0.026 | 0.804 | 0.036 | 0.826 | -0.070 | 0.828 |
| RosMidFront.R | 0.127 | 0.794 | 0.001 | 0.997 | -0.203 | 0.081 | -0.078 | 0.559 | -0.263 | 0.059 | -0.003 | 0.974 |
| SupFront.R | 0.022 | 0.925 | 0.019 | 0.997 | -0.242 | 0.045* | -0.086 | 0.559 | NaN | NaN | NaN | NaN |
| SupParietal.R | 0.001 | 0.989 | -0.040 | 0.997 | -0.229 | 0.051 | -0.167 | 0.311 | -0.059 | 0.684 | -0.045 | 0.893 |
| SupraMarg.R | 0.173 | 0.794 | 0.172 | 0.525 | -0.295 | 0.026* | -0.196 | 0.238 | -0.320 | 0.025* | -0.223 | 0.203 |
| Insula.R | 0.131 | 0.794 | 0.155 | 0.550 | -0.350 | 0.018* | -0.204 | 0.238 | -0.076 | 0.615 | -0.006 | 0.974 |

**Supplementary table 6: Correlation coefficients and p-values for the relationship between cognitive components and graph theory metrics of a given node within the working memory network constructed using a 0.5 SD thresholding approach.** R=Pearson’s r, p(FDR)=p-value with FDR correction applied across nodes.

**1.0 SD units**

| GT Metrics | Degree | | | | Local Efficiency | | | | Clustering coefficient | | | |
| --- | --- | --- | --- | --- | --- | --- | --- | --- | --- | --- | --- | --- |
| Node | Reasoning  R. p(FDR) | | Working mem  R. p(FDR) | | Reasoning  R. p(FDR) | | Working mem.  R. p(FDR) | | Reasoning  R. p(FDR) | | Working mem.  R. p(FDR) | |
| Caudate.L | 0.126 | 0.472 | 0.186 | 0.172 | -0.300 | 0.020* | -0.258 | 0.067 | -0.283 | 0.032* | -0.231 | 0.124 |
| Pallidum.L | 0.076 | 0.586 | 0.214 | 0.172 | -0.270 | 0.032* | -0.183 | 0.192 | -0.314 | 0.016* | -0.162 | 0.310 |
| Hippocampus.L | 0.062 | 0.663 | 0.041 | 0.772 | -0.264 | 0.032 | -0.230 | 0.105 | -0.362 | 0.011* | -0.245 | 0.124 |
| Caudate.R | 0.110 | 0.472 | 0.122 | 0.391 | -0.198 | 0.100 | -0.272 | 0.067 | -0.137 | 0.330 | -0.267 | 0.124 |
| Pallidum.R | 0.138 | 0.454 | 0.185 | 0.172 | -0.190 | 0.102 | -0.261 | 0.067 | -0.159 | 0.306 | -0.293 | 0.124 |
| Hippocampus.R | 0.085 | 0.544 | 0.041 | 0.772 | -0.299 | 0.020* | -0.206 | 0.162 | -0.334 | 0.016* | -0.157 | 0.315 |
| CaudAntCing.L | 0.217 | 0.223 | 0.188 | 0.172 | -0.001 | 0.992 | 0.157 | 0.234 | -0.205 | 0.141 | 0.094 | 0.653 |
| LatOrbitoFront.L | 0.028 | 0.876 | 0.171 | 0.215 | -0.354 | 0.010* | -0.290 | 0.067 | -0.056 | 0.727 | -0.217 | 0.131 |
| Paracentral.L | 0.105 | 0.472 | 0.114 | 0.396 | -0.280 | 0.027* | -0.098 | 0.419 | NaN | NaN | NaN | NaN |
| PostCentral.L | 0.111 | 0.472 | 0.020 | 0.912 | -0.256 | 0.035* | -0.126 | 0.313 | -0.316 | 0.016* | -0.246 | 0.124 |
| PostCingulate.L | 0.136 | 0.454 | 0.207 | 0.172 | -0.109 | 0.373 | 0.025 | 0.837 | -0.129 | 0.330 | -0.012 | 0.911 |
| Precentral.L | 0.057 | 0.673 | 0.117 | 0.396 | -0.179 | 0.118 | -0.165 | 0.234 | -0.131 | 0.330 | -0.171 | 0.310 |
| Precuneus.L | 0.133 | 0.454 | 0.189 | 0.172 | -0.234 | 0.048* | -0.143 | 0.256 | -0.119 | 0.361 | -0.101 | 0.634 |
| RosAntCing.L | 0.004 | 0.967 | 0.085 | 0.522 | 0.057 | 0.640 | 0.067 | 0.580 | -0.030 | 0.863 | -0.027 | 0.911 |
| RosMidFront.L | 0.212 | 0.223 | 0.158 | 0.259 | -0.186 | 0.108 | -0.119 | 0.332 | 0.011 | 0.918 | -0.143 | 0.372 |
| SupFront.L | 0.153 | 0.448 | 0.208 | 0.172 | -0.300 | 0.020* | -0.200 | 0.162 | -0.222 | 0.104 | -0.041 | 0.911 |
| Insula.L | 0.111 | 0.472 | 0.206 | 0.172 | -0.262 | 0.032* | -0.179 | 0.196 | -0.255 | 0.050 | -0.082 | 0.722 |
| CaudaAntCing.R | 0.106 | 0.472 | 0.263 | 0.172 | 0.079 | 0.521 | 0.059 | 0.613 | -0.027 | 0.863 | 0.052 | 0.911 |
| CaudMidFront.R | 0.122 | 0.472 | 0.188 | 0.172 | -0.114 | 0.361 | -0.199 | 0.162 | -0.128 | 0.330 | -0.047 | 0.911 |
| LatOrbitoFront.R | 0.024 | 0.876 | 0.200 | 0.172 | -0.249 | 0.039* | -0.126 | 0.313 | -0.079 | 0.602 | -0.038 | 0.911 |
| Paracentral.R | 0.211 | 0.223 | 0.073 | 0.585 | -0.193 | 0.100 | -0.154 | 0.234 | NaN | NaN | NaN | NaN |
| PostCentral.R | 0.226 | 0.223 | 0.130 | 0.375 | -0.247 | 0.039* | -0.244 | 0.083 | -0.158 | 0.306 | -0.234 | 0.124 |
| PostCingulate.R | 0.176 | 0.319 | 0.013 | 0.915 | -0.047 | 0.677 | -0.160 | 0.234 | NaN | NaN | NaN | NaN |
| Precentral.R | 0.094 | 0.523 | -0.011 | 0.915 | -0.056 | 0.640 | -0.081 | 0.506 | 0.030 | 0.863 | -0.016 | 0.911 |
| Precuneus.R | 0.268 | 0.223 | 0.088 | 0.519 | -0.196 | 0.100 | -0.149 | 0.242 | 0.017 | 0.907 | -0.065 | 0.841 |
| RosAntCing.R | 0.009 | 0.962 | 0.244 | 0.172 | 0.099 | 0.414 | -0.006 | 0.958 | 0.076 | 0.602 | -0.017 | 0.911 |
| RosMidFront.R | 0.187 | 0.289 | 0.139 | 0.338 | -0.244 | 0.040* | -0.117 | 0.332 | -0.268 | 0.039* | -0.014 | 0.911 |
| SupFront.R | 0.088 | 0.544 | 0.121 | 0.391 | -0.196 | 0.100 | -0.156 | 0.234 | -0.140 | 0.330 | 0.012 | 0.911 |
| SupParietal.R | 0.145 | 0.454 | 0.095 | 0.498 | -0.280 | 0.027* | -0.193 | 0.168 | -0.145 | 0.330 | -0.135 | 0.398 |
| SupraMarg.R | 0.188 | 0.289 | 0.210 | 0.172 | -0.300 | 0.020* | -0.266 | 0.067 | -0.280 | 0.032* | -0.217 | 0.131 |
| Insula.R | 0.229 | 0.223 | 0.285 | 0.172 | -0.350 | 0.010* | -0.278 | 0.067 | -0.139 | 0.330 | -0.164 | 0.310 |

**Supplementary table 7: Correlation coefficients and p-values for the relationship between cognitive components and graph theory metrics of a given node within the working memory network constructed using a 1.0 SD thresholding approach.** R=Pearson’s r, p(FDR)=p-value with FDR correction applied across nodes.

- 1. SD units

| GT Metrics | Degree | | | | Local Efficiency | | | | Clustering coefficient | | | |
| --- | --- | --- | --- | --- | --- | --- | --- | --- | --- | --- | --- | --- |
| Node | Reasoning  R. p(FDR) | | Working mem.  R. p(FDR) | | Reasoning  R. p(FDR) | | Working mem.  R. p(FDR) | | Reasoning  R. p(FDR) | | Working mem.  R. p(FDR) | |
| Caudate.L | 0.173 | 0.175 | 0.270 | 0.067 | -0.324 | 0.012* | -0.297 | 0.051 | -0.318 | 0.016* | -0.213 | 0.109 |
| Palidum.L | 0.169 | 0.175 | 0.219 | 0.80 | -0.314 | 0.012* | -0.266 | 0.065 | -0.322 | 0.016* | -0.236 | 0.083 |
| Hippocampus.L | 0.140 | 0.240 | 0.192 | 0.104 | -0.232 | 0.048* | -0.275 | 0.063 | -0.359 | 0.013* | -0.254 | 0.083 |
| Caudate.R | 0.134 | 0.240 | 0.253 | 0.067 | -0.241 | 0.043* | -0.241 | 0.079 | -0.248 | 0.045* | -0.248 | 0.083 |
| Pallidum.R | 0.208 | 0.104 | 0.255 | 0.067 | -0.202 | 0.085 | -0.232 | 0.089 | -0.133 | 0.338 | -0.228 | 0.083 |
| Hippocampus.R | 0.240 | 0.071 | 0.111 | 0.300 | -0.288 | 0.021* | -0.157 | 0.233 | -0.297 | 0.020* | -0.139 | 0.324 |
| CaudAntCing.L | 0.244 | 0.071 | 0.241 | 0.068 | -0.023 | 0.882 | 0.110 | 0.385 | -0.206 | 0.095 | 0.092 | 0.586 |
| LatOrbitoFront.L | 0.098 | 0.393 | 0.242 | 0.068 | -0.327 | 0.012* | -0.308 | 0.051 | -0.245 | 0.045* | -0.242 | 0.083 |
| Paracentral.L | 0.162 | 0.189 | 0.173 | 0.133 | -0.265 | 0.027* | -0.158 | 0.233 | -0.074 | 0.581 | -0.176 | 0.208 |
| PostCentral.L | 0.113 | 0.325 | 0.120 | 0.274 | -0.278 | 0.023* | -0.189 | 0.157 | -0.316 | 0.016* | -0.259 | 0.083 |
| PostCingulate.L | 0.191 | 0.142 | 0.210 | 0.080 | -0.084 | 0.482 | -0.121 | 0.370 | -0.064 | 0.628 | -0.168 | 0.226 |
| Precentral.L | 0.145 | 0.235 | 0.141 | 0.225 | -0.164 | 0.160 | -0.243 | 0.079 | -0.102 | 0.461 | -0.240 | 0.083 |
| Precuneus.L | 0.156 | 0.204 | 0.185 | 0.115 | -0.150 | 0.199 | -0.190 | 0.157 | -0.233 | 0.053 | -0.244 | 0.083 |
| RosAntCing.L | 0.074 | 0.484 | 0.133 | 0.239 | 0.099 | 0.415 | 0.068 | 0.555 | 0.049 | 0.710 | 0.009 | 0.979 |
| RosMidFront.L | 0.232 | 0.071 | 0.226 | 0.072 | -0.281 | 0.023* | -0.157 | 0.233 | -0.132 | 0.338 | -0.140 | 0.324 |
| SupFront.L | 0.217 | 0.091 | 0.232 | 0.068 | -0.320 | 0.012* | -0.149 | 0.255 | -0.249 | 0.045* | -0.009 | 0.979 |
| Insula.L | 0.135 | 0.240 | 0.232 | 0.068 | -0.262 | 0.027* | -0.200 | 0.145 | -0.292 | 0.020* | -0.101 | 0.545 |
| CaudaAntCing.R | 0.231 | 0.071 | 0.239 | 0.068 | -0.013 | 0.934 | 0.042 | 0.702 | -0.293 | 0.020* | -0.037 | 0.913 |
| CaudMidFront.R | 0.171 | 0.175 | 0.211 | 0.080 | -0.082 | 0.482 | -0.040 | 0.702 | -0.093 | 0.496 | 0.060 | 0.786 |
| LatOrbitoFront.R | 0.177 | 0.175 | 0.265 | 0.067 | -0.310 | 0.012* | -0.129 | 0.341 | -0.109 | 0.438 | -0.065 | 0.778 |
| Paracentral.R | 0.243 | 0.071 | 0.163 | 0.155 | -0.168 | 0.156 | -0.165 | 0.233 | NaN | NaN | NaN | NaN |
| PostCentral.R | 0.266 | 0.065 | 0.192 | 0.104 | -0.244 | 0.042* | -0.225 | 0.097 | -0.116 | 0.413 | -0.231 | 0.083 |
| PostCingulate.R | 0.286 | 0.054 | 0.122 | 0.271 | -0.100 | 0.415 | -0.110 | 0.385 | NaN | NaN | NaN | NaN |
| Precentral.R | 0.089 | 0.427 | 0.095 | 0.366 | 0.002 | 0.982 | -0.084 | 0.526 | 0.043 | 0.710 | -0.047 | 0.866 |
| Precuneus.R | 0.320 | 0.040* | 0.174 | 0.133 | -0.232 | 0.048* | -0.110 | 0.385 | 0.046 | 0.710 | -0.020 | 0.979 |
| RosAntCing.R | 0.082 | 0.449 | 0.265 | 0.067 | 0.201 | 0.085 | 0.071 | 0.554 | 0.090 | 0.496 | -0.003 | 0.979 |
| RosMidFront.R | 0.230 | 0.071 | 0.211 | 0.080 | -0.268 | 0.027* | -0.071 | 0.554 | -0.281 | 0.024* | 0.005 | 0.979 |
| SupFront.R | 0.136 | 0.240 | 0.192 | 0.104 | -0.199 | 0.085 | -0.078 | 0.551 | -0.240 | 0.047* | 0.026 | 0.976 |
| SupParietal.R | 0.249 | 0.071 | 0.133 | 0.239 | -0.225 | 0.053 | -0.202 | 0.145 | 0.008 | 0.939 | -0.138 | 0.324 |
| SupraMarg.R | 0.280 | 0.054 | 0.270 | 0.067 | -0.349 | 0.010* | -0.247 | 0.079 | -0.274 | 0.026* | -0.178 | 0.208 |
| Insula.R | 0.311 | 0.040* | 0.277 | 0.067 | -0.349 | 0.010* | -0.290 | 0.051 | -0.161 | 0.228 | -0.235 | 0.083 |

**Supplementary table 8: Correlation coefficients and p-values for the relationship between cognitive components and graph theory metrics of a given node within the working memory network constructed using a 1.5 SD thresholding approach.** R=Pearson’s r, p(FDR)=p-value with FDR correction applied across nodes.

2.0 SD units

| GT Metrics | Degree | | | | Local Efficiency | | | | Clustering coefficient | | | |
| --- | --- | --- | --- | --- | --- | --- | --- | --- | --- | --- | --- | --- |
| Node | Reasoning  R. p(FDR) | | Working mem.  R. p(FDR) | | Reasoning  R. p(FDR) | | Working mem.  R. p(FDR) | | Reasoning  R. p(FDR) | | Working mem.  R. p(FDR) | |
| Caudate.L | 0.188 | 0.087 | 0.293 | 0.037* | -0.291 | 0.020* | -0.294 | 0.048* | -0.327 | 0.019* | -0.213 | 0.125 |
| Pallidum.L | 0.179 | 0.100 | 0.238 | 0.055 | -0.245 | 0.041* | -0.250 | 0.056 | -0.270 | 0.039* | -0.212 | 0.125 |
| Hippocampus.L | 0.204 | 0.084 | 0.211 | 0.064 | -0.258 | 0.031* | -0.264 | 0.048* | -0.357 | 0.015* | -0.294 | 0.053 |
| Caudate.R | 0.190 | 0.087 | 0.298 | 0.037* | -0.260 | 0.031* | -0.260 | 0.048* | -0.280 | 0.039* | -0.258 | 0.081 |
| Pallidum.R | 0.221 | 0.076 | 0.279 | 0.037* | -0.216 | 0.063 | -0.270 | 0.048* | -0.184 | 0.134 | -0.261 | 0.081 |
| Hippocampus.R | 0.324 | 0.025* | 0.173 | 0.116 | -0.242 | 0.041* | -0.180 | 0.147 | -0.260 | 0.040* | -0.167 | 0.229 |
| CaudAntCing.L | 0.292 | 0.029* | 0.227 | 0.055 | -0.082 | 0.466 | 0.081 | 0.474 | -0.255 | 0.040* | 0.041 | 0.864 |
| LatOrbitoFront.L | 0.189 | 0.087 | 0.223 | 0.056 | -0.290 | 0.020* | -0.276 | 0.048* | -0.200 | 0.102 | -0.238 | 0.114 |
| Paracentral.L | 0.191 | 0.087 | 0.182 | 0.108 | -0.229 | 0.051 | -0.185 | 0.141 | -0.089 | 0.492 | -0.179 | 0.195 |
| PostCentral.L | 0.189 | 0.087 | 0.172 | 0.116 | -0.283 | 0.022* | -0.189 | 0.138 | -0.272 | 0.039* | -0.218 | 0.125 |
| PostCingulate.L | 0.205 | 0.084 | 0.234 | 0.055 | -0.154 | 0.176 | -0.240 | 0.060 | -0.110 | 0.440 | -0.203 | 0.125 |
| Precentral.L | 0.168 | 0.121 | 0.167 | 0.120 | -0.148 | 0.190 | -0.220 | 0.083 | -0.043 | 0.787 | -0.206 | 0.125 |
| Precuneus.L | 0.209 | 0.084 | 0.191 | 0.092 | -0.181 | 0.113 | -0.192 | 0.138 | -0.231 | 0.060 | -0.289 | 0.053 |
| RosAntCing.L | 0.129 | 0.235 | 0.152 | 0.149 | 0.124 | 0.273 | 0.095 | 0.406 | 0.027 | 0.829 | 0.017 | 0.934 |
| RosMidFront.L | 0.246 | 0.047* | 0.234 | 0.055 | -0.273 | 0.026* | -0.134 | 0.273 | -0.182 | 0.134 | -0.085 | 0.726 |
| SupFront.L | 0.254 | 0.045* | 0.271 | 0.040* | -0.325 | 0.016* | -0.158 | 0.217 | -0.267 | 0.039* | 0.005 | 0.966 |
| Insula.L | 0.199 | 0.087 | 0.289 | 0.037* | -0.311 | 0.016* | -0.265 | 0.048* | -0.320 | 0.019* | -0.144 | 0.329 |
| CaudaAntCing.R | 0.251 | 0.045* | 0.252 | 0.053 | -0.076 | 0.485 | 0.013 | 0.903 | -0.231 | 0.060 | -0.069 | 0.754 |
| CaudMidFront.R | 0.214 | 0.083 | 0.226 | 0.055 | -0.085 | 0.463 | -0.111 | 0.376 | -0.092 | 0.492 | 0.073 | 0.754 |
| LatOrbitoFront.R | 0.204 | 0.084 | 0.326 | 0.037* | -0.304 | 0.016* | -0.223 | 0.083 | -0.156 | 0.213 | -0.078 | 0.750 |
| Paracentral.R | 0.276 | 0.029* | 0.219 | 0.059 | -0.155 | 0.176 | -0.143 | 0.244 | 0.026 | 0.829 | 0.044 | 0.864 |
| PostCentral.R | 0.226 | 0.073 | 0.248 | 0.054 | -0.219 | 0.062 | -0.242 | 0.060 | -0.103 | 0.464 | -0.206 | 0.125 |
| PostCingulate.R | 0.306 | 0.029* | 0.167 | 0.120 | -0.239 | 0.042* | -0.145 | 0.244 | -0.005 | 0.964 | 0.025 | 0.934 |
| Precentral.R | 0.120 | 0.255 | 0.157 | 0.138 | -0.010 | 0.927 | -0.098 | 0.403 | 0.038 | 0.796 | -0.049 | 0.862 |
| Precuneus.R | 0.360 | 0.013* | 0.195 | 0.089 | -0.203 | 0.078 | -0.150 | 0.238 | 0.093 | 0.492 | -0.055 | 0.846 |
| RosAntCing.R | 0.122 | 0.255 | 0.227 | 0.055 | 0.311 | 0.016* | 0.101 | 0.403 | 0.255 | 0.040* | -0.014 | 0.934 |
| RosMidFront.R | 0.273 | 0.029* | 0.213 | 0.064 | -0.263 | 0.031* | -0.101 | 0.403 | -0.279 | 0.039* | -0.013 | 0.934 |
| SupFront.R | 0.195 | 0.087 | 0.231 | 0.055 | -0.203 | 0.078 | -0.023 | 0.857 | -0.238 | 0.058 | 0.036 | 0.876 |
| SupParietal.R | 0.274 | 0.029* | 0.173 | 0.116 | -0.197 | 0.084 | -0.217 | 0.083 | 0.054 | 0.727 | -0.093 | 0.692 |
| SupraMarg.R | 0.281 | 0.029* | 0.252 | 0.053 | -0.329 | 0.016* | -0.302 | 0.048* | -0.226 | 0.062 | -0.305 | 0.053 |
| Insula.R | 0.279 | 0.029* | 0.279 | 0.037* | -0.338 | 0.016* | -0.284 | 0.048* | -0.223 | 0.064 | -0.222 | 0.125 |

**Supplementary table 9: Correlation coefficients and p-values for the relationship between cognitive components and graph theory metrics of a given node within the working memory network constructed using a 2.0 SD thresholding approach.** R=Pearson’s r, p(FDR)=p-value with FDR correction applied across nodes.

2.5 SD Units

| **Working memory network** | | | | | | | | | | | | |
| --- | --- | --- | --- | --- | --- | --- | --- | --- | --- | --- | --- | --- |
| **GT metrics** | **Degree** | | | | **Local Efficiency** | | | | **Clustering coefficient** | | | |
| **Node** | **Reasoning C**  R. p(FDR) | | **Work mem C**  R. p(FDR) | | **Reasoning C**  R. p(FDR) | | **Work mem C**  R. p(FDR) | | **Reasoning C**  R. p(FDR) | | **Work mem C**  R. p(FDR) | |
| Caudate.L^✝^ | 0.231 | 0.041* | 0.312 | 0.032* | -0.321 | 0.029* | -0.297 | 0.042* | -0.369 | 0.005* | -0.217 | 0.118 |
| Pallidum.L | 0.236 | 0.038* | 0.225 | 0.054 | -0.281 | 0.029* | -0.247 | 0.055 | -0.258 | 0.045* | -0.218 | 0.118 |
| Hippocampus.L | 0.188 | 0.083 | 0.222 | 0.054 | -0.273 | 0.029* | -0.269 | 0.046* | -0.377 | 0.005* | -0.321 | 0.028* |
| Caudate.R | 0.244 | 0.036* | 0.294 | 0.032* | -0.273 | 0.029* | -0.266 | 0.046* | -0.309 | 0.028* | -0.262 | 0.073 |
| Pallidum.R^✝^ | 0.266 | 0.029* | 0.333 | 0.032* | -0.237 | 0.051 | -0.283 | 0.044* | -0.202 | 0.105 | -0.241 | 0.093 |
| Hippocampus.R | 0.288 | 0.025* | 0.217 | 0.054 | -0.241 | 0.049* | -0.189 | 0.146 | -0.257 | 0.045* | -0.173 | 0.235 |
| CaudAntCing.L | 0.346 | 0.011* | 0.217 | 0.054 | -0.117 | 0.294 | 0.093 | 0.428 | -0.272 | 0.045* | 0.067 | 0.813 |
| LatOrbitoFront.L | 0.212 | 0.053 | 0.216 | 0.054 | -0.284 | 0.029* | -0.279 | 0.044* | -0.251 | 0.045* | -0.275 | 0.063 |
| Paracentral.L | 0.210 | 0.053 | 0.174 | 0.101 | -0.218 | 0.071 | -0.162 | 0.183 | -0.051 | 0.722 | -0.163 | 0.245 |
| PostCentral.L | 0.212 | 0.053 | 0.199 | 0.068 | -0.266 | 0.029* | -0.181 | 0.153 | -0.264 | 0.045* | -0.199 | 0.162 |
| PostCingulate.L | 0.237 | 0.038* | 0.223 | 0.054 | -0.153 | 0.195 | -0.233 | 0.062 | -0.075 | 0.567 | -0.222 | 0.118 |
| Precentral.L | 0.171 | 0.110 | 0.205 | 0.063 | -0.151 | 0.195 | -0.234 | 0.062 | -0.025 | 0.841 | -0.188 | 0.187 |
| Precuneus.L | 0.210 | 0.053 | 0.178 | 0.096 | -0.160 | 0.181 | -0.173 | 0.171 | -0.214 | 0.083 | -0.274 | 0.063 |
| RosAntCing.L | 0.175 | 0.106 | 0.170 | 0.105 | 0.146 | 0.204 | 0.143 | 0.239 | 0.043 | 0.728 | 0.051 | 0.837 |
| RosMidFront.L | 0.269 | 0.029* | 0.235 | 0.054 | -0.245 | 0.048* | -0.162 | 0.183 | -0.156 | 0.237 | -0.125 | 0.405 |
| SupFront.L | 0.251 | 0.036* | 0.265 | 0.033* | -0.224 | 0.065 | -0.184 | 0.152 | -0.222 | 0.075 | -0.032 | 0.878 |
| Insula.L^✝^ | 0.249 | 0.036* | 0.267 | 0.033* | -0.301 | 0.029* | -0.252 | 0.055 | -0.275 | 0.045* | -0.170 | 0.235 |
| CaudaAntCing.R | 0.242 | 0.036* | 0.276 | 0.033* | -0.053 | 0.642 | 0.027 | 0.799 | -0.254 | 0.045* | -0.042 | 0.837 |
| CaudMidFront.R | 0.222 | 0.047* | 0.185 | 0.086 | -0.052 | 0.642 | -0.030 | 0.799 | -0.097 | 0.459 | 0.101 | 0.555 |
| LatOrbitoFront.R | 0.247 | 0.036* | 0.290 | 0.032* | -0.268 | 0.029* | -0.248 | 0.055 | -0.136 | 0.321 | -0.146 | 0.300 |
| Paracentral.R | 0.294 | 0.025* | 0.248 | 0.049* | -0.136 | 0.226 | -0.120 | 0.315 | 0.046 | 0.728 | 0.028 | 0.878 |
| PostCentral.R | 0.225 | 0.045* | 0.270 | 0.033* | -0.198 | 0.095 | -0.232 | 0.062 | -0.086 | 0.517 | -0.160 | 0.245 |
| PostCingulate.R | 0.287 | 0.025* | 0.215 | 0.054 | -0.204 | 0.087 | -0.166 | 0.183 | -0.014 | 0.897 | 0.010 | 0.936 |
| Precentral.R | 0.106 | 0.327 | 0.194 | 0.074 | 0.031 | 0.773 | -0.127 | 0.295 | 0.120 | 0.386 | -0.043 | 0.837 |
| Precuneus.R^✝^ | 0.353 | 0.011* | 0.228 | 0.054 | -0.164 | 0.176 | -0.142 | 0.239 | 0.100 | 0.459 | -0.013 | 0.936 |
| RosAntCing.R | 0.101 | 0.339 | 0.298 | 0.032* | 0.216 | 0.071 | 0.097 | 0.424 | 0.102 | 0.459 | 0.044 | 0.837 |
| RosMidFront.R | 0.277 | 0.026* | 0.219 | 0.054 | -0.287 | 0.029* | -0.091 | 0.428 | -0.281 | 0.045* | 0.009 | 0.936 |
| SupFront.R | 0.249 | 0.036* | 0.233 | 0.054 | -0.144 | 0.204 | -0.054 | 0.652 | -0.237 | 0.059 | 0.040 | 0.837 |
| SupParietal.R^✝^ | 0.304 | 0.025* | 0.205 | 0.063 | -0.185 | 0.120 | -0.208 | 0.103 | 0.118 | 0.386 | -0.063 | 0.816 |
| SupraMarg.R | 0.294 | 0.025* | 0.242 | 0.053 | -0.319 | 0.029* | -0.299 | 0.042* | -0.231 | 0.064 | -0.336 | 0.028* |
| Insula.R^✝^ | 0.277 | 0.026* | 0.276 | 0.033* | -0.292 | 0.029* | -0.300 | 0.042* | -0.185 | 0.141 | -0.241 | 0.093 |

**Supplementary table 10: Correlation coefficients and p-values for the relationship between cognitive components and graph theory metrics of a given node within the working memory network constructed using a 2.5 SD thresholding approach.** R=Pearson’s r, p(FDR)=p-value with FDR correction applied across nodes.

3.0 SD units

| GT Metrics | Degree | | | | Local Efficiency | | | | Clustering coefficient | | | |
| --- | --- | --- | --- | --- | --- | --- | --- | --- | --- | --- | --- | --- |
| Node | Reasoning  R. p(FDR) | | Working mem.  R. p(FDR) | | Reasoning  R. p(FDR) | | Working mem.  R. p(FDR) | | Reasoning  R. p(FDR) | | Working mem.  R. p(FDR) | |
| Caudate.L | 0.259 | 0.022* | 0.314 | 0.036* | -0.329 | 0.015* | -0.294 | 0.047* | -0.372 | 0.006* | -0.210 | 0.143 |
| Pallidum.L | 0.264 | 0.020* | 0.227 | 0.043* | -0.287 | 0.022* | -0.254 | 0.056 | -0.233 | 0.071 | -0.209 | 0.143 |
| Hippocampus.L | 0.208 | 0.052 | 0.231 | 0.043* | -0.282 | 0.022* | -0.251 | 0.056 | -0.363 | 0.006* | -0.283 | 0.064 |
| Caudate.R | 0.307 | 0.013* | 0.251 | 0.043* | -0.315 | 0.015* | -0.231 | 0.056 | -0.354 | 0.006* | -0.242 | 0.090 |
| Pallidum.R | 0.318 | 0.013* | 0.318 | 0.036* | -0.251 | 0.040* | -0.277 | 0.047* | -0.163 | 0.207 | -0.208 | 0.143 |
| Hippocampus.R | 0.342 | 0.009* | 0.273 | 0.043* | -0.224 | 0.066 | -0.178 | 0.153 | -0.212 | 0.100 | -0.174 | 0.217 |
| CaudAntCing.L | 0.354 | 0.008* | 0.223 | 0.046* | -0.142 | 0.230 | 0.069 | 0.567 | -0.313 | 0.019* | 0.045 | 0.828 |
| LatOrbitoFront.L | 0.234 | 0.033* | 0.234 | 0.043* | -0.310 | 0.015* | -0.288 | 0.047* | -0.288 | 0.028* | -0.268 | 0.077 |
| Paracentral.L | 0.208 | 0.052 | 0.196 | 0.068 | -0.190 | 0.114 | -0.231 | 0.056 | -0.043 | 0.733 | -0.204 | 0.143 |
| PostCentral.L | 0.232 | 0.033* | 0.194 | 0.068 | -0.276 | 0.024* | -0.217 | 0.073 | -0.246 | 0.056 | -0.255 | 0.089 |
| PostCingulate.L | 0.216 | 0.046* | 0.232 | 0.043* | -0.167 | 0.157 | -0.233 | 0.056 | -0.068 | 0.577 | -0.191 | 0.161 |
| Precentral.L | 0.192 | 0.071 | 0.194 | 0.068 | -0.143 | 0.230 | -0.241 | 0.056 | 0.025 | 0.840 | -0.197 | 0.156 |
| Precuneus.L | 0.232 | 0.033* | 0.168 | 0.109 | -0.126 | 0.265 | -0.192 | 0.122 | -0.196 | 0.135 | -0.315 | 0.035* |
| RosAntCing.L | 0.251 | 0.025* | 0.207 | 0.059 | 0.202 | 0.093 | 0.158 | 0.178 | 0.120 | 0.375 | 0.047 | 0.828 |
| RosMidFront.L | 0.306 | 0.013* | 0.229 | 0.043* | -0.245 | 0.045* | -0.171 | 0.160 | -0.153 | 0.226 | -0.140 | 0.330 |
| SupFront.L | 0.277 | 0.017* | 0.239 | 0.043* | -0.209 | 0.083 | -0.132 | 0.262 | -0.172 | 0.183 | 0.010 | 0.965 |
| Insula.L | 0.242 | 0.030* | 0.267 | 0.043* | -0.291 | 0.021* | -0.232 | 0.056 | -0.295 | 0.027* | -0.144 | 0.330 |
| CaudaAntCing.R | 0.326 | 0.012* | 0.191 | 0.070 | -0.134 | 0.243 | 0.009 | 0.932 | -0.253 | 0.051 | -0.055 | 0.813 |
| CaudMidFront.R | 0.256 | 0.023* | 0.195 | 0.068 | -0.019 | 0.859 | -0.037 | 0.749 | -0.084 | 0.507 | 0.092 | 0.622 |
| LatOrbitoFront.R | 0.271 | 0.017* | 0.289 | 0.043* | -0.237 | 0.051 | -0.246 | 0.056 | -0.105 | 0.412 | -0.137 | 0.330 |
| Paracentral.R | 0.293 | 0.014* | 0.245 | 0.043* | -0.139 | 0.233 | -0.169 | 0.160 | 0.076 | 0.542 | -0.071 | 0.779 |
| PostCentral.R | 0.231 | 0.033* | 0.265 | 0.043* | -0.217 | 0.073 | -0.244 | 0.056 | -0.105 | 0.412 | -0.168 | 0.226 |
| PostCingulate.R | 0.272 | 0.017* | 0.256 | 0.043* | -0.167 | 0.157 | -0.169 | 0.160 | -0.009 | 0.932 | 0.032 | 0.857 |
| Precentral.R | 0.151 | 0.156 | 0.211 | 0.056 | 0.038 | 0.741 | -0.162 | 0.174 | 0.177 | 0.177 | -0.062 | 0.785 |
| Precuneus.R | 0.370 | 0.008* | 0.230 | 0.043* | -0.089 | 0.428 | -0.153 | 0.188 | 0.114 | 0.396 | -0.041 | 0.828 |
| RosAntCing.R | 0.147 | 0.161 | 0.248 | 0.043* | 0.348 | 0.015* | 0.062 | 0.592 | 0.159 | 0.210 | -0.009 | 0.965 |
| RosMidFront.R | 0.299 | 0.013* | 0.217 | 0.051 | -0.307 | 0.015* | -0.110 | 0.353 | -0.278 | 0.032* | 0.003 | 0.978 |
| SupFront.R | 0.285 | 0.017* | 0.263 | 0.043* | -0.118 | 0.291 | -0.084 | 0.488 | -0.271 | 0.035* | 0.030 | 0.857 |
| SupParietal.R | 0.279 | 0.017* | 0.241 | 0.043* | -0.176 | 0.146 | -0.248 | 0.056 | 0.087 | 0.505 | -0.064 | 0.785 |
| SupraMarg.R | 0.304 | 0.013* | 0.242 | 0.043* | -0.309 | 0.015* | -0.284 | 0.047* | -0.216 | 0.099 | -0.330 | 0.035* |
| Insula.R | 0.281 | 0.017* | 0.280 | 0.043* | -0.273 | 0.024* | -0.295 | 0.047* | -0.190 | 0.144 | -0.245 | 0.090 |

**Supplementary table 11: Correlation coefficients and p-values for the relationship between cognitive components and graph theory metrics of a given node within the working memory network constructed using a 3.0 SD thresholding approach.** R=Pearson’s r, p(FDR)=p-value with FDR correction applied across nodes.

3.5 SD Units

| GT Metrics | Degree | | | | Local Efficiency | | | | Clustering coefficient | | | |
| --- | --- | --- | --- | --- | --- | --- | --- | --- | --- | --- | --- | --- |
| Node | Reasoning  R. p(FDR) | | Working mem.  R. p(FDR) | | Reasoning  R. p(FDR) | | Working mem.  R. p(FDR) | | Reasoning  R. p(FDR) | | Working mem.  R. p(FDR) | |
| Caudate.L | 0.290 | 0.015* | 0.304 | 0.032* | -0.304 | 0.019* | -0.280 | 0.051 | -0.324 | 0.013* | -0.203 | 0.175 |
| Pallidum.L | 0.248 | 0.026* | 0.223 | 0.048* | -0.258 | 0.037* | -0.240 | 0.058 | -0.207 | 0.122 | -0.195 | 0.175 |
| Hippocampus.L | 0.214 | 0.047* | 0.269 | 0.032* | -0.296 | 0.019* | -0.248 | 0.058 | -0.368 | 0.006* | -0.232 | 0.134 |
| Caudate.R | 0.340 | 0.006* | 0.257 | 0.034* | -0.338 | 0.019* | -0.238 | 0.058 | -0.362 | 0.006* | -0.243 | 0.123 |
| Pallidum.R | 0.348 | 0.006* | 0.298 | 0.032* | -0.297 | 0.019* | -0.258 | 0.058 | -0.210 | 0.122 | -0.183 | 0.205 |
| Hippocampus.R | 0.342 | 0.006* | 0.273 | 0.032* | -0.213 | 0.072 | -0.149 | 0.221 | -0.174 | 0.216 | -0.142 | 0.324 |
| CaudAntCing.L | 0.375 | 0.006* | 0.211 | 0.059 | -0.143 | 0.226 | 0.068 | 0.552 | -0.292 | 0.024* | 0.062 | 0.720 |
| LatOrbitoFront.L | 0.232 | 0.033* | 0.270 | 0.032* | -0.313 | 0.019* | -0.340 | 0.028* | -0.301 | 0.022* | -0.324 | 0.025* |
| Paracentral.L | 0.239 | 0.030* | 0.179 | 0.097 | -0.190 | 0.103 | -0.182 | 0.134 | -0.013 | 0.899 | -0.204 | 0.175 |
| PostCentral.L | 0.220 | 0.042* | 0.176 | 0.100 | -0.251 | 0.037* | -0.206 | 0.101 | -0.233 | 0.078 | -0.244 | 0.123 |
| PostCingulate.L | 0.210 | 0.049* | 0.226 | 0.047* | -0.153 | 0.197 | -0.232 | 0.062 | -0.049 | 0.684 | -0.196 | 0.175 |
| Precentral.L | 0.184 | 0.085 | 0.183 | 0.092 | -0.153 | 0.197 | -0.239 | 0.058 | 0.026 | 0.834 | -0.202 | 0.175 |
| Precuneus.L | 0.256 | 0.023* | 0.184 | 0.092 | -0.127 | 0.262 | -0.197 | 0.117 | -0.149 | 0.242 | -0.292 | 0.049* |
| RosAntCing.L | 0.258 | 0.023* | 0.157 | 0.139 | 0.241 | 0.046* | 0.141 | 0.242 | 0.162 | 0.237 | -0.021 | 0.958 |
| RosMidFront.L | 0.299 | 0.013* | 0.229 | 0.045* | -0.208 | 0.072 | -0.190 | 0.119 | -0.152 | 0.240 | -0.115 | 0.446 |
| SupFront.L | 0.280 | 0.015* | 0.230 | 0.045* | -0.208 | 0.072 | -0.111 | 0.350 | -0.144 | 0.254 | 0.009 | 0.993 |
| Insula.L | 0.257 | 0.023* | 0.245 | 0.040* | -0.307 | 0.019* | -0.222 | 0.075 | -0.322 | 0.013* | -0.165 | 0.240 |
| CaudaAntCing.R | 0.317 | 0.009* | 0.103 | 0.328 | -0.213 | 0.072 | -0.044 | 0.680 | -0.265 | 0.048* | -0.055 | 0.751 |
| CaudMidFront.R | 0.239 | 0.030* | 0.232 | 0.045* | -0.019 | 0.862 | -0.052 | 0.644 | -0.073 | 0.579 | 0.092 | 0.590 |
| LatOrbitoFront.R | 0.286 | 0.015* | 0.296 | 0.032* | -0.254 | 0.037* | -0.253 | 0.058 | -0.132 | 0.294 | -0.145 | 0.323 |
| Paracentral.R | 0.270 | 0.018* | 0.203 | 0.068 | -0.127 | 0.262 | -0.164 | 0.184 | 0.092 | 0.472 | -0.129 | 0.378 |
| PostCentral.R | 0.232 | 0.033* | 0.238 | 0.043* | -0.226 | 0.059 | -0.238 | 0.058 | -0.095 | 0.472 | -0.174 | 0.217 |
| PostCingulate.R | 0.240 | 0.030* | 0.282 | 0.032* | -0.231 | 0.056 | -0.193 | 0.118 | -0.071 | 0.579 | -0.062 | 0.720 |
| Precentral.R | 0.171 | 0.106 | 0.215 | 0.056 | -0.018 | 0.862 | -0.132 | 0.260 | 0.165 | 0.237 | -0.004 | 0.993 |
| Precuneus.R | 0.347 | 0.006* | 0.258 | 0.034* | -0.056 | 0.638 | -0.157 | 0.198 | 0.123 | 0.325 | -0.019 | 0.958 |
| RosAntCing.R | 0.133 | 0.208 | 0.266 | 0.032* | 0.317 | 0.019* | -0.074 | 0.538 | 0.061 | 0.624 | -0.063 | 0.720 |
| RosMidFront.R | 0.319 | 0.009* | 0.191 | 0.085 | -0.276 | 0.027* | -0.091 | 0.447 | -0.253 | 0.054 | 0.001 | 0.993 |
| SupFront.R | 0.277 | 0.015* | 0.282 | 0.032* | -0.099 | 0.384 | -0.136 | 0.252 | -0.251 | 0.054 | 0.018 | 0.958 |
| SupParietal.R | 0.287 | 0.015* | 0.244 | 0.040* | -0.138 | 0.237 | -0.279 | 0.051 | 0.154 | 0.240 | -0.069 | 0.720 |
| SupraMarg.R | 0.304 | 0.012* | 0.251 | 0.038* | -0.283 | 0.024* | -0.311 | 0.039* | -0.157 | 0.240 | -0.335 | 0.025* |
| Insula.R | 0.281 | 0.015* | 0.289 | 0.032* | -0.266 | 0.032* | -0.274 | 0.051 | -0.202 | 0.128 | -0.180 | 0.205 |

**Supplementary table 12: Correlation coefficients and p-values for the relationship between cognitive components and graph theory metrics of a given node within the working memory network constructed using a 3.5 SD thresholding approach.** R=Pearson’s r, p(FDR)=p-value with FDR correction applied across nodes.

4.0 SD Units

| GT Metrics | Degree | | | | Local Efficiency | | | | Clustering coefficient | | | |
| --- | --- | --- | --- | --- | --- | --- | --- | --- | --- | --- | --- | --- |
| Node | Reasoning  R. p(FDR) | | Working mem.  R. p(FDR) | | Reasoning  R. p(FDR) | | Working mem.  R. p(FDR) | | Reasoning  R. p(FDR) | | Working mem.  R. p(FDR) | |
| Caudate.L | 0.286 | 0.013* | 0.315 | 0.023* | -0.306 | 0.016* | -0.302 | 0.035* | -0.331 | 0.010* | -0.244 | 0.075 |
| Pallidum.L | 0.210 | 0.049* | 0.252 | 0.034* | -0.241 | 0.047* | -0.292 | 0.037* | -0.228 | 0.086 | -0.249 | 0.075 |
| Hippocampus.L | 0.242 | 0.030* | 0.262 | 0.033* | -0.310 | 0.016* | -0.233 | 0.065 | -0.383 | 0.004* | -0.248 | 0.075 |
| Caudate.R | 0.351 | 0.008* | 0.257 | 0.034* | -0.345 | 0.012* | -0.227 | 0.069 | -0.367 | 0.004* | -0.247 | 0.075 |
| Pallidum.R | 0.327 | 0.008* | 0.308 | 0.023* | -0.291 | 0.019* | -0.250 | 0.055 | -0.221 | 0.089 | -0.151 | 0.311 |
| Hippocampus.R | 0.330 | 0.008* | 0.280 | 0.023* | -0.237 | 0.047* | -0.170 | 0.137 | -0.225 | 0.086 | -0.168 | 0.240 |
| CaudAntCing.L | 0.370 | 0.008* | 0.178 | 0.098 | -0.195 | 0.091 | 0.013 | 0.900 | -0.365 | 0.004* | -0.014 | 0.893 |
| LatOrbitoFront.L | 0.214 | 0.048* | 0.283 | 0.023* | -0.264 | 0.038* | -0.325 | 0.025* | -0.253 | 0.066 | -0.280 | 0.075 |
| Paracentral.L | 0.227 | 0.038* | 0.177 | 0.098 | -0.171 | 0.146 | -0.215 | 0.077 | -0.003 | 0.980 | -0.204 | 0.143 |
| PostCentral.L | 0.211 | 0.049* | 0.180 | 0.098 | -0.225 | 0.056 | -0.222 | 0.069 | -0.207 | 0.114 | -0.261 | 0.075 |
| PostCingulate.L | 0.226 | 0.038* | 0.211 | 0.061 | -0.127 | 0.271 | -0.205 | 0.086 | -0.022 | 0.891 | -0.190 | 0.165 |
| Precentral.L | 0.147 | 0.162 | 0.159 | 0.134 | -0.115 | 0.307 | -0.244 | 0.055 | -0.015 | 0.920 | -0.238 | 0.076 |
| Precuneus.L | 0.257 | 0.022* | 0.190 | 0.087 | -0.137 | 0.250 | -0.185 | 0.120 | -0.161 | 0.217 | -0.266 | 0.075 |
| RosAntCing.L | 0.281 | 0.013* | 0.183 | 0.095 | 0.216 | 0.063 | 0.153 | 0.179 | 0.108 | 0.428 | 0.027 | 0.869 |
| RosMidFront.L | 0.321 | 0.008* | 0.241 | 0.036* | -0.235 | 0.047* | -0.224 | 0.069 | -0.145 | 0.259 | -0.117 | 0.435 |
| SupFront.L | 0.281 | 0.013* | 0.239 | 0.036* | -0.223 | 0.056 | -0.183 | 0.120 | -0.170 | 0.215 | -0.017 | 0.893 |
| Insula.L | 0.283 | 0.013* | 0.255 | 0.034* | -0.317 | 0.016* | -0.256 | 0.055 | -0.303 | 0.017* | -0.205 | 0.143 |
| CaudaAntCing.R | 0.276 | 0.014* | 0.118 | 0.265 | -0.298 | 0.017* | -0.077 | 0.481 | -0.308 | 0.017* | -0.084 | 0.591 |
| CaudMidFront.R | 0.230 | 0.038* | 0.243 | 0.036* | -0.043 | 0.708 | -0.082 | 0.465 | -0.038 | 0.795 | 0.082 | 0.591 |
| LatOrbitoFront.R | 0.261 | 0.020* | 0.287 | 0.023* | -0.237 | 0.047* | -0.205 | 0.086 | -0.164 | 0.215 | -0.147 | 0.312 |
| Paracentral.R | 0.284 | 0.013* | 0.195 | 0.081 | -0.133 | 0.257 | -0.178 | 0.125 | 0.105 | 0.428 | -0.139 | 0.342 |
| PostCentral.R | 0.252 | 0.024* | 0.239 | 0.036* | -0.210 | 0.069 | -0.255 | 0.055 | -0.075 | 0.547 | -0.192 | 0.165 |
| PostCingulate.R | 0.229 | 0.038* | 0.289 | 0.023* | -0.244 | 0.047* | -0.243 | 0.055 | -0.099 | 0.451 | -0.098 | 0.547 |
| Precentral.R | 0.159 | 0.135 | 0.207 | 0.065 | -0.011 | 0.921 | -0.127 | 0.265 | 0.170 | 0.215 | 0.025 | 0.869 |
| Precuneus.R | 0.334 | 0.008* | 0.247 | 0.036* | -0.082 | 0.465 | -0.176 | 0.125 | 0.106 | 0.428 | -0.057 | 0.734 |
| RosAntCing.R | 0.197 | 0.063 | 0.284 | 0.023* | 0.421 | 0.001* | -0.126 | 0.265 | 0.076 | 0.547 | -0.066 | 0.686 |
| RosMidFront.R | 0.337 | 0.008* | 0.222 | 0.049* | -0.312 | 0.016* | -0.092 | 0.425 | -0.238 | 0.086 | 0.037 | 0.863 |
| SupFront.R | 0.302 | 0.012* | 0.283 | 0.023* | -0.146 | 0.221 | -0.186 | 0.120 | -0.232 | 0.086 | 0.028 | 0.869 |
| SupParietal.R | 0.291 | 0.013* | 0.235 | 0.037* | -0.125 | 0.271 | -0.259 | 0.055 | 0.153 | 0.235 | -0.089 | 0.590 |
| SupraMarg.R | 0.289 | 0.013* | 0.272 | 0.027* | -0.239 | 0.047* | -0.340 | 0.025* | -0.086 | 0.516 | -0.312 | 0.075 |
| Insula.R | 0.304 | 0.012* | 0.307 | 0.023* | -0.259 | 0.040* | -0.258 | 0.055 | -0.164 | 0.215 | -0.118 | 0.435 |

**Supplementary table 13: Correlation coefficients and p-values for the relationship between cognitive components and graph theory metrics of a given node within the working memory network constructed using a 4.0 SD thresholding approach.** R=Pearson’s r, p(FDR)=p-value with FDR correction applied across nodes.

4.5 SD Units

| GT Metrics | Degree | | | | Local Efficiency | | | | Clustering coefficient | | | |
| --- | --- | --- | --- | --- | --- | --- | --- | --- | --- | --- | --- | --- |
| Node | Reasoning  R. p(FDR) | | Working mem.  R. p(FDR) | | Reasoning  R. p(FDR) | | Working mem,  R. p(FDR) | | Reasoning  R. p(FDR) | | Working mem.  R. p(FDR) | |
| Caudate.L | 0.294 | 0.011* | 0.303 | 0.015* | -0.337 | 0.011* | -0.305 | 0.027* | -0.365 | 0.003* | -0.246 | 0.071 |
| Pallidum.L | 0.222 | 0.041* | 0.247 | 0.029* | -0.246 | 0.033* | -0.286 | 0.035* | -0.213 | 0.120 | -0.249 | 0.071 |
| Hippocampus.L | 0.261 | 0.018* | 0.257 | 0.027* | -0.325 | 0.012* | -0.216 | 0.067 | -0.385 | 0.003* | -0.226 | 0.104 |
| Caudate.R | 0.356 | 0.007* | 0.269 | 0.027* | -0.369 | 0.005* | -0.256 | 0.039* | -0.382 | 0.003* | -0.261 | 0.071 |
| Pallidum.R | 0.314 | 0.009* | 0.307 | 0.015* | -0.300 | 0.017* | -0.247 | 0.045* | -0.249 | 0.058 | -0.140 | 0.358 |
| Hippocampus.R | 0.314 | 0.009* | 0.268 | 0.027* | -0.272 | 0.024* | -0.228 | 0.060 | -0.268 | 0.043* | -0.218 | 0.104 |
| CaudAntCing.L | 0.363 | 0.007* | 0.169 | 0.114 | -0.269 | 0.025* | -0.013 | 0.899 | -0.371 | 0.003* | -0.037 | 0.840 |
| LatOrbitoFront.L | 0.228 | 0.037* | 0.309 | 0.015* | -0.279 | 0.022* | -0.350 | 0.015* | -0.263 | 0.043* | -0.324 | 0.040* |
| Paracentral.L | 0.214 | 0.049* | 0.180 | 0.099* | -0.180 | 0.120 | -0.209 | 0.070 | -0.035 | 0.791 | -0.189 | 0.157 |
| PostCentral.L | 0.191 | 0.073 | 0.170 | 0.114 | -0.214 | 0.067 | -0.228 | 0.060 | -0.190 | 0.154 | -0.260 | 0.071 |
| PostCingulate.L | 0.209 | 0.053 | 0.227 | 0.042* | -0.112 | 0.319 | -0.222 | 0.061 | -0.024 | 0.824 | -0.198 | 0.151 |
| Precentral.L | 0.155 | 0.145 | 0.184 | 0.098 | -0.134 | 0.243 | -0.256 | 0.039* | -0.027 | 0.821 | -0.219 | 0.104 |
| Precuneus.L | 0.258 | 0.019* | 0.180 | 0.099 | -0.157 | 0.183 | -0.203 | 0.074 | -0.166 | 0.206 | -0.278 | 0.071 |
| RosAntCing.L | 0.321 | 0.009* | 0.131 | 0.213 | 0.250 | 0.033* | 0.153 | 0.181 | 0.128 | 0.321 | 0.017 | 0.891 |
| RosMidFront.L | 0.324 | 0.009* | 0.259 | 0.027* | -0.257 | 0.030* | -0.266 | 0.039* | -0.119 | 0.346 | -0.122 | 0.426 |
| SupFront.L | 0.275 | 0.014* | 0.263 | 0.027* | -0.218 | 0.063 | -0.223 | 0.061 | -0.134 | 0.316 | -0.026 | 0.857 |
| Insula.L | 0.277 | 0.014* | 0.266 | 0.027* | -0.316 | 0.013* | -0.301 | 0.027* | -0.302 | 0.018* | -0.249 | 0.071 |
| CaudaAntCing.R | 0.331 | 0.009* | 0.150 | 0.159 | -0.298 | 0.017* | -0.106 | 0.347 | -0.320 | 0.011* | -0.101 | 0.555 |
| CaudMidFront.R | 0.205 | 0.055 | 0.256 | 0.027* | -0.020 | 0.880 | -0.099 | 0.369 | -0.054 | 0.724 | 0.055 | 0.839 |
| LatOrbitoFront.R | 0.262 | 0.018* | 0.324 | 0.015* | -0.207 | 0.071 | -0.188 | 0.095 | -0.134 | 0.316 | -0.122 | 0.426 |
| Paracentral.R | 0.300 | 0.011* | 0.215 | 0.054 | -0.129 | 0.252 | -0.206 | 0.072 | 0.127 | 0.321 | -0.180 | 0.177 |
| PostCentral.R | 0.278 | 0.014* | 0.252 | 0.028* | -0.209 | 0.071 | -0.271 | 0.039* | -0.036 | 0.791 | -0.194 | 0.151 |
| PostCingulate.R | 0.244 | 0.025* | 0.261 | 0.027* | -0.261 | 0.028* | -0.236 | 0.056 | -0.083 | 0.535 | -0.048 | 0.839 |
| Precentral.R | 0.145 | 0.167 | 0.209 | 0.059 | -0.012 | 0.906 | -0.119 | 0.296 | 0.153 | 0.248 | 0.051 | 0.839 |
| Precuneus.R | 0.303 | 0.011* | 0.247 | 0.029* | -0.056 | 0.634 | -0.212 | 0.069 | 0.171 | 0.206 | -0.015 | 0.891 |
| RosAntCing.R | 0.263 | 0.018* | 0.304 | 0.015* | 0.380 | 0.005* | -0.086 | 0.428 | 0.040 | 0.791 | -0.030 | 0.857 |
| RosMidFront.R | 0.348 | 0.007* | 0.233 | 0.039* | -0.290 | 0.020* | -0.125 | 0.279 | -0.209 | 0.120 | 0.040 | 0.840 |
| SupFront.R | 0.295 | 0.011* | 0.303 | 0.015* | -0.150 | 0.192 | -0.193 | 0.088 | -0.208 | 0.120 | 0.036 | 0.840 |
| SupParietal.R | 0.292 | 0.011* | 0.232 | 0.039* | -0.150 | 0.192 | -0.280 | 0.035* | 0.168 | 0.206 | -0.067 | 0.777 |
| SupraMarg.R | 0.289 | 0.011* | 0.276 | 0.027* | -0.246 | 0.033* | -0.338 | 0.015* | -0.102 | 0.429 | -0.310 | 0.040* |
| Insula.R | 0.281 | 0.014* | 0.320 | 0.015* | -0.281 | 0.022* | -0.261 | 0.039* | -0.191 | 0.154 | -0.085 | 0.648 |

**Supplementary table 14: Correlation coefficients and p-values for the relationship between cognitive components and graph theory metrics of a given node within the working memory network constructed using a 4.5 SD thresholding approach.** R=Pearson’s r, p(FDR)=p-value with FDR correction applied across nodes.

5.0 SD Units

| GT Metrics | Degree | | | | Local Efficiency | | | | Clustering coefficient | | | |
| --- | --- | --- | --- | --- | --- | --- | --- | --- | --- | --- | --- | --- |
| Node | Reasoning  R. p(FDR) | | Working mem.  R. p(FDR) | | Reasoning  R. p(FDR) | | Working mem.  R. p(FDR) | | Reasoning  R. p(FDR) | | Working mem.  R. p(FDR) | |
| Caudate.L | 0.301 | 0.015* | 0.307 | 0.023* | -0.335 | 0.014* | -0.304 | 0.017* | -0.354 | 0.004* | -0.228 | 0.091 |
| Pallidum.L | 0.190 | 0.077 | 0.261 | 0.025* | -0.208 | 0.077 | -0.311 | 0.016* | -0.202 | 0.126 | -0.282 | 0.050 |
| Hippocampus.L | 0.277 | 0.016* | 0.239 | 0.035* | -0.321 | 0.014* | -0.200 | 0.075 | -0.354 | 0.004* | -0.225 | 0.091 |
| Caudate.R | 0.345 | 0.008* | 0.282 | 0.023* | -0.355 | 0.014* | -0.252 | 0.046* | -0.365 | 0.004* | -0.238 | 0.091 |
| Pallidum.R | 0.302 | 0.015* | 0.271 | 0.023* | -0.294 | 0.020* | -0.247 | 0.046* | -0.261 | 0.042* | -0.169 | 0.208 |
| Hippocampus.R | 0.287 | 0.015* | 0.275 | 0.023* | -0.293 | 0.020* | -0.240 | 0.050 | -0.289 | 0.027* | -0.236 | 0.091 |
| CaudAntCing.L | 0.355 | 0.008* | 0.167 | 0.122 | -0.313 | 0.015* | 0.009 | 0.930 | -0.410 | 0.002* | -0.036 | 0.864 |
| LatOrbitoFront.L | 0.252 | 0.021* | 0.338 | 0.023* | -0.284 | 0.021* | -0.343 | 0.016* | -0.262 | 0.042* | -0.288 | 0.050 |
| Paracentral.L | 0.207 | 0.059 | 0.178 | 0.102 | -0.165 | 0.163 | -0.210 | 0.065 | -0.006 | 0.981 | -0.220 | 0.091 |
| PostCentral.L | 0.194 | 0.073 | 0.166 | 0.122 | -0.208 | 0.077 | -0.227 | 0.056 | -0.175 | 0.196 | -0.263 | 0.058 |
| PostCingulate.L | 0.219 | 0.046* | 0.208 | 0.062 | -0.128 | 0.265 | -0.215 | 0.062 | -0.047 | 0.785 | -0.201 | 0.126 |
| Precentral.L | 0.150 | 0.155 | 0.181 | 0.101 | -0.110 | 0.338 | -0.252 | 0.046* | 0.024 | 0.907 | -0.220 | 0.091 |
| Precuneus.L | 0.263 | 0.018* | 0.188 | 0.092 | -0.138 | 0.236 | -0.226 | 0.056 | -0.124 | 0.389 | -0.269 | 0.058 |
| RosAntCing.L | 0.313 | 0.015* | 0.109 | 0.302 | 0.227 | 0.071 | 0.190 | 0.089 | 0.078 | 0.592 | 0.024 | 0.864 |
| RosMidFront.L | 0.282 | 0.015* | 0.252 | 0.027* | -0.179 | 0.129 | -0.310 | 0.016* | 0.003 | 0.981 | -0.114 | 0.456 |
| SupFront.L | 0.279 | 0.016* | 0.272 | 0.023* | -0.213 | 0.077 | -0.222 | 0.058 | -0.068 | 0.642 | -0.002 | 0.982 |
| Insula.L | 0.255 | 0.021* | 0.265 | 0.024* | -0.289 | 0.020* | -0.315 | 0.016* | -0.290 | 0.027* | -0.292 | 0.050 |
| CaudaAntCing.R | 0.345 | 0.008* | 0.162 | 0.126 | -0.274 | 0.023* | -0.157 | 0.161 | -0.261 | 0.042* | -0.124 | 0.412 |
| CaudMidFront.R | 0.182 | 0.089 | 0.227 | 0.043* | -0.088 | 0.430 | -0.130 | 0.242 | -0.112 | 0.412 | 0.022 | 0.864 |
| LatOrbitoFront.R | 0.253 | 0.021* | 0.299 | 0.023* | -0.188 | 0.112 | -0.208 | 0.066 | -0.111 | 0.412 | -0.160 | 0.233 |
| Paracentral.R | 0.294 | 0.015* | 0.220 | 0.050 | -0.144 | 0.222 | -0.217 | 0.061 | 0.118 | 0.406 | -0.187 | 0.154 |
| PostCentral.R | 0.284 | 0.015* | 0.273 | 0.023* | -0.211 | 0.077 | -0.293 | 0.020* | -0.014 | 0.952 | -0.199 | 0.126 |
| PostCingulate.R | 0.195 | 0.073 | 0.302 | 0.023* | -0.212 | 0.077 | -0.235 | 0.053 | -0.124 | 0.389 | -0.092 | 0.565 |
| Precentral.R | 0.150 | 0.155 | 0.187 | 0.092 | -0.031 | 0.772 | -0.073 | 0.508 | 0.158 | 0.254 | 0.093 | 0.565 |
| Precuneus.R | 0.271 | 0.016* | 0.256 | 0.025* | -0.058 | 0.602 | -0.229 | 0.056 | 0.127 | 0.389 | -0.057 | 0.761 |
| RosAntCing.R | 0.262 | 0.018* | 0.288 | 0.023* | 0.329 | 0.014* | -0.117 | 0.287 | 0.040 | 0.806 | -0.067 | 0.706 |
| RosMidFront.R | 0.317 | 0.015* | 0.258 | 0.025* | -0.255 | 0.036* | -0.145 | 0.194 | -0.213 | 0.121 | 0.038 | 0.864 |
| SupFront.R | 0.297 | 0.015* | 0.292 | 0.023* | -0.144 | 0.222 | -0.179 | 0.108 | -0.208 | 0.121 | 0.028 | 0.864 |
| SupParietal.R | 0.263 | 0.018* | 0.238 | 0.035* | -0.093 | 0.418 | -0.266 | 0.041* | 0.208 | 0.121 | -0.025 | 0.864 |
| SupraMarg.R | 0.273 | 0.016* | 0.269 | 0.023* | -0.218 | 0.077 | -0.332 | 0.016* | -0.093 | 0.506 | -0.323 | 0.050 |
| Insula.R | 0.283 | 0.015* | 0.321 | 0.023* | -0.276 | 0.023* | -0.246 | 0.046* | -0.179 | 0.193 | -0.068 | 0.706 |

**Supplementary table 15: Correlation coefficients and p-values for the relationship between cognitive components and graph theory metrics of a given node within the working memory network constructed using a 5.0 SD thresholding approach.** R=Pearson’s r, p(FDR)=p-value with FDR correction applied across nodes.

**Reasoning network correlations to cognitive components across all structural connectome threshold iterations (see supplementary figure 10 for graphical illustration of results)**

**0.5 SD Units**

| GT Metrics | Degree | | | | Local Eff | | | | Clustering Coeff | | | |
| --- | --- | --- | --- | --- | --- | --- | --- | --- | --- | --- | --- | --- |
| Node | Reasoning  R. p(FDR) | | Working mem.  R. p(FDR) | | Reasoning  R. p(FDR) | | Working mem.  R. p(FDR) | | Reasoning  R. p(FDR) | | Working mem.  R. p(FDR) | |
| CaudMidFront.L | 0.015 | 0.884 | 0.148 | 0.459 | -0.067 | 0.704 | 0.101 | 0.835 | -0.080 | 0.765 | -0.054 | 0.899 |
| Cuneus.L | 0.060 | 0.647 | -0.098 | 0.555 | -0.123 | 0.445 | -0.036 | 0.952 | -0.126 | 0.639 | -0.023 | 0.899 |
| Fusiform.L | 0.149 | 0.389 | 0.054 | 0.757 | -0.077 | 0.681 | -0.077 | 0.866 | -0.068 | 0.765 | -0.062 | 0.899 |
| InfParietal.L | 0.263 | 0.097 | 0.046 | 0.781 | -0.278 | 0.067 | -0.032 | 0.952 | -0.240 | 0.154 | -0.190 | 0.381 |
| InfTemporal.L | 0.187 | 0.244 | 0.003 | 0.979 | -0.170 | 0.345 | -0.031 | 0.952 | -0.078 | 0.765 | 0.005 | 0.962 |
| LatOcc.L | 0.174 | 0.283 | 0.155 | 0.459 | -0.137 | 0.415 | -0.017 | 0.970 | -0.184 | 0.440 | -0.038 | 0.899 |
| LatOrbFront.L | 0.135 | 0.389 | 0.026 | 0.839 | -0.292 | 0.067 | -0.101 | 0.835 | NaN | NaN | NaN | NaN |
| ParsOperc.L | -0.099 | 0.503 | -0.056 | 0.757 | -0.193 | 0.282 | -0.005 | 0.972 | -0.314 | 0.051 | -0.100 | 0.815 |
| Pericalcarine.L | 0.133 | 0.389 | 0.032 | 0.826 | -0.145 | 0.412 | -0.112 | 0.835 | -0.018 | 0.897 | 0.118 | 0.723 |
| Precentral.L | -0.085 | 0.548 | 0.192 | 0.388 | -0.081 | 0.681 | -0.052 | 0.952 | -0.104 | 0.765 | -0.156 | 0.506 |
| RosMidFront.L | 0.107 | 0.480 | 0.105 | 0.553 | -0.025 | 0.884 | 0.150 | 0.835 | NaN | NaN | NaN | NaN |
| SupFront.L | 0.040 | 0.767 | 0.127 | 0.484 | 0.015 | 0.923 | 0.143 | 0.835 | 0.045 | 0.889 | 0.252 | 0.169 |
| SupParietal.L | 0.141 | 0.389 | 0.191 | 0.388 | -0.276 | 0.067 | -0.104 | 0.835 | -0.245 | 0.154 | -0.260 | 0.169 |
| CaudMidFront.R | -0.026 | 0.837 | 0.183 | 0.388 | 0.076 | 0.681 | -0.015 | 0.970 | NaN | NaN | NaN | NaN |
| Cuneus.R | 0.197 | 0.222 | 0.178 | 0.388 | -0.151 | 0.412 | -0.036 | 0.952 | 0.093 | 0.765 | 0.040 | 0.899 |
| Fusiform.R | 0.225 | 0.133 | 0.068 | 0.710 | -0.183 | 0.302 | -0.100 | 0.835 | -0.070 | 0.765 | -0.170 | 0.464 |
| InfParietal.R | 0.387 | 0.004* | 0.096 | 0.555 | -0.219 | 0.234 | 0.004 | 0.972 | -0.026 | 0.889 | -0.020 | 0.899 |
| InfTemporal.R | 0.252 | 0.100 | 0.150 | 0.459 | -0.119 | 0.445 | -0.081 | 0.866 | -0.014 | 0.897 | 0.034 | 0.899 |
| LateralOcc.R | 0.132 | 0.389 | 0.187 | 0.388 | -0.063 | 0.704 | -0.111 | 0.835 | 0.042 | 0.889 | -0.094 | 0.815 |
| Lingual.R | 0.121 | 0.436 | 0.038 | 0.812 | -0.143 | 0.412 | -0.052 | 0.952 | -0.172 | 0.448 | -0.206 | 0.362 |
| ParsOperc.R | 0.077 | 0.578 | 0.126 | 0.484 | -0.031 | 0.872 | -0.014 | 0.970 | -0.133 | 0.639 | 0.029 | 0.899 |
| Paricalcarine.R | 0.232 | 0.133 | 0.117 | 0.493 | -0.196 | 0.282 | -0.098 | 0.835 | -0.136 | 0.639 | -0.042 | 0.899 |
| Precentral.R | 0.072 | 0.587 | 0.303 | 0.086 | -0.048 | 0.765 | -0.174 | 0.835 | 0.031 | 0.889 | -0.019 | 0.899 |
| RosMidFront.R | 0.087 | 0.548 | 0.086 | 0.603 | 0.060 | 0.704 | 0.068 | 0.905 | 0.088 | 0.765 | 0.145 | 0.525 |
| SupFront.R | 0.106 | 0.480 | 0.133 | 0.484 | 0.010 | 0.926 | 0.109 | 0.835 | NaN | NaN | NaN | NaN |
| SupParietal.R | 0.335 | 0.014* | 0.123 | 0.484 | -0.125 | 0.445 | 0.084 | 0.866 | 0.029 | 0.889 | 0.033 | 0.899 |

**Supplementary table 16: Correlation coefficients and p-values for the relationship between cognitive components and graph theory metrics of a given node within the reasoning network constructed using a 0.5 SD thresholding approach.** R=Pearson’s r, p(FDR)=p-value with FDR correction applied across nodes.

1. SD Units

| GT Metrics | Degree | | | | Local Eff | | | | Clustering Coeff | | | |
| --- | --- | --- | --- | --- | --- | --- | --- | --- | --- | --- | --- | --- |
| Node | Reasoning  R. p(FDR) | | Working mem.  R. p(FDR) | | Reasoning  R. p(FDR) | | Working mem.  R. p(FDR) | | Reasoning  R. p(FDR) | | Working mem.  R. p(FDR) | |
| CaudMidFront.L | 0.005 | 0.959 | 0.044 | 0.768 | 0.013 | 0.971 | 0.119 | 0.727 | -0.105 | 0.566 | 0.007 | 0.949 |
| Cuneus.L | 0.161 | 0.181 | -0.050 | 0.751 | -0.108 | 0.395 | 0.032 | 0.849 | -0.154 | 0.443 | -0.046 | 0.787 |
| Fusiform.L | 0.176 | 0.162 | 0.027 | 0.802 | -0.132 | 0.338 | -0.092 | 0.765 | -0.143 | 0.451 | -0.170 | 0.478 |
| InfParietal.L | 0.321 | 0.009* | 0.080 | 0.599 | -0.334 | 0.024* | -0.070 | 0.829 | -0.314 | 0.035* | -0.166 | 0.478 |
| InfTemporal.L | 0.305 | 0.013* | -0.034 | 0.775 | -0.299 | 0.024* | -0.034 | 0.849 | -0.277 | 0.047* | -0.179 | 0.478 |
| LatOcc.L | 0.259 | 0.033* | 0.106 | 0.588 | -0.277 | 0.028* | -0.054 | 0.849 | -0.308 | 0.035* | -0.056 | 0.787 |
| LatOrbFront.L | 0.173 | 0.162 | 0.154 | 0.373 | -0.303 | 0.024* | -0.175 | 0.727 | -0.042 | 0.806 | -0.020 | 0.920 |
| ParsOperc.L | -0.035 | 0.835 | 0.039 | 0.771 | -0.186 | 0.179 | -0.108 | 0.727 | -0.288 | 0.045* | -0.109 | 0.758 |
| Pericalcarine.L | 0.188 | 0.134 | 0.055 | 0.745 | -0.186 | 0.179 | -0.029 | 0.849 | -0.119 | 0.547 | 0.059 | 0.787 |
| Precentral.L | 0.016 | 0.915 | 0.126 | 0.541 | -0.019 | 0.971 | -0.109 | 0.727 | -0.043 | 0.806 | -0.176 | 0.478 |
| RosMidFront.L | 0.255 | 0.033* | 0.083 | 0.599 | 0.009 | 0.971 | 0.168 | 0.727 | 0.118 | 0.547 | 0.061 | 0.787 |
| SupFront.L | 0.024 | 0.885 | 0.200 | 0.214 | 0.118 | 0.373 | 0.094 | 0.765 | 0.070 | 0.745 | 0.191 | 0.478 |
| SupParietal.L | 0.327 | 0.009* | 0.243 | 0.214 | -0.309 | 0.024* | -0.144 | 0.727 | -0.218 | 0.182 | -0.266 | 0.260 |
| CaudMidFront.R | 0.067 | 0.621 | 0.199 | 0.214 | 0.116 | 0.373 | -0.048 | 0.849 | -0.046 | 0.806 | 0.042 | 0.787 |
| Cuneus.R | 0.296 | 0.016* | 0.204 | 0.214 | -0.179 | 0.190 | 0.007 | 0.950 | 0.034 | 0.806 | 0.092 | 0.787 |
| Fusiform.R | 0.247 | 0.036* | 0.089 | 0.599 | -0.232 | 0.084 | -0.136 | 0.727 | -0.174 | 0.346 | -0.128 | 0.701 |
| InfParietal.R | 0.404 | 0.001* | 0.078 | 0.599 | -0.210 | 0.128 | -0.039 | 0.849 | -0.011 | 0.918 | -0.059 | 0.787 |
| InfTemporal.R | 0.267 | 0.029* | 0.121 | 0.541 | -0.146 | 0.305 | -0.133 | 0.727 | -0.030 | 0.806 | -0.045 | 0.787 |
| LateralOcc.R | 0.289 | 0.017* | 0.183 | 0.263 | -0.136 | 0.338 | -0.143 | 0.727 | 0.032 | 0.806 | -0.043 | 0.787 |
| Lingual.R | 0.246 | 0.036* | 0.094 | 0.599 | -0.284 | 0.026* | -0.078 | 0.799 | -0.191 | 0.286 | -0.112 | 0.758 |
| ParsOperc.R | 0.119 | 0.338 | 0.206 | 0.214 | -0.011 | 0.971 | 0.008 | 0.950 | -0.108 | 0.566 | 0.059 | 0.787 |
| Paricalcarine.R | 0.322 | 0.009* | 0.217 | 0.214 | -0.284 | 0.026* | -0.108 | 0.727 | -0.141 | 0.451 | 0.054 | 0.787 |
| Precentral.R | 0.161 | 0.181 | 0.223 | 0.214 | -0.002 | 0.986 | -0.142 | 0.727 | 0.064 | 0.755 | -0.016 | 0.920 |
| RosMidFront.R | 0.086 | 0.511 | 0.085 | 0.599 | 0.154 | 0.287 | 0.083 | 0.797 | 0.076 | 0.733 | 0.149 | 0.555 |
| SupFront.R | 0.153 | 0.200 | 0.116 | 0.543 | 0.091 | 0.480 | 0.060 | 0.849 | NaN | NaN | NaN | NaN |
| SupParietal.R | 0.413 | 0.001* | 0.173 | 0.288 | -0.128 | 0.340 | -0.042 | 0.849 | 0.081 | 0.733 | -0.054 | 0.787 |

**Supplementary table 17: Correlation coefficients and p-values for the relationship between cognitive components and graph theory metrics of a given node within the reasoning network constructed using a 1.0 SD thresholding approach.** R=Pearson’s r, p(FDR)=p-value with FDR correction applied across nodes.

1.5 SD Units

| **GT Metrics** | Degree | | | | Local Eff | | | | Clustering Coeff | | | |
| --- | --- | --- | --- | --- | --- | --- | --- | --- | --- | --- | --- | --- |
| Node | Reasoning  R. p(FDR) | | Working mem.  R. P(FDR) | | Reasoning  R. p(FDR) | | Working mem.  R. p(FDR) | | Reasoning  R. p(FDR) | | Working mem.  R. p(FDR) | |
| CaudMidFron.L | 0.137 | 0.200 | -0.015 | 0.884 | -0.020 | 0.854 | 0.148 | 0.458 | -0.082 | 0.648 | -0.033 | 0.899 |
| Cuneus.L | 0.242 | 0.032* | 0.148 | 0.318 | -0.124 | 0.326 | -0.040 | 0.829 | -0.150 | 0.386 | -0.021 | 0.914 |
| Fusiform.L | 0.200 | 0.076 | 0.077 | 0.502 | -0.202 | 0.083 | -0.144 | 0.458 | -0.222 | 0.106 | -0.195 | 0.223 |
| InfParietal.L | 0.350 | 0.003* | 0.117 | 0.364 | -0.341 | 0.011* | -0.114 | 0.556 | -0.288 | 0.033* | -0.196 | 0.223 |
| InfTemporal.L | 0.377 | 0.001* | -0.047 | 0.685 | -0.342 | 0.011* | -0.050 | 0.789 | -0.334 | 0.014* | -0.288 | 0.045 |
| LatOcc.L | 0.335 | 0.004* | 0.101 | 0.442 | -0.287 | 0.018* | -0.086 | 0.677 | -0.308 | 0.023* | -0.082 | 0.867 |
| LatOrbFront.L | 0.207 | 0.073 | 0.209 | 0.260 | -0.295 | 0.016* | -0.160 | 0.458 | -0.048 | 0.772 | -0.012 | 0.947 |
| ParsOperc.L | 0.162 | 0.153 | 0.173 | 0.260 | -0.253 | 0.036* | -0.137 | 0.458 | -0.340 | 0.014* | -0.038 | 0.899 |
| Pericalcarine.L | 0.266 | 0.019* | 0.162 | 0.289 | -0.258 | 0.034* | -0.061 | 0.730 | -0.226 | 0.106 | 0.048 | 0.867 |
| Precentral.L | 0.143 | 0.190 | 0.092 | 0.455 | -0.019 | 0.854 | -0.142 | 0.458 | -0.010 | 0.961 | -0.202 | 0.223 |
| RosMidFront.L | 0.290 | 0.011* | 0.120 | 0.364 | -0.102 | 0.393 | 0.149 | 0.458 | -0.005 | 0.961 | 0.047 | 0.867 |
| SupFront.L | 0.152 | 0.168 | 0.219 | 0.260 | 0.149 | 0.225 | 0.219 | 0.458 | 0.083 | 0.648 | 0.299 | 0.045 |
| SupParietal.L | 0.344 | 0.003* | 0.225 | 0.260 | -0.310 | 0.012* | -0.174 | 0.458 | -0.275 | 0.040* | -0.290 | 0.045 |
| CaudMidFront.R | 0.152 | 0.168 | 0.173 | 0.260 | 0.117 | 0.344 | 0.024 | 0.886 | 0.023 | 0.903 | 0.053 | 0.867 |
| Cuneus.R | 0.356 | 0.003* | 0.138 | 0.325 | -0.247 | 0.038* | -0.002 | 0.985 | -0.061 | 0.706 | 0.069 | 0.867 |
| Fusiform.R | 0.303 | 0.008* | 0.096 | 0.452 | -0.277 | 0.022* | -0.139 | 0.458 | -0.175 | 0.264 | -0.131 | 0.588 |
| InfParietal.R | 0.377 | 0.001* | 0.086 | 0.472 | -0.309 | 0.012* | -0.099 | 0.649 | -0.139 | 0.420 | -0.148 | 0.500 |
| InfTemporal.R | 0.272 | 0.017* | 0.131 | 0.325 | -0.202 | 0.083 | -0.199 | 0.458 | -0.070 | 0.665 | -0.100 | 0.860 |
| LateralOcc.R | 0.320 | 0.005* | 0.134 | 0.325 | -0.220 | 0.065 | -0.085 | 0.677 | -0.104 | 0.577 | 0.001 | 0.992 |
| Lingual.R | 0.329 | 0.004* | 0.136 | 0.325 | -0.331 | 0.011* | -0.077 | 0.677 | -0.266 | 0.044* | -0.061 | 0.867 |
| ParsOperc.R | 0.196 | 0.079 | 0.194 | 0.260 | 0.049 | 0.694 | 0.003 | 0.985 | -0.076 | 0.656 | 0.050 | 0.867 |
| Paricalcarine.R | 0.391 | 0.001* | 0.178 | 0.260 | -0.318 | 0.012* | -0.078 | 0.677 | -0.081 | 0.648 | 0.067 | 0.867 |
| Precentral.R | 0.245 | 0.032* | 0.189 | 0.260 | 0.053 | 0.694 | -0.032 | 0.862 | 0.124 | 0.494 | 0.059 | 0.867 |
| RosMidFront.R | 0.094 | 0.372 | 0.154 | 0.310 | 0.222 | 0.065 | 0.213 | 0.458 | 0.114 | 0.536 | 0.252 | 0.097 |
| SupFront.R | 0.201 | 0.076 | 0.173 | 0.260 | 0.104 | 0.393 | 0.121 | 0.547 | NaN | NaN | NaN | NaN |
| SupParietal.R | 0.411 | 0.001* | 0.202 | 0.260 | -0.204 | 0.083 | -0.067 | 0.716 | 0.043 | 0.780 | -0.022 | 0.914 |

**Supplementary table 18: Correlation coefficients and p-values for the relationship between cognitive components and graph theory metrics of a given node within the reasoning network constructed using a 1.5 SD thresholding approach.** R=Pearson’s r, p(FDR)=p-value with FDR correction applied across nodes.

2.0 SD Units

| **GT Metrics** | Degree | | | | Local Eff | | | | Clustering Coeff | | | |
| --- | --- | --- | --- | --- | --- | --- | --- | --- | --- | --- | --- | --- |
| Node | Reasoning  R. p(FDR) | | Working mem.  R. p(FDR) | | Reasoning  R. p(FDR) | | Working mem.  R. p(FDR) | | Reasoning  R. p(FDR) | | Working mem  R. p(FDR) | |
| Cuneus.L | 0.327 | 0.003* | 0.120 | 0.329 | -0.174 | 0.127 | -0.068 | 0.713 | -0.202 | 0.138 | -0.084 | 0.719 |
| Fusiform.L | 0.249 | 0.024* | 0.131 | 0.317 | -0.273 | 0.018* | -0.201 | 0.472 | -0.268 | 0.037* | -0.237 | 0.149 |
| InfParietal.L | 0.352 | 0.002* | 0.129 | 0.317 | -0.360 | 0.006* | -0.111 | 0.472 | -0.328 | 0.027* | -0.169 | 0.350 |
| InfTemporal.L | 0.336 | 0.002* | -0.007 | 0.946 | -0.351 | 0.006* | -0.127 | 0.472 | -0.288 | 0.029* | -0.304 | 0.058 |
| LatOcc.L | 0.366 | 0.001* | 0.125 | 0.322 | -0.339 | 0.006* | -0.133 | 0.472 | -0.317 | 0.027* | -0.143 | 0.496 |
| LatOrbFront.L | 0.252 | 0.024* | 0.255 | 0.179 | -0.284 | 0.014* | -0.150 | 0.472 | -0.103 | 0.475 | -0.012 | 0.922 |
| ParsOperc.L | 0.229 | 0.036* | 0.208 | 0.179 | -0.215 | 0.060 | -0.118 | 0.472 | -0.287 | 0.029* | -0.011 | 0.922 |
| Pericalcarine.L | 0.338 | 0.002* | 0.228 | 0.179 | -0.299 | 0.010* | -0.139 | 0.472 | -0.303 | 0.029* | -0.034 | 0.887 |
| Precentral.L | 0.095 | 0.367 | 0.060 | 0.594 | 0.016 | 0.880 | -0.117 | 0.472 | 0.008 | 0.938 | -0.198 | 0.272 |
| RosMidFront.L | 0.281 | 0.011* | 0.186 | 0.204 | -0.077 | 0.549 | 0.093 | 0.547 | -0.084 | 0.525 | 0.059 | 0.788 |
| SupFront.L | 0.160 | 0.145 | 0.255 | 0.179 | 0.213 | 0.060 | 0.185 | 0.472 | 0.155 | 0.260 | 0.253 | 0.128 |
| SupParietal.L | 0.337 | 0.002* | 0.147 | 0.280 | -0.314 | 0.007* | -0.168 | 0.472 | -0.281 | 0.029* | -0.294 | 0.058 |
| CaudMidFront.R | 0.189 | 0.085 | 0.179 | 0.204 | 0.158 | 0.163 | 0.024 | 0.890 | 0.122 | 0.379 | 0.114 | 0.528 |
| Cuneus.R | 0.388 | 0.001* | 0.179 | 0.204 | -0.241 | 0.038* | -0.033 | 0.854 | -0.061 | 0.668 | 0.070 | 0.733 |
| Fusiform.R | 0.342 | 0.002* | 0.102 | 0.393 | -0.326 | 0.006* | -0.143 | 0.472 | -0.219 | 0.103 | -0.122 | 0.528 |
| InfParietal.R | 0.379 | 0.001* | 0.113 | 0.350 | -0.333 | 0.006* | -0.114 | 0.472 | -0.127 | 0.372 | -0.132 | 0.496 |
| InfTemporal.R | 0.285 | 0.011* | 0.175 | 0.204 | -0.265 | 0.021* | -0.186 | 0.472 | -0.085 | 0.525 | -0.081 | 0.719 |
| LateralOcc.R | 0.372 | 0.001* | 0.141 | 0.293 | -0.298 | 0.010* | -0.006 | 0.991 | -0.153 | 0.260 | 0.074 | 0.733 |
| Lingual.R | 0.333 | 0.003* | 0.162 | 0.244 | -0.341 | 0.006* | -0.123 | 0.472 | -0.262 | 0.038* | -0.113 | 0.528 |
| ParsOperc.R | 0.230 | 0.036* | 0.157 | 0.252 | 0.072 | 0.562 | -0.001 | 0.992 | -0.019 | 0.888 | 0.018 | 0.922 |
| Paricalcarine.R | 0.412 | 0.001* | 0.206 | 0.179 | -0.322 | 0.006* | -0.054 | 0.732 | -0.178 | 0.210 | 0.046 | 0.817 |
| Precentral.R | 0.261 | 0.019* | 0.229 | 0.179 | 0.058 | 0.629 | 0.053 | 0.732 | 0.151 | 0.260 | 0.132 | 0.496 |
| RosMidFront.R | 0.151 | 0.164 | 0.099 | 0.393 | 0.219 | 0.059 | 0.209 | 0.472 | 0.050 | 0.722 | 0.195 | 0.272 |
| SupFront.R | 0.219 | 0.044* | 0.214 | 0.179 | 0.174 | 0.127 | 0.164 | 0.472 | -0.160 | 0.260 | 0.171 | 0.350 |
| SupParietal.R | 0.411 | 0.001* | 0.197 | 0.197 | -0.235 | 0.042* | -0.098 | 0.537 | 0.032 | 0.825 | -0.054 | 0.789 |

**Supplementary table 19: Correlation coefficients and p-values for the relationship between cognitive components and graph theory metrics of a given node within the reasoning network constructed using a 2.0 SD thresholding approach.** R=Pearson’s r, p(FDR)=p-value with FDR correction applied across nodes.

2.5 SD Units

| **Reasoning network** | | | | | | | | | | | | |
| --- | --- | --- | --- | --- | --- | --- | --- | --- | --- | --- | --- | --- |
|  | **Degree** | | | | **Local Efficiency** | | | | **Clustering coefficient** | | | |
| **Node** | **Reasoning**  **R. FDR** | | **Work mem**  **R FDR** | | **Reasoning**  **R FDR** | | **Work mem**  **R FDR** | | **Reasoning**  **R FDR** | | **Work mem**  **R FDR** | |
| CaudMidFront.L | 0.141 | 0.193 | 0.095 | 0.398 | 0.035 | 0.800 | -0.078 | 0.665 | -0.022 | 0.838 | -0.060 | 0.752 |
| Cuneus.L | 0.331 | 0.003** | 0.107 | 0.366 | -0.334 | 0.004** | -0.036 | 0.829 | -0.332 | 0.010* | -0.059 | 0.752 |
| Fusiform.L | 0.299 | 0.007** | 0.164 | 0.219 | -0.334 | 0.004** | -0.251 | 0.207 | -0.321 | 0.010* | -0.258 | 0.112 |
| InfParietal.L | 0.338 | 0.003** | 0.120 | 0.329 | -0.347 | 0.004** | -0.123 | 0.444 | -0.317 | 0.010* | -0.177 | 0.328 |
| InfTemporal.L | 0.288 | 0.009** | 0.070 | 0.526 | -0.337 | 0.004** | -0.176 | 0.444 | -0.339 | 0.010* | -0.310 | 0.068 |
| LatOcc.L | 0.369 | 0.002** | 0.122 | 0.329 | -0.331 | 0.004** | -0.142 | 0.444 | -0.314 | 0.010* | -0.177 | 0.328 |
| LatOrbFront.L | 0.245 | 0.024* | 0.238 | 0.136 | -0.208 | 0.064 | -0.158 | 0.444 | -0.087 | 0.534 | -0.006 | 0.958 |
| ParsOperc.L | 0.308 | 0.005** | 0.187 | 0.160 | -0.236 | 0.034* | -0.116 | 0.444 | -0.286 | 0.019* | -0.042 | 0.812 |
| Pericalcarine.L | 0.355 | 0.002** | 0.211 | 0.142 | -0.367 | 0.004** | -0.111 | 0.444 | -0.322 | 0.010* | -0.038 | 0.812 |
| Precentral.L | 0.085 | 0.420 | 0.066 | 0.534 | 0.005 | 0.959 | -0.113 | 0.444 | 0.025 | 0.838 | -0.172 | 0.328 |
| RosMidFront.L^✝^ | 0.280 | 0.010* | 0.198 | 0.151 | -0.020 | 0.885 | 0.032 | 0.829 | -0.022 | 0.838 | -0.076 | 0.718 |
| SupFront.L^✝^ | 0.195 | 0.071 | 0.256 | 0.136 | 0.284 | 0.012* | 0.136 | 0.444 | 0.242 | 0.058 | 0.193 | 0.328 |
| SupParietal.L^✝^ | 0.345 | 0.002** | 0.130 | 0.311 | -0.338 | 0.004** | -0.141 | 0.444 | -0.300 | 0.014* | -0.261 | 0.112 |
| CaudMidFront.R | 0.230 | 0.032* | 0.224 | 0.136 | 0.134 | 0.240 | 0.011 | 0.941 | 0.112 | 0.495 | 0.160 | 0.337 |
| Cuneus.R | 0.439 | 0.000*** | 0.180 | 0.174 | -0.305 | 0.007** | -0.038 | 0.829 | -0.081 | 0.547 | 0.061 | 0.752 |
| Fusiform.R | 0.347 | 0.002** | 0.151 | 0.250 | -0.321 | 0.005** | -0.159 | 0.444 | -0.214 | 0.087 | -0.109 | 0.558 |
| InfParietal.R^✝^ | 0.366 | 0.002** | 0.139 | 0.285 | -0.329 | 0.004** | -0.160 | 0.444 | -0.094 | 0.534 | -0.137 | 0.387 |
| InfTemporal.R | 0.331 | 0.003** | 0.191 | 0.160 | -0.272 | 0.014* | -0.253 | 0.207 | -0.087 | 0.534 | -0.148 | 0.346 |
| LateralOcc.R | 0.327 | 0.003** | 0.113 | 0.354 | -0.278 | 0.013* | -0.034 | 0.829 | -0.139 | 0.344 | 0.025 | 0.881 |
| Lingual.R | 0.358 | 0.002** | 0.203 | 0.151 | -0.311 | 0.006** | -0.155 | 0.444 | -0.219 | 0.085 | -0.099 | 0.605 |
| ParsOperc.R | 0.257 | 0.018* | 0.230 | 0.136 | 0.136 | 0.240 | 0.008 | 0.941 | 0.108 | 0.495 | 0.078 | 0.718 |
| Paricalcarine.R | 0.419 | 0.000*** | 0.150 | 0.250 | -0.314 | 0.006** | -0.055 | 0.787 | -0.159 | 0.262 | 0.013 | 0.939 |
| Precentral.R | 0.233 | 0.032* | 0.250 | 0.136 | 0.085 | 0.476 | 0.061 | 0.769 | 0.230 | 0.072 | 0.155 | 0.337 |
| RosMidFront.R | 0.137 | 0.202 | 0.095 | 0.398 | 0.252 | 0.024* | 0.215 | 0.344 | 0.074 | 0.568 | 0.179 | 0.328 |
| SupFront.R | 0.259 | 0.018* | 0.269 | 0.136 | 0.204 | 0.067 | 0.114 | 0.444 | -0.095 | 0.534 | 0.154 | 0.337 |
| SupParietal.R^✝^ | 0.436 | 0.000*** | 0.212 | 0.142 | -0.277 | 0.013* | -0.121 | 0.444 | 0.030 | 0.838 | -0.041 | 0.812 |

**Supplementary table 20: Correlation coefficients and p-values for the relationship between cognitive components and graph theory metrics of a given node within the reasoning network constructed using a 2.5 SD thresholding approach.** R=Pearson’s r, p(FDR)=p-value with FDR correction applied across nodes.

1. SD Units

| **GT Metrics** | Degree | | | | Local Eff | | | | Clustering Coeff | | | |
| --- | --- | --- | --- | --- | --- | --- | --- | --- | --- | --- | --- | --- |
| Node | Reasoning  R. p(FDR) | | Working mem.  R. p(FDR) | | Reasoning  R. p(FDR) | | Working mem.  R. p(FDR) | | Reasoning  R. p(FDR) | | Working mem.  R. p(FDR) | |
| CaudMidFront.L | 0.208 | 0.050 | 0.113 | 0.337 | 0.120 | 0.301 | -0.034 | 0.806 | 0.057 | 0.641 | -0.074 | 0.702 |
| Cuneus.L | 0.336 | 0.002* | 0.107 | 0.348 | -0.337 | 0.003* | -0.064 | 0.746 | -0.330 | 0.006* | -0.064 | 0.740 |
| Fusiform.L | 0.342 | 0.002* | 0.194 | 0.187 | -0.371 | 0.003* | -0.221 | 0.383 | -0.343 | 0.006* | -0.221 | 0.315 |
| InfParietal.L | 0.350 | 0.002* | 0.141 | 0.236 | -0.351 | 0.003* | -0.125 | 0.383 | -0.333 | 0.006* | -0.154 | 0.449 |
| InfTemporal.L | 0.302 | 0.005* | 0.075 | 0.477 | -0.374 | 0.003* | -0.204 | 0.383 | -0.336 | 0.006* | -0.296 | 0.108 |
| LatOcc.L | 0.356 | 0.001* | 0.146 | 0.236 | -0.342 | 0.003* | -0.128 | 0.383 | -0.344 | 0.006* | -0.124 | 0.515 |
| LatOrbFront.L | 0.259 | 0.014* | 0.269 | 0.100 | -0.171 | 0.128 | -0.143 | 0.383 | -0.047 | 0.685 | 0.040 | 0.767 |
| ParsOperc.L | 0.265 | 0.013* | 0.142 | 0.236 | -0.089 | 0.431 | -0.168 | 0.383 | -0.179 | 0.152 | -0.079 | 0.697 |
| Pericalcarine.L | 0.332 | 0.002* | 0.169 | 0.206 | -0.368 | 0.003* | -0.100 | 0.524 | -0.346 | 0.006* | -0.047 | 0.740 |
| Precentral.L | 0.103 | 0.331 | 0.090 | 0.410 | 0.036 | 0.760 | -0.132 | 0.383 | 0.074 | 0.546 | -0.166 | 0.426 |
| RosMidFront.L | 0.279 | 0.010* | 0.184 | 0.187 | -0.017 | 0.875 | 0.069 | 0.746 | -0.129 | 0.318 | -0.050 | 0.740 |
| SupFront.L | 0.202 | 0.056 | 0.251 | 0.100 | 0.320 | 0.005* | 0.137 | 0.383 | 0.274 | 0.021* | 0.192 | 0.315 |
| SupParietal.L | 0.366 | 0.001* | 0.151 | 0.236 | -0.359 | 0.003* | -0.156 | 0.383 | -0.285 | 0.017* | -0.214 | 0.315 |
| CaudMidFront.R | 0.276 | 0.010* | 0.245 | 0.100 | 0.200 | 0.077 | 0.038 | 0.806 | 0.201 | 0.110 | 0.149 | 0.449 |
| Cuneus.R | 0.396 | 0.001* | 0.147 | 0.236 | -0.280 | 0.013* | -0.028 | 0.825 | -0.080 | 0.546 | 0.050 | 0.740 |
| Fusiform.R | 0.405 | 0.001* | 0.184 | 0.187 | -0.316 | 0.005* | -0.145 | 0.383 | -0.204 | 0.110 | -0.109 | 0.525 |
| InfParietal.R | 0.369 | 0.001* | 0.126 | 0.285 | -0.294 | 0.009* | -0.155 | 0.383 | -0.005 | 0.965 | -0.132 | 0.515 |
| InfTemporal.R | 0.335 | 0.002* | 0.167 | 0.206 | -0.270 | 0.016* | -0.244 | 0.383 | -0.076 | 0.546 | -0.126 | 0.515 |
| LateralOcc.R | 0.345 | 0.002* | 0.094 | 0.406 | -0.314 | 0.005* | -0.023 | 0.825 | -0.221 | 0.082 | 0.028 | 0.821 |
| Lingual.R | 0.358 | 0.001* | 0.192 | 0.187 | -0.356 | 0.003* | -0.155 | 0.383 | -0.296 | 0.014* | -0.109 | 0.525 |
| ParsOperc.R | 0.306 | 0.005* | 0.228 | 0.126 | 0.186 | 0.098 | 0.057 | 0.765 | 0.176 | 0.152 | 0.084 | 0.689 |
| Paricalcarine.R | 0.413 | 0.001* | 0.156 | 0.236 | -0.307 | 0.006* | -0.045 | 0.806 | -0.170 | 0.160 | 0.016 | 0.876 |
| Precentral.R | 0.280 | 0.010* | 0.256 | 0.100 | 0.116 | 0.306 | 0.039 | 0.806 | 0.307 | 0.011* | 0.114 | 0.525 |
| RosMidFront.R | 0.268 | 0.012* | 0.179 | 0.190 | 0.243 | 0.032* | 0.158 | 0.383 | 0.183 | 0.150 | 0.192 | 0.315 |
| SupFront.R | 0.355 | 0.001* | 0.244 | 0.100 | 0.222 | 0.048* | 0.126 | 0.383 | 0.081 | 0.546 | 0.188 | 0.315 |
| SupParietal.R | 0.423 | 0.001* | 0.220 | 0.129 | -0.233 | 0.039* | -0.151 | 0.383 | 0.101 | 0.463 | -0.050 | 0.740 |

**Supplementary table 21: Correlation coefficients and p-values for the relationship between cognitive components and graph theory metrics of a given node within the reasoning network constructed using a 3.0 SD thresholding approach.** R=Pearson’s r, p(FDR)=p-value with FDR correction applied across nodes.

3.5 SD Units

| **GT Metrics** | Degree | | | | Local Eff | | | | Clustering Coeff | | | |
| --- | --- | --- | --- | --- | --- | --- | --- | --- | --- | --- | --- | --- |
| Node | Reasoning  R. p(FDR) | | Working mem.  R. p(FDR) | | Reasoning  R. p(FDR) | | Working mem.  R. p(FDR) | | Reasoning  R. p(FDR) | | Working mem.  R. p(FDR) | |
| CaudMidFront.L | 0.219 | 0.040* | 0.141 | 0.262 | 0.135 | 0.236 | 0.077 | 0.595 | 0.118 | 0.326 | 0.040 | 0.837 |
| Cuneus.L | 0.330 | 0.003* | 0.092 | 0.394 | -0.340 | 0.002* | -0.074 | 0.595 | -0.354 | 0.005* | -0.061 | 0.771 |
| Fusiform.L | 0.325 | 0.003* | 0.208 | 0.157 | -0.378 | 0.002* | -0.225 | 0.405 | -0.365 | 0.005* | -0.224 | 0.279 |
| InfParietal.L | 0.312 | 0.004* | 0.104 | 0.373 | -0.328 | 0.003* | -0.096 | 0.554 | -0.329 | 0.006* | -0.137 | 0.454 |
| InfTemporal.L | 0.306 | 0.004* | 0.100 | 0.373 | -0.346 | 0.002* | -0.202 | 0.405 | -0.315 | 0.007* | -0.292 | 0.123 |
| LatOcc.L | 0.337 | 0.003* | 0.142 | 0.262 | -0.330 | 0.003* | -0.108 | 0.528 | -0.344 | 0.005* | -0.072 | 0.715 |
| LatOrbFront.L | 0.214 | 0.044* | 0.267 | 0.052 | -0.012 | 0.908 | -0.113 | 0.528 | 0.108 | 0.360 | 0.045 | 0.834 |
| ParsOperc.L | 0.306 | 0.004* | 0.189 | 0.186 | -0.038 | 0.782 | -0.180 | 0.405 | -0.169 | 0.171 | -0.110 | 0.511 |
| Pericalcarine.L | 0.332 | 0.003* | 0.182 | 0.196 | -0.364 | 0.002* | -0.099 | 0.554 | -0.347 | 0.005* | -0.021 | 0.876 |
| Precentral.L | 0.089 | 0.401 | 0.090 | 0.394 | 0.018 | 0.897 | -0.130 | 0.473 | 0.074 | 0.526 | -0.169 | 0.319 |
| RosMidFront.L | 0.267 | 0.013* | 0.152 | 0.258 | 0.138 | 0.234 | 0.023 | 0.825 | 0.085 | 0.478 | -0.055 | 0.781 |
| SupFront.L | 0.204 | 0.053 | 0.233 | 0.111 | 0.350 | 0.002* | 0.163 | 0.405 | 0.315 | 0.007* | 0.168 | 0.319 |
| SupParietal.L | 0.372 | 0.001* | 0.148 | 0.258 | -0.376 | 0.002* | -0.164 | 0.405 | -0.312 | 0.007* | -0.224 | 0.279 |
| CaudMidFront.R | 0.264 | 0.013* | 0.288 | 0.047* | 0.207 | 0.065 | 0.063 | 0.618 | 0.220 | 0.066 | 0.191 | 0.304 |
| Cuneus.R | 0.392 | 0.001* | 0.133 | 0.280 | -0.280 | 0.013* | -0.039 | 0.743 | -0.122 | 0.319 | 0.026 | 0.875 |
| Fusiform.R | 0.434 | 0.000* | 0.206 | 0.157 | -0.365 | 0.002* | -0.173 | 0.405 | -0.246 | 0.036* | -0.128 | 0.473 |
| InfParietal.R | 0.363 | 0.001* | 0.149 | 0.258 | -0.272 | 0.015* | -0.152 | 0.405 | 0.011 | 0.918 | -0.106 | 0.511 |
| InfTemporal.R | 0.336 | 0.003* | 0.165 | 0.252 | -0.305 | 0.006* | -0.275 | 0.208 | -0.123 | 0.319 | -0.182 | 0.304 |
| LateralOcc.R | 0.358 | 0.001* | 0.100 | 0.373 | -0.357 | 0.002* | -0.060 | 0.618 | -0.300 | 0.009* | -0.012 | 0.908 |
| Lingual.R | 0.335 | 0.003* | 0.130 | 0.280 | -0.356 | 0.002* | -0.144 | 0.405 | -0.311 | 0.007* | -0.120 | 0.473 |
| ParsOperc.R | 0.290 | 0.007* | 0.267 | 0.052 | 0.246 | 0.029* | 0.077 | 0.595 | 0.200 | 0.098 | 0.123 | 0.473 |
| Paricalcarine.R | 0.390 | 0.001* | 0.124 | 0.298 | -0.348 | 0.002* | -0.073 | 0.595 | -0.262 | 0.025* | -0.032 | 0.860 |
| Precentral.R | 0.242 | 0.024* | 0.289 | 0.047* | 0.125 | 0.268 | 0.071 | 0.595 | 0.335 | 0.006* | 0.146 | 0.426 |
| RosMidFront.R | 0.308 | 0.004* | 0.151 | 0.258 | 0.223 | 0.050 | 0.145 | 0.405 | 0.130 | 0.311 | 0.185 | 0.304 |
| SupFront.R | 0.389 | 0.001* | 0.292 | 0.047* | 0.210 | 0.065 | 0.110 | 0.528 | 0.059 | 0.597 | 0.205 | 0.304 |
| SupParietal.R | 0.419 | 0.000* | 0.189 | 0.186 | -0.166 | 0.147 | -0.158 | 0.405 | 0.167 | 0.171 | -0.078 | 0.699 |

**Supplementary table 22: Correlation coefficients and p-values for the relationship between cognitive components and graph theory metrics of a given node within the reasoning network constructed using a 3.5 SD thresholding approach.** R=Pearson’s r, p(FDR)=p-value with FDR correction applied across nodes.

1. SD Units

| **GT Metrics** | Degree | | | | Local Eff | | | | Clustering Coeff | | | |
| --- | --- | --- | --- | --- | --- | --- | --- | --- | --- | --- | --- | --- |
| Node | Reasoning  R. p(FDR) | | Working mem.  R. p(FDR) | | Reasoning  R. p(FDR) | | Working mem.  R. p(FDR) | | Reasoning  R. p(FDR) | | Working mem.  R. p(FDR) | |
| CaudMidFront.L | 0.250 | 0.019* | 0.130 | 0.331 | 0.170 | 0.125 | 0.066 | 0.670 | 0.193 | 0.099 | 0.040 | 0.877 |
| Cuneus.L | 0.316 | 0.004* | 0.083 | 0.491 | -0.341 | 0.003* | -0.063 | 0.670 | -0.341 | 0.004* | -0.029 | 0.878 |
| Fusiform.L | 0.361 | 0.002* | 0.178 | 0.212 | -0.407 | 0.001* | -0.233 | 0.333 | -0.357 | 0.004* | -0.245 | 0.314 |
| InfParietal.L | 0.325 | 0.003* | 0.100 | 0.448 | -0.291 | 0.009* | -0.103 | 0.646 | -0.255 | 0.034* | -0.158 | 0.360 |
| InfTemporal.L | 0.292 | 0.007* | 0.082 | 0.491 | -0.345 | 0.002* | -0.164 | 0.512 | -0.337 | 0.004* | -0.235 | 0.314 |
| LatOcc.L | 0.303 | 0.006* | 0.089 | 0.491 | -0.326 | 0.004* | -0.088 | 0.670 | -0.347 | 0.004* | -0.059 | 0.805 |
| LatOrbFront.L | 0.238 | 0.025* | 0.288 | 0.061 | 0.049 | 0.698 | -0.155 | 0.512 | 0.161 | 0.162 | 0.040 | 0.877 |
| ParsOperc.L | 0.288 | 0.007* | 0.172 | 0.218 | -0.013 | 0.903 | -0.166 | 0.512 | -0.159 | 0.162 | -0.156 | 0.360 |
| Pericalcarine.L | 0.329 | 0.003* | 0.135 | 0.321 | -0.366 | 0.002* | -0.074 | 0.670 | -0.369 | 0.004* | -0.030 | 0.878 |
| Precentral.L | 0.128 | 0.226 | 0.074 | 0.506 | 0.041 | 0.724 | -0.099 | 0.646 | 0.113 | 0.322 | -0.144 | 0.404 |
| RosMidFront.L | 0.290 | 0.007* | 0.202 | 0.181 | 0.082 | 0.493 | -0.004 | 0.972 | 0.028 | 0.790 | 0.011 | 0.917 |
| SupFront.L | 0.173 | 0.103 | 0.195 | 0.181 | 0.396 | 0.001* | 0.130 | 0.564 | 0.305 | 0.009* | 0.160 | 0.360 |
| SupParietal.L | 0.343 | 0.002* | 0.159 | 0.240 | -0.365 | 0.002* | -0.171 | 0.512 | -0.318 | 0.007* | -0.200 | 0.335 |
| CaudMidFront.R | 0.258 | 0.016* | 0.303 | 0.061 | 0.205 | 0.069 | 0.069 | 0.670 | 0.208 | 0.076 | 0.194 | 0.335 |
| Cuneus.R | 0.376 | 0.001* | 0.123 | 0.334 | -0.316 | 0.004* | -0.044 | 0.729 | -0.176 | 0.126 | 0.025 | 0.878 |
| Fusiform.R | 0.448 | 0.000* | 0.203 | 0.181 | -0.369 | 0.002* | -0.157 | 0.512 | -0.241 | 0.040* | -0.107 | 0.539 |
| InfParietal.R | 0.347 | 0.002* | 0.137 | 0.321 | -0.234 | 0.038* | -0.115 | 0.646 | 0.034 | 0.780 | -0.093 | 0.613 |
| InfTemporal.R | 0.353 | 0.002* | 0.164 | 0.235 | -0.334 | 0.003* | -0.249 | 0.333 | -0.181 | 0.122 | -0.162 | 0.360 |
| LateralOcc.R | 0.337 | 0.002* | 0.061 | 0.560 | -0.346 | 0.002* | -0.066 | 0.670 | -0.310 | 0.009* | -0.057 | 0.805 |
| Lingual.R | 0.377 | 0.001* | 0.124 | 0.334 | -0.381 | 0.002* | -0.142 | 0.512 | -0.301 | 0.009* | -0.121 | 0.524 |
| ParsOperc.R | 0.295 | 0.007* | 0.270 | 0.061 | 0.272 | 0.015* | 0.057 | 0.670 | 0.239 | 0.040* | 0.109 | 0.539 |
| Paricalcarine.R | 0.347 | 0.002* | 0.079 | 0.491 | -0.320 | 0.004* | -0.041 | 0.729 | -0.251 | 0.034* | -0.018 | 0.896 |
| Precentral.R | 0.220 | 0.038* | 0.266 | 0.061 | 0.179 | 0.114 | 0.057 | 0.670 | 0.339 | 0.004* | 0.118 | 0.524 |
| RosMidFront.R | 0.282 | 0.008* | 0.184 | 0.204 | 0.240 | 0.035* | 0.109 | 0.646 | 0.137 | 0.226 | 0.208 | 0.335 |
| SupFront.R | 0.385 | 0.001* | 0.262 | 0.061 | 0.229 | 0.041* | 0.082 | 0.670 | 0.067 | 0.566 | 0.172 | 0.360 |
| SupParietal.R | 0.419 | 0.000* | 0.195 | 0.181 | -0.173 | 0.122 | -0.143 | 0.512 | 0.215 | 0.068 | -0.061 | 0.805 |

**Supplementary table 23: Correlation coefficients and p-values for the relationship between cognitive components and graph theory metrics of a given node within the reasoning network constructed using a 4.0 SD thresholding approach.** R=Pearson’s r, p(FDR)=p-value with FDR correction applied across nodes.

4.5 SD Units

| **GT Metrics** | Degree | | | | Local Eff | | | | Clustering Coeff | | | |
| --- | --- | --- | --- | --- | --- | --- | --- | --- | --- | --- | --- | --- |
| Node | Reasoning  R. p(FDR) | | Working mem.  R. p(FDR) | | Reasoning  R. p(FDR) | | Working mem.  R. p(FDR) | | Reasoning  R. p(FDR) | | Working mem.  R. p(FDR) | |
| CaudMidFront.L | 0.273 | 0.012* | 0.092 | 0.495 | 0.219 | 0.047* | 0.075 | 0.669 | 0.274 | 0.018* | 0.010 | 0.962 |
| Cuneus.L | 0.312 | 0.004* | 0.072 | 0.538 | -0.334 | 0.003* | -0.069 | 0.669 | -0.348 | 0.005* | -0.051 | 0.854 |
| Fusiform.L | 0.342 | 0.002* | 0.142 | 0.308 | -0.378 | 0.003* | -0.206 | 0.364 | -0.325 | 0.006* | -0.228 | 0.344 |
| InfParietal.L | 0.311 | 0.004* | 0.076 | 0.532 | -0.270 | 0.015* | -0.093 | 0.669 | -0.255 | 0.026* | -0.171 | 0.344 |
| InfTemporal.L | 0.265 | 0.014* | 0.094 | 0.495 | -0.340 | 0.003* | -0.180 | 0.364 | -0.352 | 0.005* | -0.236 | 0.344 |
| LatOcc.L | 0.319 | 0.004* | 0.089 | 0.496 | -0.333 | 0.003* | -0.095 | 0.669 | -0.368 | 0.005* | -0.074 | 0.789 |
| LatOrbFront.L | 0.228 | 0.033* | 0.282 | 0.050 | 0.030 | 0.808 | -0.167 | 0.364 | 0.191 | 0.093 | -0.020 | 0.958 |
| ParsOperc.L | 0.258 | 0.016* | 0.157 | 0.271 | 0.002 | 0.987 | -0.207 | 0.364 | -0.174 | 0.114 | -0.153 | 0.344 |
| Pericalcarine.L | 0.330 | 0.003* | 0.120 | 0.413 | -0.348 | 0.003* | -0.072 | 0.669 | -0.341 | 0.005* | -0.037 | 0.902 |
| Precentral.L | 0.110 | 0.295 | 0.085 | 0.496 | 0.038 | 0.776 | -0.106 | 0.669 | 0.102 | 0.349 | -0.136 | 0.425 |
| RosMidFront.L | 0.290 | 0.007* | 0.201 | 0.145 | 0.096 | 0.412 | -0.020 | 0.847 | 0.077 | 0.468 | 0.047 | 0.854 |
| SupFront.L | 0.181 | 0.087 | 0.202 | 0.145 | 0.400 | 0.002* | 0.133 | 0.540 | 0.328 | 0.006* | 0.158 | 0.344 |
| SupParietal.L | 0.345 | 0.002* | 0.151 | 0.280 | -0.367 | 0.003* | -0.175 | 0.364 | -0.308 | 0.009* | -0.201 | 0.344 |
| CaudMidFront.R | 0.214 | 0.044* | 0.319 | 0.050 | 0.242 | 0.028* | 0.077 | 0.669 | 0.241 | 0.034* | 0.183 | 0.344 |
| Cuneus.R | 0.379 | 0.001* | 0.109 | 0.439 | -0.319 | 0.005* | -0.051 | 0.720 | -0.211 | 0.064 | -0.001 | 0.993 |
| Fusiform.R | 0.418 | 0.000* | 0.227 | 0.128 | -0.336 | 0.003* | -0.173 | 0.364 | -0.241 | 0.034* | -0.116 | 0.539 |
| InfParietal.R | 0.380 | 0.001* | 0.159 | 0.271 | -0.241 | 0.028* | -0.133 | 0.540 | 0.109 | 0.328 | -0.029 | 0.923 |
| InfTemporal.R | 0.339 | 0.002* | 0.165 | 0.271 | -0.314 | 0.005* | -0.242 | 0.364 | -0.174 | 0.114 | -0.157 | 0.344 |
| LateralOcc.R | 0.327 | 0.003* | 0.026 | 0.806 | -0.334 | 0.003* | -0.041 | 0.738 | -0.305 | 0.009* | -0.065 | 0.827 |
| Lingual.R | 0.383 | 0.001* | 0.108 | 0.439 | -0.351 | 0.003* | -0.114 | 0.660 | -0.262 | 0.023* | -0.096 | 0.632 |
| ParsOperc.R | 0.343 | 0.002* | 0.259 | 0.066 | 0.297 | 0.008* | 0.039 | 0.738 | 0.300 | 0.009* | 0.101 | 0.628 |
| Paricalcarine.R | 0.307 | 0.005* | 0.057 | 0.615 | -0.296 | 0.008* | -0.050 | 0.720 | -0.281 | 0.016* | -0.052 | 0.854 |
| Precentral.R | 0.230 | 0.033* | 0.276 | 0.050 | 0.149 | 0.184 | 0.090 | 0.669 | 0.343 | 0.005* | 0.157 | 0.344 |
| RosMidFront.R | 0.302 | 0.005* | 0.200 | 0.145 | 0.287 | 0.010* | 0.052 | 0.720 | 0.185 | 0.102 | 0.158 | 0.344 |
| SupFront.R | 0.360 | 0.002* | 0.298 | 0.050 | 0.260 | 0.019* | 0.081 | 0.669 | 0.171 | 0.117 | 0.205 | 0.344 |
| SupParietal.R | 0.428 | 0.000* | 0.219 | 0.132 | -0.193 | 0.081 | -0.181 | 0.364 | 0.223 | 0.049* | -0.013 | 0.962 |

**Supplementary table 24: Correlation coefficients and p-values for the relationship between cognitive components and graph theory metrics of a given node within the reasoning network constructed using a 4.5 SD thresholding approach.** R=Pearson’s r, p(FDR)=p-value with FDR correction applied across nodes.

5.0 SD Units

| **GT Metrics** | Degree | | | | Local Eff | | | | Clustering Coeff | | | |
| --- | --- | --- | --- | --- | --- | --- | --- | --- | --- | --- | --- | --- |
| Node | Reasoning  R. p(FDR) | | Working mem.  R. p(FDR) | | Reasoning  R. p(FDR) | | Working mem.  R. p(FDR) | | Reasoning  R. p(FDR) | | Working mem.  R. p(FDR) | |
| CaudMidFront.L | 0.212 | 0.048* | 0.066 | 0.606 | 0.230 | 0.040* | 0.064 | 0.656 | 0.243 | 0.032* | -0.037 | 0.859 |
| Cuneus.L | 0.304 | 0.005* | 0.062 | 0.606 | -0.318 | 0.005* | -0.070 | 0.656 | -0.335 | 0.007* | -0.063 | 0.731 |
| Fusiform.L | 0.350 | 0.003* | 0.144 | 0.344 | -0.377 | 0.003* | -0.203 | 0.395 | -0.318 | 0.007* | -0.207 | 0.435 |
| InfParietal.L | 0.309 | 0.005* | 0.065 | 0.606 | -0.272 | 0.013* | -0.112 | 0.632 | -0.240 | 0.033* | -0.194 | 0.435 |
| InfTemporal.L | 0.262 | 0.015* | 0.076 | 0.587 | -0.356 | 0.003* | -0.186 | 0.395 | -0.331 | 0.007* | -0.230 | 0.435 |
| LatOcc.L | 0.303 | 0.005* | 0.083 | 0.564 | -0.326 | 0.004* | -0.084 | 0.656 | -0.357 | 0.007* | -0.071 | 0.726 |
| LatOrbFront.L | 0.231 | 0.033* | 0.303 | 0.048* | 0.055 | 0.655 | -0.211 | 0.395 | 0.285 | 0.014* | -0.071 | 0.726 |
| ParsOperc.L | 0.280 | 0.010* | 0.171 | 0.222 | 0.035 | 0.740 | -0.150 | 0.503 | -0.171 | 0.116 | -0.125 | 0.515 |
| Pericalcarine.L | 0.342 | 0.003* | 0.115 | 0.420 | -0.344 | 0.003* | -0.070 | 0.656 | -0.324 | 0.007* | -0.022 | 0.938 |
| Precentral.L | 0.125 | 0.235 | 0.109 | 0.436 | 0.051 | 0.655 | -0.106 | 0.632 | 0.151 | 0.162 | -0.110 | 0.515 |
| RosMidFront.L | 0.307 | 0.005* | 0.232 | 0.078 | 0.167 | 0.146 | -0.018 | 0.863 | 0.231 | 0.036* | 0.123 | 0.515 |
| SupFront.L | 0.184 | 0.082 | 0.194 | 0.152 | 0.388 | 0.003* | 0.131 | 0.553 | 0.316 | 0.007* | 0.127 | 0.515 |
| SupParietal.L | 0.324 | 0.004* | 0.135 | 0.346 | -0.352 | 0.003* | -0.161 | 0.468 | -0.316 | 0.007* | -0.181 | 0.435 |
| CaudMidFront.R | 0.187 | 0.080 | 0.256 | 0.078 | 0.219 | 0.049* | 0.079 | 0.656 | 0.214 | 0.049* | 0.127 | 0.515 |
| Cuneus.R | 0.362 | 0.002* | 0.105 | 0.436 | -0.299 | 0.008* | -0.049 | 0.730 | -0.213 | 0.049* | -0.018 | 0.938 |
| Fusiform.R | 0.406 | 0.001* | 0.226 | 0.078 | -0.337 | 0.003* | -0.172 | 0.435 | -0.244 | 0.032* | -0.113 | 0.515 |
| InfParietal.R | 0.359 | 0.002* | 0.136 | 0.346 | -0.160 | 0.157 | -0.074 | 0.656 | 0.103 | 0.329 | 0.002 | 0.988 |
| InfTemporal.R | 0.364 | 0.002* | 0.231 | 0.078 | -0.275 | 0.013* | -0.236 | 0.395 | -0.132 | 0.217 | -0.139 | 0.515 |
| LateralOcc.R | 0.332 | 0.004* | 0.016 | 0.877 | -0.341 | 0.003* | -0.035 | 0.773 | -0.300 | 0.010* | -0.057 | 0.731 |
| Lingual.R | 0.319 | 0.004* | 0.115 | 0.420 | -0.342 | 0.003* | -0.109 | 0.632 | -0.277 | 0.015* | -0.077 | 0.726 |
| ParsOperc.R | 0.293 | 0.007* | 0.287 | 0.048* | 0.311 | 0.006* | 0.065 | 0.656 | 0.297 | 0.010* | 0.119 | 0.515 |
| Paricalcarine.R | 0.303 | 0.005* | 0.057 | 0.615 | -0.306 | 0.007* | -0.062 | 0.656 | -0.281 | 0.015* | -0.060 | 0.731 |
| Precentral.R | 0.212 | 0.048* | 0.229 | 0.078 | 0.101 | 0.381 | 0.134 | 0.553 | 0.323 | 0.007* | 0.170 | 0.455 |
| RosMidFront.R | 0.321 | 0.004* | 0.232 | 0.078 | 0.293 | 0.008* | 0.042 | 0.746 | 0.224 | 0.042* | 0.156 | 0.512 |
| SupFront.R | 0.347 | 0.003* | 0.297 | 0.048* | 0.297 | 0.008* | 0.078 | 0.656 | 0.254 | 0.027* | 0.186 | 0.435 |
| SupParietal.R | 0.404 | 0.001* | 0.243 | 0.078 | -0.137 | 0.230 | -0.194 | 0.395 | 0.234 | 0.036* | -0.009 | 0.966 |

**Supplementary table 25: Correlation coefficients and p-values for the relationship between cognitive components and graph theory metrics of a given node within the reasoning network constructed using a 5.0 SD thresholding approach.** R=Pearson’s r, p(FDR)=p-value with FDR correction applied across nodes.

**References**

Latora V, Marchiori M. Efficient behavior of small-world networks. Phys Rev Lett 2001, 87(19), 198-701.

Onnela JP, Saramäki J, Kertész J, Kaski K. Intensity and coherence of motifs in weighted complex networks. Phys Rev E Stat Nonlin Soft Matter Phys 2005; 71(6 Pt 2): 065103.

Klados MA, Kanatsouli K, Antoniou I, Babiloni F, Tsirka V, Bamidis PD, Micheloyannis S. A Graph theoretical approach to study the organization of the cortical networks during different mathematical tasks. PLoS One 2013; 8(8): e71800.

Sporns O, Chialvo DR, Kaiser M, Hilgetag CC. Organization, development and function of complex brain networks. Trends Cogn Sci. 2004;8(9):418-25.

Chen Z, Liu M, Gross DW, Beaulieu C. Graph theoretical analysis of developmental patterns of the white matter network. Front Hum Neurosci. 2013;7.

Göttlich M, Münte TF, Heldmann M, Kasten M, Hagenah J, Krämer UM. Altered Resting State Brain Networks in Parkinson’s Disease. PLoS One. 2013;8(10):e77336.
